# The Variation of Transcriptomic Perturbations is Associated with the Development and Progression of Various Diseases

**DOI:** 10.1155/2022/2148627

**Published:** 2022-09-26

**Authors:** Zehua Dong, Qiyu Yan, Xiaosheng Wang

**Affiliations:** ^1^Biomedical Informatics Research Laboratory, School of Basic Medicine and Clinical Pharmacy, China Pharmaceutical University, Nanjing 211198, China; ^2^Big Data Research Institute, China Pharmaceutical University, Nanjing 211198, China

## Abstract

**Background:**

Although transcriptomic data have been widely applied to explore various diseases, few studies have investigated the association between transcriptomic perturbations and disease development in a wide variety of diseases.

**Methods:**

Based on a previously developed algorithm for quantifying intratumor heterogeneity at the transcriptomic level, we defined the variation of transcriptomic perturbations (VTP) of a disease relative to the health status. Based on publicly available transcriptome datasets, we compared VTP values between the disease and health status and analyzed correlations between VTP values and disease progression or severity in various diseases, including neurological disorders, infectious diseases, cardiovascular diseases, respiratory diseases, liver diseases, kidney diseases, digestive diseases, and endocrine diseases. We also identified the genes and pathways whose expression perturbations correlated positively with VTP across diverse diseases.

**Results:**

VTP values were upregulated in various diseases relative to their normal controls. VTP values were significantly greater in define than in possible or probable Alzheimer's disease. VTP values were significantly larger in intensive care unit (ICU) COVID-19 patients than in non-ICU patients, and in COVID-19 patients requiring mechanical ventilatory support (MVS) than in those not requiring MVS. VTP correlated positively with viral loads in acquired immune deficiency syndrome (AIDS) patients. Moreover, the AIDS patients treated with abacavir or zidovudine had lower VTP values than those without such therapies. In pulmonary tuberculosis (TB) patients, VTP values followed the pattern: active TB > latent TB > normal controls. VTP values were greater in clinically apparent than in presymptomatic malaria. VTP correlated negatively with the cardiac index of left ventricular ejection fraction (LVEF). In chronic obstructive pulmonary disease (COPD), VTP showed a negative correlation with forced expiratory volume in the first second (FEV1). VTP values increased with H. pylori infection and were upregulated in atrophic gastritis caused by H. pylori infection. The genes and pathways whose expression perturbations correlated positively with VTP scores across diseases were mainly involved in the regulation of immune, metabolic, and cellular activities.

**Conclusions:**

VTP is upregulated in the disease versus health status, and its upregulation is associated with disease progression and severity in various diseases. Thus, VTP has potential clinical implications for disease diagnosis and prognosis.

## 1. Introduction

With the recent development of next-generation sequencing (NGS) technologies, a substantial number of multiomics data associated with various diseases have been produced, including cancer, neurological disorders, cardiovascular disease, respiratory disease, digestive system disease, metabolic disease, endocrine disease, kidney and urinary system disorders, and infectious disease. In a previous study [1], we developed an algorithm, termed DEPTH, to quantify the variation of transcriptomic perturbations (VTP) in cancer, namely intratumor heterogeneity. We found that VTP value was significantly higher in cancer than in normal controls. Moreover, VTP values increased with cancer advancement, and its increase were associated with worse clinical outcomes in cancer patients [1]. In this study, we generalized this algorithm to a wide variety of diseases and explored the association between VTP and prognosis-associated clinical features. The disease types we analyzed included neurological disorders, infectious diseases, cardiovascular diseases, respiratory diseases, liver diseases, kidney diseases, digestive diseases, and endocrine diseases. We compared VTP values between the disease state and normal controls and analyzed correlations between VTP and disease progression or severity.

## 2. Methods

### 2.1. Algorithm

The algorithm is described as follows: given a transcriptome dataset, which involves *g* genes and *m* disease samples and *n* normal control samples; the variation of transcriptomic perturbations (VTP) of a disease sample DS is defined as

(1) $$\begin{array}{c} \sqrt{\frac{1}{g-1}\left(\overset{g}{\underset{i=1}{\sum }}{\left(\Delta {\left(G_{i},\text{DS},\text{N}{\text{S}}_{j}\right)}^{2}-\frac{1}{g}\overset{g}{\underset{i=1}{\sum }}\Delta {\left(G_{i},\text{DS},\text{N}{\text{S}}_{j}\right)}^{2}\right)}^{2}\right)}=\sqrt{\frac{1}{g-1}\left(\overset{g}{\underset{i=1}{\sum }}{\left({\left(\text{ex}\left(G_{i},\text{DS}\right)-\frac{1}{n}\overset{n}{\underset{j=1}{\sum }}\text{e}\text{x}\left(G_{i},{\text{NS}}_{j}\right)\right)}^{2}-\frac{1}{g}\overset{g}{\underset{i=1}{\sum }}{\left(\text{ex}\left(G_{i},\text{DS}\right)-\frac{1}{n}\overset{n}{\underset{j=1}{\sum }}\text{e}\text{x}\left(G_{i},{\text{NS}}_{j}\right)\right)}^{2}\right)}^{2}\right),}\Delta \left(G_{i},\text{DS},\text{N}{\text{S}}_{j}\right)=\text{ex}\left(G_{i},\text{DS}\right)-\frac{1}{n}\overset{n}{\underset{j=1}{\sum }}\text{e}\text{x}\left(G_{i},{\text{NS}}_{j}\right), \end{array}$$

where *ex*(*G*_*i*_, DS) indicates the expression value of gene *G*_*i*_ in DS, and ex(*G*_*i*_, NS_*j*_) indicates the expression value of *G*_*i*_ in the normal sample NS_*j*_.

### 2.2. Datasets

We downloaded transcriptome datasets for various diseases from the NCBI Gene Expression Omnibus (GEO) (https://www.ncbi.nlm.nih.gov/geo/) and analyzed these datasets with the algorithm. The datasets were associated with various types of diseases, including neurological disorders (Alzheimer's disease (AD) and schizophrenia (SCZ)), infectious diseases (COVID-19, acquired immune deficiency syndrome (AIDS), hepatitis B virus (HBV) infection, tuberculosis (TB), and malaria), cardiovascular diseases (acute myocardial infarction, dilated cardiomyopathy, idiopathic or ischemic cardiomyopathy, and heart failure), respiratory diseases (chronic obstructive pulmonary disease), liver diseases (chronic hepatitis B and liver cirrhosis), kidney diseases (nephrotic syndrome, uremia, focal segmental glomerulosclerosis and glomerular disease), digestive diseases (inflammatory bowel disease and helicobacter pylori infection), and endocrine diseases (diabetes). A description of these datasets is shown in Table 1.

### 2.3. Data Preprocessing

For RNA-Seq gene expression values, we normalized them by the TPM method. For microarray gene expression values, we used the normalization methods recommended by related platforms. A description of the normalization methods for the datasets analyzed was provided in Supplementary Table S1. All normalized expression values were transformed by log2(*x* + 1) before subsequent analyses.

### 2.4. Statistical Analysis and Visualization

We employed the Mann–Whitney *U* test (one-tailed) to compare VTP values between two classes of samples, and the Kruskal-Wallis test to compare VTP values among more than two classes of samples. We utilized the Spearman method to assess the correlation between VTP values and other variables and reported the correlation coefficients (*ρ*) and *P* values. To correct for *P* values in multiple tests, we utilized the Benjamini and Hochberg method to calculate the false discovery rate (FDR) [2]. All statistical analyses were performed in the R programming environment (version 4.1.2). The R packages “ggplot2”, “ggpubr”, and “ggstatsplot” were used for data visualization.

### 2.5. Identifying Genes and Pathways whose Expression Perturbations Have Significant Positive Correlations with VTP across Diverse Diseases

In each dataset, we identified the genes satisfying that |Δ(*G*_*i*_, DS, NS_*j*_)| significantly and positively correlated with VTP values in all disease samples using a threshold of the Spearman correlation test FDR < 0.1. For each disease with *n* datasets analyzed, we identified the genes which satisfied the prior condition at least *n* − 1 datasets. These genes were defined as the genes having significant positive correlations of expression perturbations with VTP in specific diseases. Among them, the genes identified in common in at least 5 specific diseases were defined as the genes whose expression perturbations had significant positive correlations with VTP across diseases. By inputting the genes associated with VTP across diseases into the GSEA web tool [3], we obtained the KEGG pathways [4] having significant positive correlations of their expression perturbations with VTP across diseases using a threshold of FDR<0.1.

## 3. Results

### 3.1. Neurological Disorder

**Figure 1:**
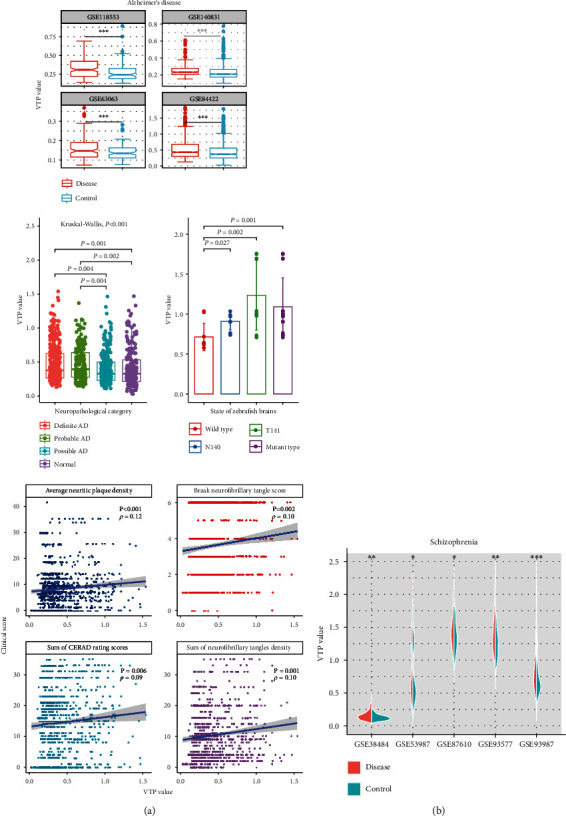
Associations between the VTP measure and disease development and progression in neurological disorder. (a) VTP values are significantly greater in AD patients than in normal controls, larger in define than in possible or probable AD, and increase with AD progression. The measures of Braak neurofibrillary tangle score, average neuritic plaque density, sum of CERAD rating scores in multiple brain regions, and sum of neurofibrillary tangles density in multiple brain regions represent the degree of AD progression. VTP values are remarkedly greater in PSEN2-mutated zebrafish brains than in their wild type controls. (b) VTP values are significantly greater in SCZ patients than in normal controls. AD: Alzheimer's disease. N140: psen2^N140fs^. T141: psen2^T141_L142delinsMISLISV^. SCZ: Schizophrenia. ^∗^*P* < 0.05, ^∗∗^*P* < 0.01, ^∗∗∗^*P* < 0.001. They also apply to the following figures.

AD is a progressive neurodegenerative disease [5]. In four transcriptome datasets for AD (GSE63063 [6], GSE118553 [7], GSE140831, and GSE84422 [8]), the VTP values were significantly larger in AD patients than in normal controls (*P* < 0.001) (Figure 1(a)). In GSE84422, VTP values were significantly larger in define than in possible or probable AD (*P* = 0.02) (Figure 1(a)). In addition, we analyzed correlations between VTP and several measures of the degree of AD progression in GSE84422, including clinical dementia rating, Braak neurofibrillary tangle score, average neuritic plaque density, sum of consortium to establish a registry for Alzheimer's diseases (CERAD) rating scores in multiple brain regions, and sum of neurofibrillary tangles density in multiple brain regions. Notably, VTP displayed significant positive correlations with these measures (*P* < 0.01) (Figure 1(a)). Mutations in PSEN2 may result in early-onset AD. In GSE158233 [9], Barthelson et al. generated transcriptomes of two-types of PSEN2-mutated (psen2^T141_L142delinsMISLISV^ and psen2^N140fs^) lines of zebrafish brains and transcriptomes of their wild type siblings. We observed that VTP values were remarkedly greater in PSEN2-mutated zebrafish brains than in their wild type controls (*P* < 0.03) (Figure 1(a)).

Schizophrenia (SCZ) is a severe psychotic disorder characterized by relapsing incidences of psychosis [10]. In four transcriptome datasets (GSE38484 [11], GSE87610 [12], GSE93577 [13], and GSE93987 [14]) generated from SCZ patients and normal controls, VTP values were consistently greater in SCZ patients than in normal controls (*P* < 0.02) (Figure 1(b)).

Taken together, these results indicate that VTP is augmented in certain neurological disorders (such as AD and SCZ) and grows with disease progression.

### 3.2. Infectious Disease

**Figure 2:**
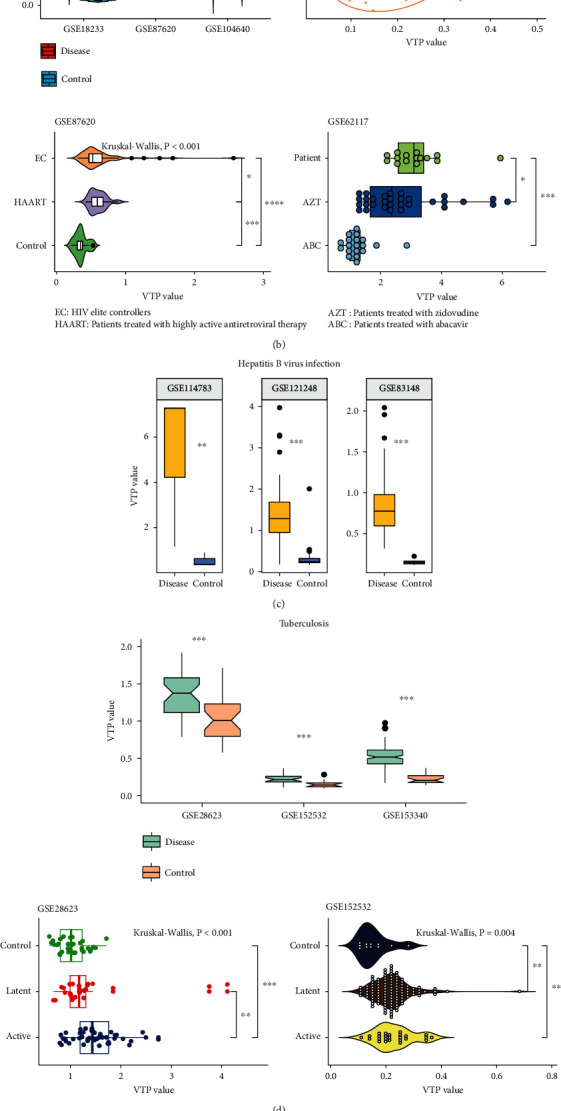
Associations between the VTP measure and disease development and progression in infectious disease. (a) VTP values are significantly greater in COVID-19 patients than in normal controls and increase with disease severity. (b) VTP values are significantly greater in AIDS patients than in normal controls, greater in AIDS patients without treatment than in AIDS patients with treatment, and increase with disease severity. (c). VTP values are significantly greater in HBV-infected patients than in normal controls. (d) VTP values are significantly greater in TB patients than in normal controls and increase with disease progression. (e) VTP values are significantly greater in malaria patients than in normal controls and increase with disease severity. ICU: intensive care unit. MVS: mechanical ventilatory support. SOFA: sequential organ failure assessment. AIDS: acquired immune deficiency syndrome. HBV: hepatitis B virus. TB: tuberculosis.

COVID-19 is a highly contagious disease caused by SARS-CoV-2 infection and is currently widespread around the globe. This disease has caused more than 552 million cases and 6.3 million deaths as of July 1, 2022 [15]. In four transcriptome datasets (GSE152075 [16], GSE157103 [17], GSE161731 [18], and GSE198449 [19]) for COVID-19 patients, VTP values were significantly greater in COVID-19 patients than in normal controls (*P* < 0.01) (Figure 2(a)). Notably, in GSE157103, VTP values were significantly greater in intensive care unit (ICU) COVID-19 patients than in non-ICU patients (*P* < 0.001) (Figure 2(a)). Moreover, COVID-19 patients requiring mechanical ventilatory support (MVS) had greater VTP values than those not requiring MVS (*P* < 0.001) (Figure 2(a)). In addition, VTP displayed a significant positive correlation with the scores of the sequential organ failure assessment (SOFA) (*P* = 0.006; *ρ* = 0.36) (Figure 2(a)), which indicates the severity of ICU patients.

Acquired immune deficiency syndrome (AIDS) is a chronic condition resulting from infection with the human immunodeficiency virus (HIV) [20]. In three transcriptome datasets (GSE18233 [21], GSE87620 [22], and GSE104640 [23]) for AIDS patients, VTP values were significantly upregulated in AIDS patients versus normal controls (*P* < 0.001) (Figure 2(b)). In GSE18233, VTP correlated positively with viral loads (*P* = 0.002; *ρ* = 0.28) (Figure 2(b)). In GSE87620, the AIDS patients with highly active antiretroviral therapy had greater VTP values than elite controllers, who were the AIDS patients with undetectable levels of HIV replication not receiving antiretroviral therapy (*P* = 0.01) (Figure 2(b)). In addition, in GSE62117 [24], the AIDS patients treated with abacavir or zidovudine had lower VTP values than those without such therapies (*P* < 0.05) (Figure 2(b)).

Hepatitis B virus (HBV) infection is a major etiologic factor for hepatocellular carcinoma [25]. In three transcriptome datasets (GSE83148 [26], GSE114783 [27], and GSE121248 [28]), VTP values were greater in HBV-infected patients than in normal controls (*P* < 0.01) (Figure 2(c)).

Pulmonary tuberculosis (TB) is an infectious disease caused by Mycobacterium tuberculosis attacking lungs [29]. In three transcriptome datasets (GSE28623 [30], GSE153340 [31], and GSE152532 [32]), TB patients showed greater VTP values than normal controls (*P* < 0.001) (Figure 2(d)). Moreover, in GSE152532 and GSE28623 [30], VTP values likely followed the pattern: active TB > latent TB > normal controls (Figure 2(d)).

Malaria is a serious disease caused by a parasite and is a major cause of death globally [33]. In three transcriptome datasets (GSE1124 [34], GSE5418 [35], and GSE34404 [36]), malaria patients had greater VTP values than normal controls (*P* < 0.01) (Figure 2(e)). Moreover, in GSE34404, the high parasitemia group had significantly larger VTP values than the low parasitemia group of malaria patients (*P* = 0.004) (Figure 2(e)). In GSE1124, VTP values likely followed the pattern: malaria associated with severe anemia > uncomplicated malaria > asymptomatic infection (Figure 2(e)). In addition, in another transcriptome dataset (GSE5418), VTP values were greater in clinically apparent than in presymptomatic malaria (*P* = 0.006) (Figure 2(e)).

Collectively, these results support that VTP is upregulated in infectious diseases and increases with disease severity.

### 3.3. Cardiovascular Disease

**Figure 3:**
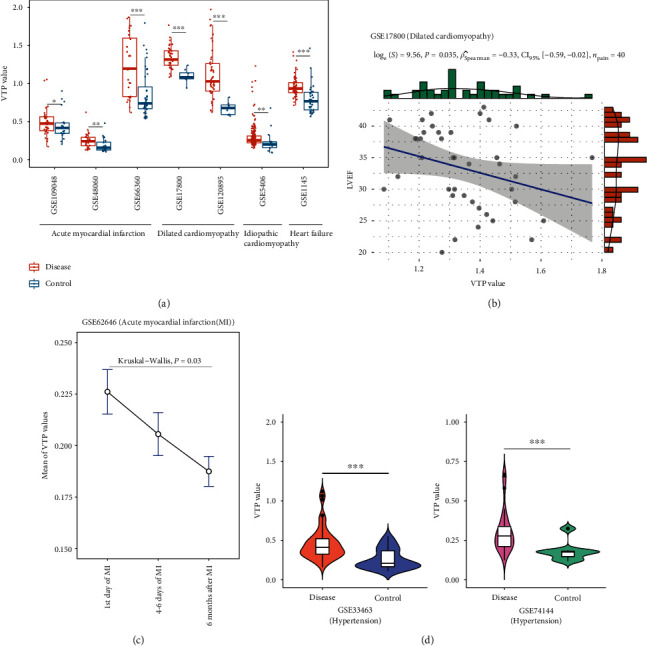
Associations between the VTP measure and disease development and progression in cardiovascular disease. (a) VTP values are significantly greater in heart disease patients than in normal controls and increase with disease severity. (b) VTP values correlate negatively with the cardiac index of LVEF. (c) VTP values decrease with the remission of acute myocardial infarction. (d) VTP values are significantly greater in hypertension patients than in normal controls. LVEF: left ventricular ejection fraction.

Heart disease is the leading cause of death worldwide [37]. In numerous transcriptome datasets of heart disease, such as GSE1145, GSE5406 [38], GSE17800 [39], GSE48060 [40], GSE66360 [41], GSE109048 [42], and GSE120895 [43], VTP values were significantly greater in patients than in normal controls (*P* < 0.05) (Figure 3(a)). In GSE17800, VTP had a significant negative correlation with the cardiac index of left ventricular ejection fraction (LVEF) (*P* = 0.035; *ρ* = −0.33) (Figure 3(b)). In GSE62646 [44], the VTP values calculated based on gene expression patterns in leukocytes from acute myocardial infarction patients followed the pattern: the 1st day of myocardial infarction > after 4 − 6 days > after 6 months (Figure 3(c)). In addition, in two transcriptome datasets (GSE33463 [45] and GSE74144) for hypertension, VTP values were significantly larger in patients than in normal controls (*P* < 0.001) (Figure 3(d)). Altogether, these results suggest that VTP is upregulated in cardiovascular diseases and decreases with disease remission.

### 3.4. Respiratory Disease

**Figure 4:**
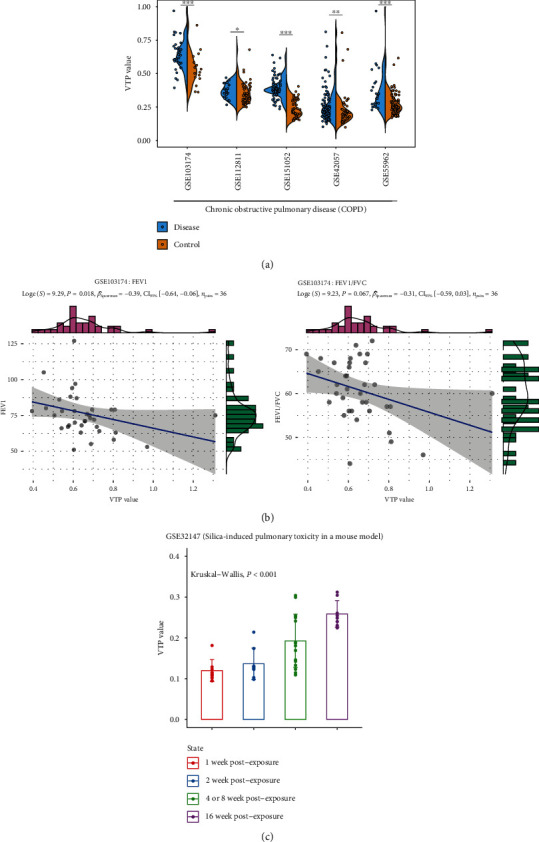
Associations between the VTP measure and disease development and progression in respiratory disease. (a) VTP values are significantly greater in respiratory disease patients than in normal controls. (b) VTP values correlate negatively with FEV1 and ratios of FEV1/FVC in COPD. (c) VTP values increase steadily with the progression of silica-induced pulmonary toxicity in a mouse model. COPD: chronic obstructive pulmonary disease. FEV1: forced expiratory volume in the first second. FVC: forced vital capacity.

Respiratory diseases are the diseases affecting the organs and tissues involved in gas exchange in air-breathing animals [46]. Some of the most common respiratory diseases include obstructive lung disease, restrictive lung disease, and respiratory tract infections. In many transcriptome datasets of respiratory diseases, such as GSE112811, GSE42057 [47], GSE55962 [48], GSE103174, and GSE151052, VTP values were significantly larger in patients than in normal controls (*P* < 0.05) (Figure 4(a)). In chronic obstructive pulmonary disease (COPD), forced expiratory volume in the first second (FEV1) and ratio of FEV1 to forced vital capacity (FVC) are crucial in evaluating the severity of disease [49]. In GSE103174, which is a transcriptome dataset for COPD, VTP showed negative correlations with both FEV1 (*P* = 0.018; *ρ* = −0.39) and FEV1/FVC (*P* = 0.067; *ρ* = −0.31) (Figure 4(b)). The transcriptome dataset GSE32147 [50] is gene expression profiles in lung samples of rats exposed to crystalline silica. We observed that VTP values increased steadily with the progression of silica-induced pulmonary toxicity: 1 week of exposed to crystalline silica < 2 weeks < 4 or 8 weeks < 16 weeks (Figure 4(c)).

Collectively, these results support that VTP is upregulated in respiratory diseases and is negatively associated with their clinical outcomes.

### 3.5. Liver Disease

**Figure 5:**
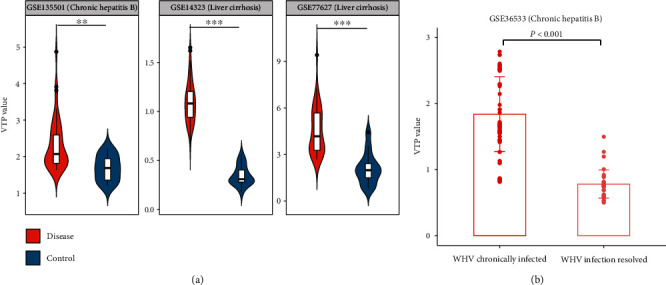
Associations between the VTP measure and disease development and progression in liver disease. (a) VTP values are significantly greater in liver disease patients than in normal controls. (b) VTP values are greater in WHV chronically infected than in infection resolved woodchuck in an animal model. WHV: woodchuck hepatitis virus.

In three transcriptome datasets (GSE14323 [51], GSE77627, and GSE135501) for liver diseases, VTP values were significantly larger in patients than in normal controls (*P* < 0.01) (Figure 5(a)). The transcriptome dataset GSE36533 [52] is gene expression profiles in woodchuck infected with woodchuck hepatitis virus (WHV), an animal model for studying the human HBV. Notably, VTP values are greater in WHV chronically infected than in infection resolved woodchuck (*P* < 0.001) (Figure 5(b)).

### 3.6. Kidney Disease

**Figure 6:**
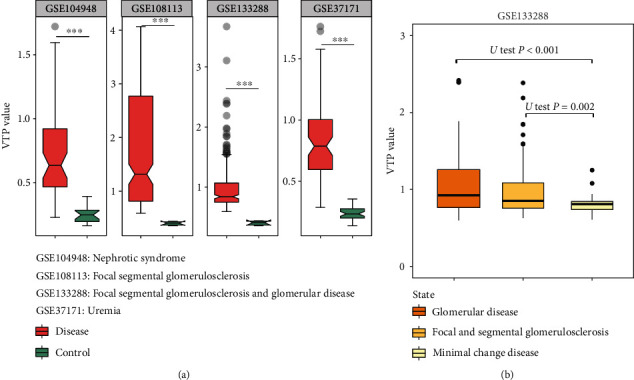
Associations between the VTP measure and disease development and progression in kidney disease. (a) VTP values are significantly greater in kidney disease patients than in normal controls. (b) VTP values correlate positively with disease severity in kidney disease.

In four transcriptome datasets (GSE37171 [53], GSE104948 [54], GSE108113 [54], and GSE133288 [55]) for kidney disease, VTP values were significantly greater in patients than in normal controls (*P* < 0.001) (Figure 6(a)). In addition, in GSE133228, VTP values were significantly larger in focal segmental glomerulosclerosis and glomerular disease than in minimal change disease (*P* < 0.01) (Figure 6(b)). It indicates that VTP values increase with disease progression in kidney disease.

### 3.7. Digestive Disease

**Figure 7:**
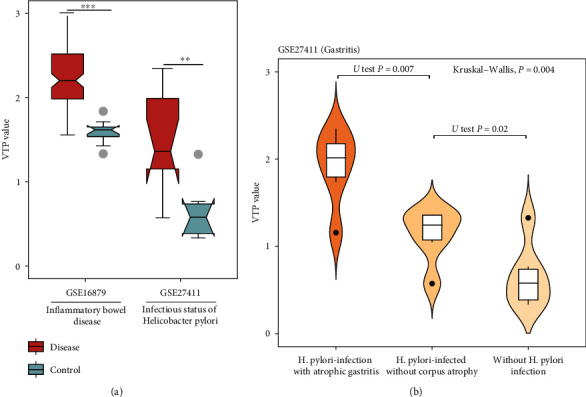
Associations between the VTP measure and disease development and progression in digestive disease. (a) VTP values are significantly greater in digestive disease patients than in normal controls. (b) VTP values correlate positively with disease severity in atrophic gastritis.

In two transcriptome datasets (GSE16879 [56] and GSE27411 [57]) for digestive disease, VTP values were significantly larger in patients than in normal controls (*P* < 0.01) (Figure 7(a)). GSE27411 is a transcriptome dataset for patients with different stages of Helicobacter pylori (H. pylori) infection. Interestingly, we found that VTP values were significantly different among different stages of H. pylori infection and followed the pattern: without current H.pylori infection < H.pylori − infected without corpus atrophy < with current or past H.pylori − infection with corpus-predominant atrophic gastritis (Figure 7(b)). These results collectively support that VTP is upregulated in digestive diseases and increases with disease severity.

### 3.8. Endocrine Disease

**Figure 8:**
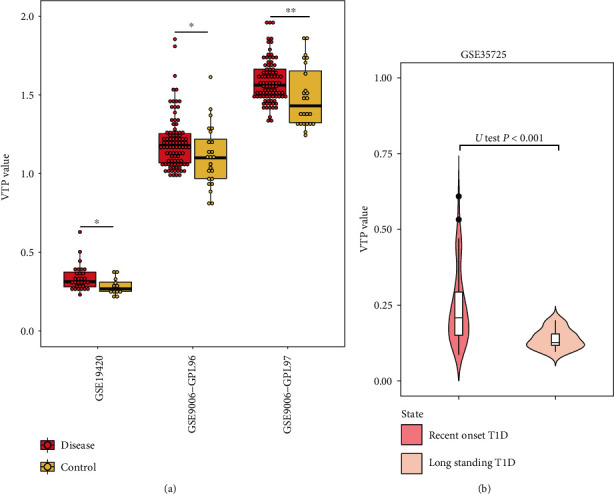
Associations between the VTP measure and disease development and progression in endocrine disease. (a) VTP values are significantly greater in diabetes patients than in normal controls. (b) VTP values are significantly greater in recent onset diabetes patients than in longstanding diabetes patients. T1D: Type 1 diabetes.

Diabetes is a metabolic disease that causes high blood sugar to cause many chronic health problems, such as cardiovascular diseases, vision damage, and kidney disease [58]. In two transcriptome datasets (GSE9006 [59] and GSE19420 [60]) for diabetes, VTP values were significantly greater in patients than in normal controls (*P* < 0.05) (Figure 8(a)). Moreover, in the transcriptome dataset GSE35725 [61] for diabetes, VTP values were significantly greater in recent onset diabetes patients than in longstanding diabetes patients (*P* < 0.001) (Figure 8(b)).

### 3.9. Genes and Pathways whose Expression Perturbations Correlate Positively with VTP across Diseases

**Figure 9:**
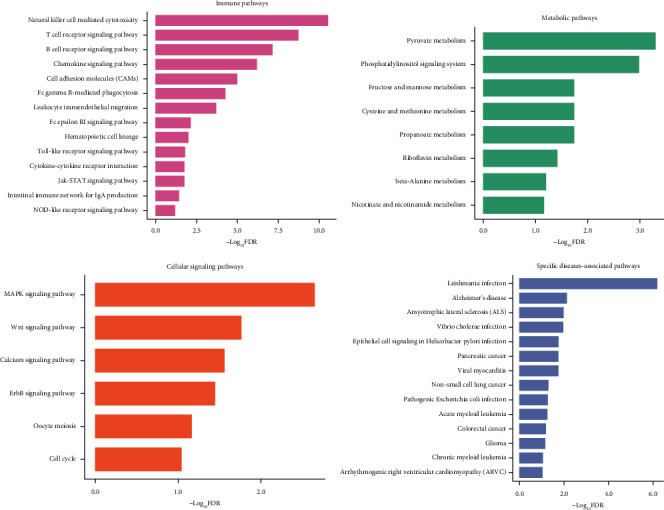
Pathways whose expression perturbations correlate positively with VTP scores across diseases.

We identified 369 genes whose expression perturbations showed significant positive correlations with VTP values across diseases (Supplementary Table S2). Notably, many of these genes are involved in immune regulation (such as CD2, CD247, CD300A, CD2AP, CD28, CD47, CD53, CD7, and CXCR2), cell cycle (such as CCND2, CDK4, and SKP2), and metabolism (such as LDHA, LDHB, PDHA1, GLO1, and ME2). Furthermore, we identified 58 KEGG pathways showing significant positive correlations of expression perturbations with VTP across diseases. Notably, many of these pathways are immune pathways, including natural killer cell-mediated cytotoxicity, T cell receptor signaling, B cell receptor signaling, chemokine signaling, cell adhesion molecules, Fc gamma R-mediated phagocytosis, leukocyte transendothelial migration, Fc epsilon RI signaling, hematopoietic cell lineage, Toll-like receptor signaling, Jak-STAT signaling, cytokine-cytokine receptor interaction, intestinal immune network for IgA production, and NOD-like receptor signaling (Figure 9). The 58 pathways also included many metabolism-related pathways, such as pyruvate metabolism, inositol phosphate metabolism, propanoate metabolism, cysteine and methionine metabolism, fructose and mannose metabolism, riboflavin metabolism, *β*-alanine metabolism, and nicotinate and nicotinamide metabolism. Moreover, many pathways regulating cell growth and division were included in the list of the 58 pathways. Such pathways included MAPK signaling, Wnt signaling, calcium signaling, ErbB signaling, oocyte meiosis, and cell cycle. In addition, the 58 pathways also included many specific diseases-associated pathways, such as leishmania infection, AD, vibrio cholerae infection, epithelial cell signaling in Helicobacter pylori infection, amyotrophic lateral sclerosis, viral myocarditis, pathogenic Escherichia coli infection, arrhythmogenic right ventricular cardiomyopathy, pancreatic cancer, non-small-cell lung cancer, acute myeloid leukemia, colorectal cancer, glioma, and chronic myeloid leukemia.

## 4. Discussion

Although transcriptomic data have been widely applied to biomedical science, few studies have explored the association between transcriptomic perturbations and disease development and progression in a wide variety of diseases. For the first time, we investigated the association between the VTP and various diseases' onset and progression. Our analysis suggests that VTP values are upregulated in various diseases relative to their normal controls, and that VTP values increase with disease progression. Thus, this analysis uncovers a common characteristic of transcriptomic perturbations across various human diseases. In fact, the VTP measure reflects the asynchronous degree of transcriptomic perturbations in a disease status relative to the health status. Our results indicate that the asynchronous degree of transcriptomic perturbations is positively associated with disease progression or severity. That is, the higher asynchronous degree of transcriptomic perturbations suggests more unfavorable clinical outcomes in disease. This is consistent with the findings in cancer [1]. An intriguing question is whether the variation of perturbations in other molecules, such as genome, proteome, and metabolome, has similar associations with disease development and progression.

We identified numerous genes and pathways whose expression perturbations correlated positively with VTP scores across diseases. These genes and pathways are mainly involved in the regulation of immune, metabolic, and cellular activities. It is justified since deregulated immune, metabolic, and cellular activities have been associated with various diseases. Our data suggest that the disordered perturbations of the molecules modulating immune, metabolic, or cellular activities are associated with the development and progression of various diseases. Interestingly, by searching for the database of publicly available GWAS summary statistics (https://www.ebi.ac.uk/gwas/), we found that many of the 369 genes, which displayed significant expression perturbations' correlations with VTP values across diseases, had genetic variants that are statistically associated with the risk of the diseases we analyzed (Supplementary Table S3). For example, there were 16 genes, including RDX, PIP4K2A, PILRA, LPXN, LILRB2, ITGAX, IQGAP2, FOXN2, CR1, CELF2, CDC42SE2, CD2AP, PDK4, PARP8, HSPA6, and BNIP3, whose genetic variants are statistically associated with the risk of AD. Six genes (TKT, TCF4, SWAP70, DDHD2, ARHGAP31, and LTB) showed significant associations of genetic variants with the risk of cardiovascular disease. Notably, FOXN2 had genetic variants statistically associated with the risk of both AD and SCZ, and NOTCH2 displayed genetic variants that are statistically associated with the risk of both endocrine disease and kidney disease. These data support the relevance of many of these genes with the diseases.

This study has several limitations. First, although we have analyzed numerous datasets for various diseases, more datasets are needed to be analyzed to bolster the validity of this analysis. Second, the mechanism underlying the association between VTP and disease development and progression needs to be explored. Finally, the prospect of translating the present findings into clinical practice remains unclear. Nevertheless, our further study is to implement further investigations to overcome these limitations.

## 5. Conclusions

VTP is upregulated in the disease relative to health status, and its upregulation is associated with disease progression and severity in various diseases. The molecules whose abundance perturbations correlate positively with VTP are mainly involved in the regulation of immune, metabolic, and cellular activities. Thus, VTP has potential clinical values in disease diagnosis and prognosis.

## Figures and Tables

**Table 1:** Summary of the datasets analyzed.

Disease	Dataset	Platform	Sample size	
			Patients	Controls
Alzheimer's disease	GSE63063	GPL6947	145	104
		GPL10558	139	134
	GSE84422	GPL96	737	214
			328 (definite AD)	
			180 (probable AD)	
			229 (possible AD)	
		GPL97	737	214
			328 (definite AD)	
			180 (probable AD)	
			229 (possible AD)	
		GPL570	74	28
			34 (definite AD)	
			23 (probable AD)	
			17 (possible AD)	
	GSE118553	GPL10558	167	100
	GSE140831	GPL15988	599	530
	GSE158233	GPL20828	20	10
	Danio rerio dataset			

Schizophrenia	GSE38484	GPL6947	106	96
	GSE53987	GPL570	48	55
	GSE87610	GPL13667	65	72
	GSE93577	GPL13667	70	71
	GSE93987	GPL13158	102	106

Corona virus Disease 2019	GSE152075	GPL18573	430	54
	GSE157103	GPL24676	102	26
			50 (ICU)	
			42 (MVS)	
	GSE161731	GPL24676	46	19
	GSE198449	GPL24676	149	22
			85 (asymptomatic)	
			64 (symptomatic)	

Acquired immune deficiency syndrome	GSE18233	GPL6884	153	3
			16 (EC)	
	GSE87620	GPL10558	83	10
			51 (EC)	
			32 (HAART-treated)	
	GSE104640	GPL10558	188	60

Hepatitis B virus infection	GSE83148	GPL570	122	6
	GSE114783	GPL15491	3	3
	GSE121248	GPL570	70	37

Tuberculosis	GSE28623	GPL4133	71	37
			46 (TB)	
			25 (LTB)	
	GSE153340	GPL21185	18	4
	GSE152532	GPL10558	50	11
			42 (TB)	
			8 (LTB)	

Malaria	GSE1124	GPL96	20	5
			5 (asymptomatic)	
			5 (uncomplicated)	
			5 (severe)	
		GPL97	20	5
			5 (asymptomatic)	
			5 (uncomplicated)	
			5 (severe)	
	GSE5418	GPL96	22	22
	GSE34404	GPL10558	94	61

Cardiovascular disease	GSE1145	GPL570	53	37
	GSE5406	GPL96	194	16
	GSE17800	GPL570	40	8
	GSE33463	GPL6947	49	41
	GSE48060	GPL570	31	21
	GSE62646	GPL6244	28	14
	GSE66360	GPL570	49	50
	GSE74144	GPL13497	28	8
	GSE109048	GPL17586	38	19
	GSE120895	GPL570	47	8

Respiratory disease	GSE5058	GPL570	15	24
	GSE42057	GPL570	94	42
	GSE55962	GPL13667	24	82
	GSE103174	GPL13667	37	16
	GSE112811	GPL570	20	18
	GSE151052	GPL17556	77	40
	GSE32147	GPL6101	56	4
	Rattus norvegicus dataset			

Liver disease	GSE14323	GPL96	58	19
	GSE77627	GPL14951	40	14
			32 (cirrhosis)	
			18 (noncirrhosis)	
	GSE135501	GPL13667	40	14
			16 (white tongue)	
			24 (yellow tongue)	
	GSE36533	GPL15354	11	10
	Marmota monax dataset			

Kidney disease	GSE37171	GPL570	63	20
	GSE104948	GPL22945	53	18
	GSE108113	GPL19983	269	5
	GSE133288	GPL19983	239	5

Digestive disease	GSE16879	GPL570	61	12
	GSE27411	GPL6255	12	6

Endocrine disease	GSE9006	GPL96	55	24
			T1D (43)	
			T2D (12)	
		GPL97	55	24
			T1D (43)	
			T2D (12)	
	GSE19420	GPL570	30	12
	GSE35725	GPL570	57	44
			46 (recent onset)	
			11 (longstanding)	

Note: EC: Elite controllers. HAART: Highly active antiretroviral therapy. LTB: Latently tuberculosis. T1D: Type 1 diabetes. T2D: Type 2 diabetes.

## Data Availability

All data associated with this study are available within the paper and the database of NCBI Gene Expression Omnibus (GEO) (https://www.ncbi.nlm.nih.gov/geo/).

## Supplementary Materials

Supplementary 1 Table S1: a description of the normalization methods for the datasets analyzed.

Supplementary 2 Table S2: 369 genes having significant positive correlations of expression perturbations with VTP values across diseases.

Supplementary 3 Table S3: the genes displayed significant expression perturbations' correlations with VTP values across diseases and had genetic variants statistically associated with a risk of the diseases.
