# Supplementary material for: The Variation of Transcriptomic Perturbations is Associated with the Development and Progression of Various Diseases
Source: Dis Markers. 2022 Sep 26;2022:2148627. doi: 10.1155/2022/2148627 (PMC9530920; doi:10.1155/2022/2148627)
Supplement: Supplementary 1 — Table S1: a description of the normalization methods for the datasets analyzed. [file 2148627.f1.docx]

| **Disease** | **Dataset** | **Platform** | **Type** | **Method** |
| --- | --- | --- | --- | --- |
| Alzheimer's disease | GSE63063 | GPL6947 | Illumina HumanHT-12 V3.0 expression beadchip | The raw data were produced using the Illumina Human HT-12 V3 Bead chip (Array-express: E-TABM-1140) and log-2 transformed signals were normalized using quantile normalization. |
|  |  | GPL10558 | Illumina HumanHT-12 V4.0 expression beadchip |  |
|  | GSE84422 | GPL96 | [HG-U133A] Affymetrix Human Genome U133A Array | The raw microarray data were first quantile normalized with all probesets on the arrays by making use of the RMA method implemented in the R/Bioconductor package affy (v1.44) with the default parameters and then corrected for covariates including sex, postmortem interval (PMI), pH, and race using a linear regression model. |
|  |  | GPL97 | [HG-U133B] Affymetrix Human Genome U133B Array |  |
|  |  | GPL570 | [HG-U133_Plus_2] Affymetrix Human Genome U133 Plus 2.0 Array |  |
|  | GSE118553 | GPL10558 | Illumina HumanHT-12 V4.0 expression beadchip | Using R (version 3.2.2), raw data was Maximum Likelihood Estimation (MLE) background corrected using R package “MBCB” (version 1.18.0), log2 transformed, and underwent Robust Spline Normalisation (RSN) using R package “lumi” (version 2.16.0). |
|  | GSE140831 | GPL15988 | HumanHT-12 v4 Expression BeadChip (nuID) | Gene expression was quantified using Illumina HT12 v4 microarrays. We only considered subjects with diagnoses of control, AD, MCI, bvFTD, svPPA, nfvPPA, PSP or CBS, and also removed RNA samples with RNA integrity number < 6.0. |
|  | GSE158233 | GPL20828 | Illumina NextSeq 500 (Danio rerio) | Pre-processing was performed using the RMA method as implemented in the oligo package. We omitted any probesets that contained a median log2 intensity value of <3.5 (lowly expressed) and also any probesets assigned to multiple genes. |
| Schizophrenia | GSE38484 | GPL6947 | Illumina HumanHT-12 V3.0 expression beadchip | BeadStudio© software version 3.2.3 was used to extract raw data and generate background-corrected gene expression data. Further pre-processing was done using the Lumi package for R. A variance stabilizing transformation (VST) and robust spline normalization (RSN) were applied to the data according to the Lumi procedure. Genes were then filtered based on detection values generated by BeadStudio©. Expression probes had to reach the detection p-value threshold <0.01 in at least one sample. |
|  | GSE53987 | GPL570 | [HG-U133_Plus_2] Affymetrix Human Genome U133 Plus 2.0 Array | Microarray data were normalized by Robust Multi-array Average (RMA). Only genes expressed in all three regions were included in the statistical analysis (N = 22,461). All data (microarray, RT-PCR and cytokine) were analyzed for differential expression using a mixed model ANCOVA using log10 transformed RMA, RQ, or interpolated cytokine protein values as response variable, brain region, and disease group as fixed factors, tetrad as a random factor, and age, sex, tobacco use at time of death, manner of death (accidental, natural, or suicide), PMI, and pH as covariates. |
|  | GSE87610 | GPL13667 | [HG-U219] Affymetrix Human Genome U219 Array | For each of the 74 unique subject samples in each layer, expression intensities were extracted from Affymetrix Expression Console and normalized using Robust Multi-array Average Express. The correlations between replicates were high (0.80<r<0.95), and thus the expression values of the replicate samples from each layer were averaged for data analysis. |
|  | GSE93577 | GPL13667 | [HG-U219] Affymetrix Human Genome U219 Array | For each sample, RNA was extracted using the QIAGEN Micro RNeasy kit Plus (QIAGEN, Valencia, CA, United States). The Ovation Pico WTA System (San Carlos, CA, United States) was used for synthesis and amplification of cDNA and samples were profiled using the Affymetrix GeneChipU219. Data from all 14 samples were normalized together. |
|  | GSE93987 | GPL13158 | [HT_HG-U133_Plus_PM] Affymetrix HT HG-U133+ PM Array Plate | Transcripts selected for validation had a differential expression between subject groups >20% and a high level of expression (Robust Multi-array Average (RMA) normalized value >7). Primer sets were designed outside of the Affymetrix target except when precluded by the small size of some transcripts. |
| Corona Virus Disease 2019 | GSE152075 | GPL18573 | Illumina NextSeq 500 (Homo sapiens)(counts) | Pseudoaligned reads were pre-filtered to remove any genes with average expression of less than one read per sample, then normalized, and DE was calculated with the R package DEseq2 version 1.28.1. Correction for batch effects was incorporated into the design formula and modeling performed using the Wald test with outlier replacement. Results were deemed significant at a Benjamini-Hochberg adjusted p < 0.1. Gene expression differences attributable to sex or age were incorporated into the design formula as interaction terms. |
|  | GSE157103 | GPL24676 | Illumina NovaSeq 6000 (Homo sapiens)(TPM) | Fastq files were trimmed and filtered using a custom algorithm tailored to improve quality scores and maximize retained reads in paired-end data. RNA-Seq expression estimation was performed by RSEM v 1.3.0 (parameters: seed-length=20, no-qualities, bowtie2-k=200, bowtie2-sensitivity-level=sensitive), with bowtie-2 (v 2.3.4.1) for the alignment step, using the custom hg38 reference described above. After the collation of expression estimates, hemoglobin transcripts were removed from further analysis, and TPM values were rescaled to total 1,000,000 in each sample. Differential Expression analysis was performed using the EBSeq package (v 1.26.0) in R (v 3.6.2). |
|  | GSE161731 | GPL24676 | Illumina NovaSeq 6000 (Homo sapiens)(TPM) | RNA Sequencing data was normalized using the frozen RMA method. The sequencing reads were trimmed and aligned to the human reference genome GRCh38 and a count matrix obtained utilizing STAR v2.7.1a. Genes with counts per million greater than 1 in fewer than 20% of samples were dropped along with three samples with a high proportion of lowly expressed reads. The data was normalized using trimmed mean normalization and then log2 transformed. |
|  | GSE198449 | GPL24676 | Illumina NovaSeq 6000 (Homo sapiens)(counts) | All RNA-seq data was processed in a uniform pipeline. Gene expression levels were quantified with kallisto (v0.46.0), using Gencode v34 transcript annotations. Transcript-level quantifications were aggregated to gene level using the tximport (v1.14.2) package, and expression levels were normalized across samples using DESeq2. Differential gene expression analysis was performed with DESeq2, comparing samples during the various time points during infection to baseline gene expression levels, controlling for sex and plate number to minimize batch effects. Immune cell type proportions were estimated from bulk RNA-seq using CIBERSORTx. |
| Acquired immune deficiency syndrome | GSE18233 | GPL6884 | Illumina HumanWG-6 v3.0 expression beadchip | Bead summary data was output from Illumina's BeadStudio software without background correction, as this has previously been shown to have detrimental effects. Data pre-processing, including a variance-stabilizing transformation and robust-spline normalization were applied as implemented in the lumi package of R. Four outlier samples identified based on aberrant expression of control probes and aberrant median-interquartile range values compared to other samples were removed. |
|  | GSE87620 | GPL10558 | Illumina HumanHT-12 V4.0 expression beadchip | Following mRNA extraction from the sorted cells (mirVana™ miRNA Isolation Kit, Ambion), whole-genome transcriptional profiling was performed using Illumina HumanHT-12 V4 microarrays according to standard protocols. Data retrieved from the Illumina software were background corrected (Illumina beadstudio software) and quantile normalized using the Arraystar normalization function. Subsequent data analysis was restricted to genes with significant expression (p<0.05) in at least 95% of samples. |
|  | GSE104640 | GPL10558 | Illumina HumanHT-12 V4.0 expression beadchip | Raw gene expression data was processed in Illumina BeadStudio software, and the lumi R package was used to log2-transform and quantile normalize the expression profiles to stabilize variance and to normalize inter-sample expression profile distributions, respectively. Probe reannotations provided by the illuminaHumanv3.db R package were used to filter out poor probe hybridization specificity. Probes with significant detection in less than 80% of samples were omitted from further analysis. The data was then batch-corrected for sample chip effects using the ComBat R function from the R package sva (freely available from http://www.bioconductor.org). |
| Hepatitis B virus infection | GSE83148 | GPL570 | [HG-U133_Plus_2] Affymetrix Human Genome U133 Plus 2.0 Array | All data were expressed as the mean ± standard deviation (SD). All statistical analyses were conducted with SPSS software v.16.0. The differences in data measurements among the groups were analysed initially by a homogeneity test of variances, and then, either an unpaired Student's t test or one‐way ANOVA was performed under equivalent conditions. Pearson's correlation analysis was conducted for variables correlation. P < 0.05 was considered to be statistically significant. |
|  | GSE114783 | GPL15491 | NimbleGen Human Gene Expression 12x135K Array | Raw data were extracted as pair files by the NimbleScan software (version 2.5); the data were considered robustly expressed if the signal-to-noise ratio was >2. The NimbleScan software uses a robust multi-array analysis algorithm that offers quantile normalization and background correction. Probe level and gene summary files were produced. |
|  | GSE121248 | GPL570 | [HG-U133_Plus_2] Affymetrix Human Genome U133 Plus 2.0 Array | The signal intensity of all the arrays studied was normalized to 500. Only genes that had at least 70% present calls in all the samples studied were grouped under a particular clinicopathologic factor and were kept for further analyses. All data generated by Affymetrix Microarray Suite version 5.0 in cel file format were refined using Genedata Expressionist Refiner software package (Genedata GmbH). |
| Tuberculosis | GSE28623 | GPL4133 | Agilent-014850 Whole Human Genome Microarray 4x44K G4112F (Feature Number version) | Analysis of the scanned images was performed with Feature Extraction software (version 6.1.1, Agilent Technologies). Data analysis was performed using the R package. Data were log-transformed and differentially expressed genes were identified based on log2 fold changes (M-values) in average gene expression with a q value <0.01 (q equals the p value corrected for multiple testing). |
|  | GSE153340 | GPL21185 | Agilent-072363 SurePrint G3 Human GE v3 8x60K Microarray 039494 [Probe Name Version] | Expression data were analysed using ‘R’ Language and Environment for Statistical Computing 3.5.2. Pre-processing, log-2 transformation and normalisation were performed using the Agilp package. Microarrays were run using two batches of microarray slides and Principal Component Analysis identified an associated batch effect. Batch correction was performed using the COmBat function in the Surrogate Variable Analysis (sva) package in R. |
|  | GSE152532 | GPL10558 | Illumina HumanHT-12 V4.0 expression beadchip | The idat Illumina HumanHT12-V4 microarray files were converted to raw expression data using the Bioconductor package beadarray v2.38 in R. The raw data was quantile normalized and log2 transformed using the Bioconductor package lumi v2.40 in R. A comprehensive annotation was performed using annotations provided by biomaRt v2.40.5, the Gene Expression Omnibus (GEO), and platform specific annotation obtained from Illumina. |
| Malaria | GSE1124 | GPL96 | [HG-U133A] Affymetrix Human Genome U133A Array | Signals on the arrays, signal ratios among them and the corresponding measure of likelihood of change and direction (“change P-value”) were calculated with the Affymetrix® Microarray Suite version 5.0 (Affymetrix, Santa Clara, CA, USA). Probe sets whose signal intensities reached values under 50 in all the investigated groups were excluded. The expression of a certain gene was considered increased or decreased if it varied positively by a facto ≥ 1.9 or negatively by a factor ≤ −1.9, and the corresponding change in p value was <0.15 or > 0.85, respectively, in at least 15 comparisons. |
|  |  | GPL97 | [HG-U133B] Affymetrix Human Genome U133B Array |  |
|  | GSE5418 | GPL96 | [HG-U133A] Affymetrix Human Genome U133A Array | Gene expression profiles from both groups of subjects were harvested from Affymetrix U133A GeneChips. The scanned images were analyzed using Affymetrix MAS 5.0 to generate CEL files (fluorescence intensity files), which were normalized at the probe level using the robust multichip average method, with the average fluorescence intensity of each probe expressed as log2. The data sets from all groups (22 data sets from experimentally infected U.S. volunteers, 22 data sets from healthy U.S. volunteers, and 15 data sets from naturally infected Cameroonian volunteers) were normalized together in order to permit direct comparisons of gene expression patterns in the two groups relative to the same baseline. |
|  | GSE34404 | GPL10558 | Illumina HumanHT-12 V4.0 expression beadchip | Genome-wide genotyping data were generated by using OmniExpress arrays (733k SNPs) and extracted with the Genotyping Module in BeadStudio software (Illumina). Only samples with call rates >99% were retained, and all SNPs that had a cluster separation value below 0.3 or call frequency below 99% were removed. |
| Cardiovascular disease | GSE1145 | GPL570 | [HG-U133_Plus_2] Affymetrix Human Genome U133 Plus 2.0 Array |  |
|  | GSE5406 | GPL96 | [HG-U133A] Affymetrix Human Genome U133A Array | Analyses were performed in R software version 1.9-2.2 (www.R-project.org). All.cel files were normalized with the use of robust multiarray analysis (RMA) in the affy package version 1.6.7 (www.bioconductor.org).8 For both the human and the murine data, probe sets were removed if they displayed expression values <91 U (log2=6.5) on all arrays.9 This filtering yielded sets of cardiac genes present well above background levels in either the human or the murine heart. For the human data, we used significance analysis of microarrays (SAM),10 implemented with the use of the siggenes package in R, to select genes that were differentially expressed in failing hearts compared with nonfailing controls. These analyses were stratified by cause of heart failure, ie, ischemic or idiopathic cardiomyopathy. |
|  | GSE17800 | GPL570 | [HG-U133_Plus_2] Affymetrix Human Genome U133 Plus 2.0 Array | RNA was isolated from frozen EMBs (−80°C) following the manufacturer's instructions for total RNA isolation from fibrous tissues (RNeasy® Micro Kit, Qiagen, Inc., Valencia, CA, USA). After purification and quality assessment, transcriptional profiling of EMBs was performed with GeneChip-Human Genome-HG U133-Plus 2.0 arrays (Affymetrix, Santa Clara, CA, USA) and validated for a subset of genes by quantitative reverse-transcription polymerase chain reaction (qRT-PCR). |
|  | GSE33463 | GPL6947 | Illumina HumanHT-12 V3.0 expression beadchip | A single intensity (expression) value for each Illumina probe on the array was obtained using Illumina BeadStudio software with standard settings and no background correction. The expression values for all the probes for each sample were scaled to have median 256 (28) and then log (base 2) transformed before performing statistical analysis. Analysis for differential expression between pairs of disease groups and between individual disease groups and the group of controls was carried out for each Illumina microarray probe. |
|  | GSE48060 | GPL570 | [HG-U133_Plus_2] Affymetrix Human Genome U133 Plus 2.0 Array | Gene filtering was performed using R genefilter package. Differential analysis was performed using empirical Bayesian method implemented in R limma package with FDR control at 0.05 and fold change of 1.2 from AMI patients and control subjects. Due to the limited number of AMI patients with recurrent events, differential analysis between AMI patients with a recurrent event (events groups) and those that were event free (no events group) at 18 months was conducted to determine all genes with a fold change of 1.2 or greater before FDR control. |
|  | GSE62646 | GPL6244 | [HuGene-1_0-st] Affymetrix Human Gene 1.0 ST Array [transcript (gene) version] | Quality controls were performed using Microarray Suite 5.0 software provided by Affymetrix (www.affymetrix.com) according to the manufacturer’s recommendations. Affymetrix raw gene array data were processed using the Partek Genomics Suite software (Partek Inc., St. Louis, MO, USA). |
|  | GSE66360 | GPL570 | [HG-U133_Plus_2] Affymetrix Human Genome U133 Plus 2.0 Array | Normalized expression values for the microarrays were calculated using RMA normalization. Quality controls were conducted with the affy and affyQCReport R packages. A Gaussian mixture clustering of the principal components of the expression data detected eight outliers (five AMI and three control), which were discarded. To select genes for our predictive model, we first removed probe sets mapping to genes that are up-regulated in inflammatory diseases in order to account for the basic health status differences in our cases and controls in the discovery cohort. Next, differential expression analysis in the discovery set was performed via linear regression using the limma package in R. P-values were calculated using an empirical Bayesian method, which were adjusted using the Bonferroni correction. Probe sets with a fold change less than two-times were removed from further consideration. |
|  | GSE74144 | GPL13497 | Agilent-026652 Whole Human Genome Microarray 4x44K v2 (Probe Name version) |  |
|  | GSE109048 | GPL17586 | [HTA-2_0] Affymetrix Human Transcriptome Array 2.0 [transcript (gene) version] | The expression data obtained from the Affymetrix probe-sets (70,523) were processed using Expression Console software (Affymetrix, www.affymetrix.com) with the default parameters. |
|  | GSE120895 | GPL570 | [HG-U133_Plus_2] Affymetrix Human Genome U133 Plus 2.0 Array | EMBs (three or four) from each patient were pooled and homogenized. RNA was isolated following the manufacturer’s instructions for total RNA isolation from fibrous tissues (RNeasy Micro Kit; Qiagen, Valencia, CA). Transcriptional profiling of EMBs was, after purification and quality assessment, performed with GeneChip-Human Genome-HG U133-Plus 2.0-arrays (Affymetrix, Santa Clara, CA) as described earlier (2). Expression data have been submitted to Gene Expression Omnibus (GSE120895). For probe set extraction and normalization, expression raw data were transferred to Rosetta Resolver 7.2 (Ceiba Solutions, Seattle, WA). For further analyses filtering was based on probe sets having signals above background intensity (P < 0.05) in at least 80% of the samples ending up in 18,621 probe sets as basis for subsequent Spearman correlation analysis. |
| Respiratory disease | GSE5058 | GPL570 | [HG-U133_Plus_2] Affymetrix Human Genome U133 Plus 2.0 Array | Captured images were analyzed using Microarray Suite version 5.0 algorithm (Affymetrix). These data were normalized using GeneSpring version 6.2 software (Agilent Technologies) as follows: (a) per array, by dividing raw data by the 50th percentile of all measurements; and (b) per gene, by dividing the raw data by the median expression level for all the genes across all arrays in a data set. All HG-U133A data and HG-U133 Plus 2.0 large airway data was log transformed before statistical analysis. |
|  | GSE42057 | GPL570 | [HG-U133_Plus_2] Affymetrix Human Genome U133 Plus 2.0 Array | The expression of 54,675 transcripts was measured using Affymetrix Human Genome U133 plus 2.0 Gene Array. Quality control was performed, and data were filtered and normalized (see the online supplement). For each probe set, a linear model was fit for the association between gene expression and lung function while controlling for age, sex, body mass index, parental history of COPD, and two smoking variables (smoking status and pack-years). |
|  | GSE55962 | GPL13667 | [HG-U219] Affymetrix Human Genome U219 Array | RNA was extracted using RNeasy Mini Kit (Qiagen, Valencia, US) following manufacturer instructions. RNA integrity was assessed with an Agilent 2100 Bionalyzer (Agilent Technologies). All samples had a RIN above 8. The 60 RNA samples were hybridized to Affymetrix HG-U219 array plate, which enables the performance of up to 96 arrays at one time, following Affymetrix's protocols. |
|  | GSE103174 | GPL13667 | [HG-U219] Affymetrix Human Genome U219 Array | Fresh lung tissue was preserved in RNAlater® (Life Technologies, US). From 53 individuals, total RNA was isolated with PureLink RNA-MiniKit (Life Technologies, US), quantified by Nanodrop (Thermo Scientific, Germany). RNA samples with integrity numbers (RIN) ≥ 7 (Agilent technologies, Germany), where analyzed with the Affymetrix GeneChip® Human Genome U219 Array Plate at the IDIBAPS genomics platform. Microarray results were RMA normalized and probes in the lowest quartile of variability were removed. |
|  | GSE112811 | GPL570 | [HG-U133_Plus_2] Affymetrix Human Genome U133 Plus 2.0 Array |  |
|  | GSE151052 | GPL17556 | [HuGene-1_0-st] Affymetrix Human Gene 1.0 ST Array [HuGene10stv1_Hs_ENTREZG_17.0.0] | Systematic uniform random (SUR) sampling provided 120 samples (8/lung) from 10 explanted lungs of patients with very-severe (Global Initiative for Obstructive Lung Disease, GOLD 4) COPD treated by lung transplantation, and 5 unused donor control lungs. After quality control, three samples were removed from the panel. |
|  | GSE32147 | GPL6101 | Illumina ratRef-12 v1.0 expression beadchip | Illumina BeadArray expression data was analyzed in Bioconductor using the ‘lumi’ and ‘limma’ packages. Bioconductor is a project for the analysis and comprehension of genomic data and operates in R, a statistical computing environment. The ‘lumi’ Bioconductor package was specifically developed to process Illumina microarrays and covers data input, quality control, variance stabilization, normalization and gene annotation. Normalized data were then analyzed using the ‘limma’ package in R. In short, limma fits a linear model for each gene, generates group means of expression and calculates P-values and log fold-changes which are converted to standard fold changes. |
| Liver disease | GSE14323 | GPL96 | [HG-U133A] Affymetrix Human Genome U133A Array | To mitigate any effect due to GeneChip type, prior to obtaining probe set expression summaries, the 9 HG-U133A GeneChips and 115 HG-U133A 2.0 GeneChips were independently read into the R programming environment using the affy Bioconductor package. Thereafter, probe level data from the two GeneChip types were merged by probe sequence using matchprobes package in R. Subsequently, the robust multiarray average method was used to obtain probe set expression summaries. |
|  | GSE77627 | GPL14951 | Illumina HumanHT-12 WG-DASL V4.0 R2 expression beadchip |  |
|  | GSE135501 | GPL13667 | [HG-U219] Affymetrix Human Genome U219 Array | The peripheral blood samples were collected by TempusTM Blood RNA Tubes (Thermo Fisher Scientific), Affymetrix Human Genome U219 Array plates were used. The quality control data was normalized by the RMA algorithm using Gene Spring Software 11.5 (Agilent). |
|  | GSE36533 | GPL15354 | NimbleGen Woodchuck Gene Expression HX3 Microarray | The gene expression data was normalized by the robust multichip average algorithm implemented in Partek Genomics Suite 6.5, and ANOVA was used to derive lists of differentially expressed probesets. Multiple testing correction was performed using the method of Benjamini and Hochberg. |
| Kidney disease | GSE37171 | GPL570 | [HG-U133_Plus_2] Affymetrix Human Genome U133 Plus 2.0 Array | Quality of the samples, hybridization, chips and scanning was reviewed using the BioConductor packages Affy version 1.16.0 and affyPLM version 1.14.0. Data import, normalization and statistical analysis were performed using the Partek Genomics Suite, version 6.5 (Partek, St Louis, MI). RMA background correction and quantile normalization were applied followed by log2-transformation. An unsupervised raw expression filter was applied with a threshold of signal intensity of 6 in a number of samples equal to 75% of the smallest sample group. |
|  | GSE104948 | GPL22945 | [HG-U133_Plus_2] Affymetrix Human Genome U133 Plus 2.0 Array [CDF: Brainarray HGU133Plus2_Hs_ENTREZG_v19] | Transcriptional data were used to assess reliability of microdissection, targeting 16-fold to 64-fold enrichment of glomerular-selective or tubulointerstitial-selective transcripts in each respective compartment. In the discovery cohort, RNA from each compartment was processed and analysed using Affymetrix GeneChip Human Genome U133A V.2.0 and U133 Plus V.2.0 platforms. In the validation cohort, samples were profiled on a Human Gene ST 2.1 array platform. Probe sets were annotated to Entrez Gene IDs using custom CDF V.19 generated from the University of Michigan Brain Array group, as previously described. |
|  | GSE108113 | GPL19983 | [HuGene-2_1-st] Affymetrix Human Gene 2.1 ST Array [HuGene21st_Hs_ENTREZG_19.0.0] | Transcriptional data were used to assess reliability of microdissection, targeting 16-fold to 64-fold enrichment of glomerular-selective or tubulointerstitial-selective transcripts in each respective compartment. In the discovery cohort, RNA from each compartment was processed and analysed using Affymetrix GeneChip Human Genome U133A V.2.0 and U133 Plus V.2.0 platforms. In the validation cohort, samples were profiled on a Human Gene ST 2.1 array platform. Probe sets were annotated to Entrez Gene IDs using custom CDF V.19 generated from the University of Michigan Brain Array group, as previously described. |
|  | GSE133288 | GPL19983 | [HuGene-2_1-st] Affymetrix Human Gene 2.1 ST Array [HuGene21st_Hs_ENTREZG_19.0.0] | Gene expression was normalized, quantified and annotated at the Entrez Gene level. Only genes expressed 1 standard deviation (SD) above the negative control were considered to be expressed and included in the analysis. |
| Digestive disease | GSE16879 | GPL570 | [HG-U133_Plus_2] Affymetrix Human Genome U133 Plus 2.0 Array | The Affymetrix raw data (.cel files) were analyzed using Bioconductor tools in R (version 2.7.2, http://www.r-project.org/). The robust multichip average method was performed on the Affymetrix raw data (.cel files) to obtain a log2 expression value for each probe set. |
|  | GSE27411 | GPL6255 | Illumina humanRef-8 v2.0 expression beadchip | All raw signal intensity files from the BeadStudio were processed together by R software equipped 'lumi’ package using quantile normalization. |
| Endocrine disease | GSE9006 | GPL96 | [HG-U133A] Affymetrix Human Genome U133A Array | For each Affymetrix U133A or U133B Gene Chip, raw intensity data were normalized to the mean intensity of all measurements on that chip and scaled to a target intensity value of 500 in GeneChip Operating System version 1.0. With use of Genespring software, version 7.3.1, the value for each gene in each patient sample array was divided by the median of that gene’s measurement from the cohort of healthy volunteers. |
|  |  | GPL97 | [HG-U133B] Affymetrix Human Genome U133B Array |  |
|  | GSE19420 | GPL570 | [HG-U133_Plus_2] Affymetrix Human Genome U133 Plus 2.0 Array | Muscle RNA was amplified and hybridized on Affymetrix (Santa Clara, CA) U133 plus 2.0 arrays. The microarray data reported in this manuscript have been deposited in the NCBI Gene Expression Omnibus (GEO), accession number GSE19420. |
|  | GSE35725 | GPL570 | [HG-U133_Plus_2] Affymetrix Human Genome U133 Plus 2.0 Array | Image data were analyzed with Affymetrix Expression Console™ 1.1.2 software and normalized with Robust Multichip Analysis (www.bioconductor.org) to determine signal log ratios. |
