# Supplementary material for: The Variation of Transcriptomic Perturbations is Associated with the Development and Progression of Various Diseases
Source: Dis Markers. 2022 Sep 26;2022:2148627. doi: 10.1155/2022/2148627 (PMC9530920; doi:10.1155/2022/2148627)
Supplement: Supplementary 2 — Table S2: 369 genes having significant positive correlations of expression perturbations with VTP values across diseases. [file 2148627.f2.docx]

| **ENTREZ Gene ID** | **Gene Name** | **ENTREZ_GENE_SUMMARY** | **KEGG_PATHWAY** |
| --- | --- | --- | --- |
| 55326 | 1-acylglycerol-3-phosphate O-acyltransferase 5(AGPAT5) | This gene encodes a member of the 1-acylglycerol-3-phosphate O-acyltransferase family. This integral membrane protein converts lysophosphatidic acid to phosphatidic acid, the second step in de novo phospholipid biosynthesis. A pseudogene of this gene is present on the Y chromosome. [provided by RefSeq, Aug 2014], | hsa00561:Glycerolipid metabolism,hsa00564:Glycerophospholipid metabolism,hsa01100:Metabolic pathways,hsa04072:Phospholipase D signaling pathway, |
| 18 | 4-aminobutyrate aminotransferase(ABAT) | 4-aminobutyrate aminotransferase (ABAT) is responsible for catabolism of gamma-aminobutyric acid (GABA), an important, mostly inhibitory neurotransmitter in the central nervous system, into succinic semialdehyde. The active enzyme is a homodimer of 50-kD subunits complexed to pyridoxal-5-phosphate. The protein sequence is over 95% similar to the pig protein. GABA is estimated to be present in nearly one-third of human synapses. ABAT in liver and brain is controlled by 2 codominant alleles with a frequency in a Caucasian population of 0.56 and 0.44. The ABAT deficiency phenotype includes psychomotor retardation, hypotonia, hyperreflexia, lethargy, refractory seizures, and EEG abnormalities. Multiple alternatively spliced transcript variants encoding the same protein isoform have been found for this gene. [provided by RefSeq, Jul 2008], | hsa00250:Alanine, aspartate and glutamate metabolism,hsa00280:Valine, leucine and isoleucine degradation,hsa00410:beta-Alanine metabolism,hsa00640:Propanoate metabolism,hsa00650:Butanoate metabolism,hsa01100:Metabolic pathways,hsa04727:GABAergic synapse, |
| 5209 | 6-phosphofructo-2-kinase/fructose-2,6-biphosphatase 3(PFKFB3) | The protein encoded by this gene belongs to a family of bifunctional proteins that are involved in both the synthesis and degradation of fructose-2,6-bisphosphate, a regulatory molecule that controls glycolysis in eukaryotes. The encoded protein has a 6-phosphofructo-2-kinase activity that catalyzes the synthesis of fructose-2,6-bisphosphate (F2,6BP), and a fructose-2,6-biphosphatase activity that catalyzes the degradation of F2,6BP. This protein is required for cell cycle progression and prevention of apoptosis. It functions as a regulator of cyclin-dependent kinase 1, linking glucose metabolism to cell proliferation and survival in tumor cells. Several alternatively spliced transcript variants encoding different isoforms have been found for this gene. [provided by RefSeq, Apr 2016], | hsa00051:Fructose and mannose metabolism,hsa01100:Metabolic pathways,hsa04066:HIF-1 signaling pathway,hsa04152:AMPK signaling pathway, |
| 11215 | A-kinase anchoring protein 11(AKAP11) | The A-kinase anchor proteins (AKAPs) are a group of structurally diverse proteins, which have the common function of binding to the regulatory subunit of protein kinase A (PKA) and confining the holoenzyme to discrete locations within the cell. This gene encodes a member of the AKAP family. The encoded protein is expressed at high levels throughout spermatogenesis and in mature sperm. It binds the RI and RII subunits of PKA in testis. It may serve a function in cell cycle control of both somatic cells and germ cells in addition to its putative role in spermatogenesis and sperm function. [provided by RefSeq, Jul 2008], |  |
| 83440 | ADP dependent glucokinase(ADPGK) | ADPGK (EC 2.7.1.147) catalyzes the ADP-dependent phosphorylation of glucose to glucose-6-phosphate and may play a role in glycolysis, possibly during ischemic conditions (Ronimus and Morgan, 2004 [PubMed 14975750]).[supplied by OMIM, Mar 2008], | hsa00010:Glycolysis / Gluconeogenesis,hsa01100:Metabolic pathways,hsa01200:Carbon metabolism, |
| 10123 | ADP ribosylation factor like GTPase 4C(ARL4C) | ADP-ribosylation factor-like 4C is a member of the ADP-ribosylation factor family of GTP-binding proteins. ARL4C is closely similar to ARL4A and ARL4D and each has a nuclear localization signal and an unusually high guanine nucleotide exchange rate. This protein may play a role in cholesterol transport. [provided by RefSeq, Jul 2008], |  |
| 165 | AE binding protein 1(AEBP1) | This gene encodes a member of carboxypeptidase A protein family. The encoded protein may function as a transcriptional repressor and play a role in adipogenesis and smooth muscle cell differentiation. Studies in mice suggest that this gene functions in wound healing and abdominal wall development. Overexpression of this gene is associated with glioblastoma. [provided by RefSeq, May 2013], |  |
| 79572 | ATPase 13A3(ATP13A3) | ATP13A3 is a member of the P-type ATPase family of proteins that transport a variety of cations across membranes. Other P-type ATPases include ATP7B (MIM 606882) and ATP7A (MIM 300011).[supplied by OMIM, Aug 2008], |  |
| 523 | ATPase H+ transporting V1 subunit A(ATP6V1A) | This gene encodes a component of vacuolar ATPase (V-ATPase), a multisubunit enzyme that mediates acidification of eukaryotic intracellular organelles. V-ATPase dependent organelle acidification is necessary for such intracellular processes as protein sorting, zymogen activation, receptor-mediated endocytosis, and synaptic vesicle proton gradient generation. V-ATPase is composed of a cytosolic V1 domain and a transmembrane V0 domain. The V1 domain consists of three A and three B subunits, two G subunits plus the C, D, E, F, and H subunits. The V1 domain contains the ATP catalytic site. The V0 domain consists of five different subunits: a, c, c&apos;, c&quot;, and d. Additional isoforms of many of the V1 and V0 subunit proteins are encoded by multiple genes or alternatively spliced transcript variants. This encoded protein is one of two V1 domain A subunit isoforms and is found in all tissues. Transcript variants derived from alternative polyadenylation exist. [provided by RefSeq, Jul 2008], | hsa00190:Oxidative phosphorylation,hsa01100:Metabolic pathways,hsa04145:Phagosome,hsa04150:mTOR signaling pathway,hsa04721:Synaptic vesicle cycle,hsa04966:Collecting duct acid secretion,hsa05110:Vibrio cholerae infection,hsa05120:Epithelial cell signaling in Helicobacter pylori infection,hsa05165:Human papillomavirus infection,hsa05323:Rheumatoid arthritis, |
| 528 | ATPase H+ transporting V1 subunit C1(ATP6V1C1) | This gene encodes a component of vacuolar ATPase (V-ATPase), a multisubunit enzyme that mediates acidification of intracellular compartments of eukaryotic cells. V-ATPase dependent acidification is necessary for such intracellular processes as protein sorting, zymogen activation, receptor-mediated endocytosis, and synaptic vesicle proton gradient generation. V-ATPase is composed of a cytosolic V1 domain and a transmembrane V0 domain. The V1 domain consists of three A and three B subunits, two G subunits plus the C, D, E, F, and H subunits. The V1 domain contains the ATP catalytic site. The V0 domain consists of five different subunits: a, c, c&apos;, c&apos;&apos;, and d. Additional isoforms of many of the V1 and V0 subunit proteins are encoded by multiple genes or alternatively spliced transcript variants. This gene is one of two genes that encode the V1 domain C subunit proteins and is found ubiquitously. This C subunit is analogous but not homologous to gamma subunit of F-ATPases. Previously, this gene was designated ATP6D. [provided by RefSeq, Jul 2008], | hsa00190:Oxidative phosphorylation,hsa01100:Metabolic pathways,hsa04145:Phagosome,hsa04150:mTOR signaling pathway,hsa04721:Synaptic vesicle cycle,hsa04966:Collecting duct acid secretion,hsa05110:Vibrio cholerae infection,hsa05120:Epithelial cell signaling in Helicobacter pylori infection,hsa05165:Human papillomavirus infection,hsa05323:Rheumatoid arthritis, |
| 57198 | ATPase phospholipid transporting 8B2(ATP8B2) | The protein encoded by this gene belongs to the family of P-type cation transport ATPases, and to the subfamily of aminophospholipid-transporting ATPases. The aminophospholipid translocases transport phosphatidylserine and phosphatidylethanolamine from one side of a bilayer to another. Alternatively spliced transcript variants encoding different isoforms have been identified. [provided by RefSeq, Jul 2008], |  |
| 116985 | ArfGAP with RhoGAP domain, ankyrin repeat and PH domain 1(ARAP1) | The protein encoded by this gene contains SAM, ARF-GAP, RHO-GAP, ankyrin repeat, RAS-associating, and pleckstrin homology (PH) domains. In vitro, this protein displays RHO-GAP and phosphatidylinositol (3,4,5) trisphosphate (PIP3)-dependent ARF-GAP activity. The encoded protein associates with the Golgi, and the ARF-GAP activity mediates changes in the Golgi and the formation of filopodia. It is thought to regulate the cell-specific trafficking of a receptor protein involved in apoptosis. Multiple transcript variants encoding different isoforms have been found for this gene. [provided by RefSeq, Sep 2008], | hsa04144:Endocytosis, |
| 50807 | ArfGAP with SH3 domain, ankyrin repeat and PH domain 1(ASAP1) | This gene encodes an ADP-ribosylation factor (ARF) GTPase-activating protein. The GTPase-activating activity is stimulated by phosphatidylinositol 4,5-biphosphate (PIP2), and is greater towards ARF1 and ARF5, and lesser for ARF6. This gene maybe involved in regulation of membrane trafficking and cytoskeleton remodeling. Alternatively spliced transcript variants encoding different isoforms have been found for this gene. [provided by RefSeq, Oct 2011], | hsa04144:Endocytosis,hsa04666:Fc gamma R-mediated phagocytosis, |
| 9744 | ArfGAP with coiled-coil, ankyrin repeat and PH domains 1(ACAP1) | Predicted to enable GTPase activator activity and metal ion binding activity. Predicted to be involved in protein transport and regulation of catalytic activity. Located in membrane. [provided by Alliance of Genome Resources, Apr 2022], | hsa04144:Endocytosis, |
| 23527 | ArfGAP with coiled-coil, ankyrin repeat and PH domains 2(ACAP2) | Enables GTPase activator activity. Acts upstream of or within actin filament-based process. Located in ruffle. [provided by Alliance of Genome Resources, Apr 2022], | hsa04144:Endocytosis, |
| 55973 | B cell receptor associated protein 29(BCAP29) | Involved in osteoblast differentiation. Located in membrane. [provided by Alliance of Genome Resources, Apr 2022], |  |
| 664 | BCL2 interacting protein 3(BNIP3) | This gene is encodes a mitochondrial protein that contains a BH3 domain and acts as a pro-apoptotic factor. The encoded protein interacts with anti-apoptotic proteins, including the E1B 19 kDa protein and Bcl2. This gene is silenced in tumors by DNA methylation. [provided by RefSeq, Dec 2014], | hsa04068:FoxO signaling pathway,hsa04137:Mitophagy - animal,hsa04140:Autophagy - animal,hsa05131:Shigellosis,hsa05134:Legionellosis, |
| 597 | BCL2 related protein A1(BCL2A1) | This gene encodes a member of the BCL-2 protein family. The proteins of this family form hetero- or homodimers and act as anti- and pro-apoptotic regulators that are involved in a wide variety of cellular activities such as embryonic development, homeostasis and tumorigenesis. The protein encoded by this gene is able to reduce the release of pro-apoptotic cytochrome c from mitochondria and block caspase activation. This gene is a direct transcription target of NF-kappa B in response to inflammatory mediators, and is up-regulated by different extracellular signals, such as granulocyte-macrophage colony-stimulating factor (GM-CSF), CD40, phorbol ester and inflammatory cytokine TNF and IL-1, which suggests a cytoprotective function that is essential for lymphocyte activation as well as cell survival. Alternatively spliced transcript variants encoding different isoforms have been found for this gene. [provided by RefSeq, Jul 2008], | hsa04064:NF-kappa B signaling pathway,hsa04210:Apoptosis,hsa05202:Transcriptional misregulation in cancer,hsa05221:Acute myeloid leukemia, |
| 604 | BCL6 transcription repressor(BCL6) | The protein encoded by this gene is a zinc finger transcription factor and contains an N-terminal POZ domain. This protein acts as a sequence-specific repressor of transcription, and has been shown to modulate the transcription of STAT-dependent IL-4 responses of B cells. This protein can interact with a variety of POZ-containing proteins that function as transcription corepressors. This gene is found to be frequently translocated and hypermutated in diffuse large-cell lymphoma (DLCL), and may be involved in the pathogenesis of DLCL. Alternatively spliced transcript variants encoding different protein isoforms have been found for this gene. [provided by RefSeq, Aug 2015], | hsa04068:FoxO signaling pathway,hsa05202:Transcriptional misregulation in cancer,hsa05207:Chemical carcinogenesis - receptor activation, |
| 637 | BH3 interacting domain death agonist(BID) | This gene encodes a death agonist that heterodimerizes with either agonist BAX or antagonist BCL2, and thus regulate apoptosis. The encoded protein is a member of the BCL-2 family of cell death regulators. It is a mediator of mitochondrial damage induced by caspase-8 (CASP8); CASP8 cleaves this encoded protein, and the COOH-terminal part translocates to mitochondria where it triggers cytochrome c release. Multiple alternatively spliced transcript variants have been found. [provided by RefSeq, Aug 2020], | hsa01524:Platinum drug resistance,hsa04071:Sphingolipid signaling pathway,hsa04115:p53 signaling pathway,hsa04210:Apoptosis,hsa04215:Apoptosis - multiple species,hsa04217:Necroptosis,hsa04650:Natural killer cell mediated cytotoxicity,hsa04932:Non-alcoholic fatty liver disease,hsa05010:Alzheimer disease,hsa05014:Amyotrophic lateral sclerosis,hsa05022:Pathways of neurodegeneration - multiple diseases,hsa05152:Tuberculosis,hsa05160:Hepatitis C,hsa05161:Hepatitis B,hsa05162:Measles,hsa05163:Human cytomegalovirus infection,hsa05164:Influenza A,hsa05167:Kaposi sarcoma-associated herpesvirus infection,hsa05168:Herpes simplex virus 1 infection,hsa05169:Epstein-Barr virus infection,hsa05170:Human immunodeficiency virus 1 infection,hsa05200:Pathways in cancer,hsa05416:Viral myocarditis,hsa05417:Lipid and atherosclerosis, |
| 6347 | C-C motif chemokine ligand 2(CCL2) | This gene is one of several cytokine genes clustered on the q-arm of chromosome 17. Chemokines are a superfamily of secreted proteins involved in immunoregulatory and inflammatory processes. The superfamily is divided into four subfamilies based on the arrangement of N-terminal cysteine residues of the mature peptide. This chemokine is a member of the CC subfamily which is characterized by two adjacent cysteine residues. This cytokine displays chemotactic activity for monocytes and basophils but not for neutrophils or eosinophils. It has been implicated in the pathogenesis of diseases characterized by monocytic infiltrates, like psoriasis, rheumatoid arthritis and atherosclerosis. It binds to chemokine receptors CCR2 and CCR4. Elevated expression of the encoded protein is associated with severe acute respiratory syndrome coronavirus 2 (SARS&amp;#8208;CoV&amp;#8208;2) infection. [provided by RefSeq, Aug 2020], | hsa04060:Cytokine-cytokine receptor interaction,hsa04061:Viral protein interaction with cytokine and cytokine receptor,hsa04062:Chemokine signaling pathway,hsa04621:NOD-like receptor signaling pathway,hsa04657:IL-17 signaling pathway,hsa04668:TNF signaling pathway,hsa04933:AGE-RAGE signaling pathway in diabetic complications,hsa05135:Yersinia infection,hsa05142:Chagas disease,hsa05144:Malaria,hsa05163:Human cytomegalovirus infection,hsa05164:Influenza A,hsa05168:Herpes simplex virus 1 infection,hsa05171:Coronavirus disease - COVID-19,hsa05323:Rheumatoid arthritis,hsa05417:Lipid and atherosclerosis,hsa05418:Fluid shear stress and atherosclerosis, |
| 1230 | C-C motif chemokine receptor 1(CCR1) | This gene encodes a member of the beta chemokine receptor family, which is predicted to be a seven transmembrane protein similar to G protein-coupled receptors. The ligands of this receptor include macrophage inflammatory protein 1 alpha (MIP-1 alpha), regulated on activation normal T expressed and secreted protein (RANTES), monocyte chemoattractant protein 3 (MCP-3), and myeloid progenitor inhibitory factor-1 (MPIF-1). Chemokines and their receptors mediated signal transduction are critical for the recruitment of effector immune cells to the site of inflammation. Knockout studies of the mouse homolog suggested the roles of this gene in host protection from inflammatory response, and susceptibility to virus and parasite. This gene and other chemokine receptor genes, including CCR2, CCRL2, CCR3, CCR5 and CCXCR1, are found to form a gene cluster on chromosome 3p. [provided by RefSeq, Jul 2008], | hsa04060:Cytokine-cytokine receptor interaction,hsa04061:Viral protein interaction with cytokine and cytokine receptor,hsa04062:Chemokine signaling pathway,hsa05163:Human cytomegalovirus infection,hsa05167:Kaposi sarcoma-associated herpesvirus infection, |
| 3579 | C-X-C motif chemokine receptor 2(CXCR2) | The protein encoded by this gene is a member of the G-protein-coupled receptor family. This protein is a receptor for interleukin 8 (IL8). It binds to IL8 with high affinity, and transduces the signal through a G-protein activated second messenger system. This receptor also binds to chemokine (C-X-C motif) ligand 1 (CXCL1/MGSA), a protein with melanoma growth stimulating activity, and has been shown to be a major component required for serum-dependent melanoma cell growth. This receptor mediates neutrophil migration to sites of inflammation. The angiogenic effects of IL8 in intestinal microvascular endothelial cells are found to be mediated by this receptor. Knockout studies in mice suggested that this receptor controls the positioning of oligodendrocyte precursors in developing spinal cord by arresting their migration. This gene, IL8RA, a gene encoding another high affinity IL8 receptor, as well as IL8RBP, a pseudogene of IL8RB, form a gene cluster in a region mapped to chromosome 2q33-q36. Alternatively spliced variants, encoding the same protein, have been identified. [provided by RefSeq, Nov 2009], | hsa04060:Cytokine-cytokine receptor interaction,hsa04061:Viral protein interaction with cytokine and cytokine receptor,hsa04062:Chemokine signaling pathway,hsa04072:Phospholipase D signaling pathway,hsa04144:Endocytosis,hsa05120:Epithelial cell signaling in Helicobacter pylori infection,hsa05163:Human cytomegalovirus infection, |
| 50856 | C-type lectin domain family 4 member A(CLEC4A) | This gene encodes a member of the C-type lectin/C-type lectin-like domain (CTL/CTLD) superfamily. Members of this family share a common protein fold and have diverse functions, such as cell adhesion, cell-cell signalling, glycoprotein turnover, and roles in inflammation and immune response. The encoded type 2 transmembrane protein may play a role in inflammatory and immune response. Multiple transcript variants encoding distinct isoforms have been identified for this gene. This gene is closely linked to other CTL/CTLD superfamily members on chromosome 12p13 in the natural killer gene complex region. [provided by RefSeq, Jul 2008], |  |
| 25966 | C2 calcium dependent domain containing 2(C2CD2) | Located in cytosol and nucleus. [provided by Alliance of Genome Resources, Apr 2022], |  |
| 81688 | Chromosome 6 open reading frame 62(C6orf62) | No summary. | hsa05100:Bacterial invasion of epithelial cells, |
| 23607 | CD2 associated protein(CD2AP) | This gene encodes a scaffolding molecule that regulates the actin cytoskeleton. The protein directly interacts with filamentous actin and a variety of cell membrane proteins through multiple actin binding sites, SH3 domains, and a proline-rich region containing binding sites for SH3 domains. The cytoplasmic protein localizes to membrane ruffles, lipid rafts, and the leading edges of cells. It is implicated in dynamic actin remodeling and membrane trafficking that occurs during receptor endocytosis and cytokinesis. Haploinsufficiency of this gene is implicated in susceptibility to glomerular disease. [provided by RefSeq, Jul 2008], | hsa05100:Bacterial invasion of epithelial cells, |
| 914 | CD2 molecule(CD2) | The protein encoded by this gene is a surface antigen found on all peripheral blood T-cells. The encoded protein interacts with LFA3 (CD58) on antigen presenting cells to optimize immune recognition. A locus control region (LCR) has been found in the 3&apos; flanking sequence of this gene. [provided by RefSeq, Jun 2016], | hsa04514:Cell adhesion molecules,hsa04640:Hematopoietic cell lineage, |
| 919 | CD247 molecule(CD247) | The protein encoded by this gene is T-cell receptor zeta, which together with T-cell receptor alpha/beta and gamma/delta heterodimers, and with CD3-gamma, -delta and -epsilon, forms the T-cell receptor-CD3 complex. The zeta chain plays an important role in coupling antigen recognition to several intracellular signal-transduction pathways. Low expression of the antigen results in impaired immune response. Two alternatively spliced transcript variants encoding distinct isoforms have been found for this gene. [provided by RefSeq, Jul 2008], | hsa04650:Natural killer cell mediated cytotoxicity,hsa04658:Th1 and Th2 cell differentiation,hsa04659:Th17 cell differentiation,hsa04660:T cell receptor signaling pathway,hsa05142:Chagas disease,hsa05169:Epstein-Barr virus infection,hsa05170:Human immunodeficiency virus 1 infection,hsa05235:PD-L1 expression and PD-1 checkpoint pathway in cancer, |
| 940 | CD28 molecule(CD28) | The protein encoded by this gene is essential for T-cell proliferation and survival, cytokine production, and T-helper type-2 development. Several alternatively spliced transcript variants encoding different isoforms have been found for this gene.[provided by RefSeq, Jul 2011], | hsa04514:Cell adhesion molecules,hsa04660:T cell receptor signaling pathway,hsa04672:Intestinal immune network for IgA production,hsa04940:Type I diabetes mellitus,hsa05162:Measles,hsa05235:PD-L1 expression and PD-1 checkpoint pathway in cancer,hsa05320:Autoimmune thyroid disease,hsa05322:Systemic lupus erythematosus,hsa05323:Rheumatoid arthritis,hsa05330:Allograft rejection,hsa05332:Graft-versus-host disease,hsa05416:Viral myocarditis, |
| 11314 | CD300a molecule(CD300A) | This gene encodes a member of the CD300 glycoprotein family of cell surface proteins found on leukocytes involved in immune response signaling pathways. This gene is located on chromosome 17 in a cluster with all but one of the other family members. Multiple transcript variants encoding different isoforms have been found for this gene. [provided by RefSeq, Feb 2012], |  |
| 961 | CD47 molecule(CD47) | This gene encodes a membrane protein, which is involved in the increase in intracellular calcium concentration that occurs upon cell adhesion to extracellular matrix. The encoded protein is also a receptor for the C-terminal cell binding domain of thrombospondin, and it may play a role in membrane transport and signal transduction. This gene has broad tissue distribution, and is reduced in expression on Rh erythrocytes. Alternatively spliced transcript variants have been found for this gene. [provided by RefSeq, Jul 2010], | hsa04512:ECM-receptor interaction, |
| 963 | CD53 molecule(CD53) | The protein encoded by this gene is a member of the transmembrane 4 superfamily, also known as the tetraspanin family. Most of these members are cell-surface proteins that are characterized by the presence of four hydrophobic domains. The proteins mediate signal transduction events that play a role in the regulation of cell development, activation, growth and motility. This encoded protein is a cell surface glycoprotein that is known to complex with integrins. It contributes to the transduction of CD2-generated signals in T cells and natural killer cells and has been suggested to play a role in growth regulation. Familial deficiency of this gene has been linked to an immunodeficiency associated with recurrent infectious diseases caused by bacteria, fungi and viruses. Alternative splicing results in multiple transcript variants. [provided by RefSeq, Mar 2016], |  |
| 924 | CD7 molecule(CD7) | This gene encodes a transmembrane protein which is a member of the immunoglobulin superfamily. This protein is found on thymocytes and mature T cells. It plays an essential role in T-cell interactions and also in T-cell/B-cell interaction during early lymphoid development. [provided by RefSeq, Jul 2008], | hsa04640:Hematopoietic cell lineage, |
| 56990 | CDC42 small effector 2(CDC42SE2) | Enables signaling adaptor activity. Involved in regulation of signal transduction. Located in plasma membrane. [provided by Alliance of Genome Resources, Apr 2022], |  |
| 255919 | CTD nuclear envelope phosphatase 1 regulatory subunit 1(CNEP1R1) | This gene encodes a transmembrane protein that belongs to the Tmemb_18A family. A similar protein in yeast is a component of an endoplasmic reticulum-associated protein phosphatase complex and is thought to play a role in the synthesis of triacylglycerol. Alternate splicing results in multiple transcript variants. [provided by RefSeq, Aug 2013], |  |
| 10659 | CUGBP Elav-like family member 2(CELF2) | Members of the CELF/BRUNOL protein family contain two N-terminal RNA recognition motif (RRM) domains, one C-terminal RRM domain, and a divergent segment of 160-230 aa between the second and third RRM domains. Members of this protein family regulate pre-mRNA alternative splicing and may also be involved in mRNA editing, and translation. Alternative splicing results in multiple transcript variants encoding different isoforms. [provided by RefSeq, Jul 2008], |  |
| 51571 | CYFIP related Rac1 interactor B(CYRIB) | Enables small GTPase binding activity. Involved in several processes, including cellular response to molecule of bacterial origin; negative regulation of small GTPase mediated signal transduction; and regulation of organelle organization. Located in mitochondrion. [provided by Alliance of Genome Resources, Apr 2022], |  |
| 23259 | DDHD domain containing 2(DDHD2) | This gene encodes a phospholipase enzyme containing sterile-alpha-motif (SAM), WWE, and DDHD domains. This protein participates in membrane trafficking between the endoplastic reticulum and the Golgi body. Mutations in this gene can cause autosomal recessive spastic paraplegia 54. Alternative splicing results in multiple transcript variants. [provided by RefSeq, Dec 2013], |  |
| 79961 | DENN domain containing 2D(DENND2D) | Enables guanyl-nucleotide exchange factor activity. Predicted to be involved in regulation of catalytic activity. Located in cytosol and nucleoplasm. [provided by Alliance of Genome Resources, Apr 2022], |  |
| 22898 | DENN domain containing 3(DENND3) | Enables guanyl-nucleotide exchange factor activity. Predicted to be involved in cellular protein catabolic process; endosome to lysosome transport; and regulation of Rab protein signal transduction. Predicted to be located in cytosol. Predicted to be active in cytoplasmic vesicle. [provided by Alliance of Genome Resources, Apr 2022], |  |
| 23258 | DENN domain containing 5A(DENND5A) | This gene encodes a DENN-domain-containing protein that functions as a RAB-activating guanine nucleotide exchange factor (GEF). This protein catalyzes the conversion of GDP to GTP and thereby converts inactive GDP-bound Rab proteins into their active GTP-bound form. The encoded protein is recruited by RAB6 onto Golgi membranes and is therefore referred to as RAB6-interacting protein 1. This protein binds with RAB39 as well. Alternative splicing results in multiple transcript variants encoding distinct isoforms. Mutations in this gene are associated with early infantile epileptic encephalopathy-49. [provided by RefSeq, Feb 2017], |  |
| 10212 | DExD-box helicase 39A(DDX39A) | This gene encodes a member of the DEAD box protein family. These proteins are characterized by the conserved motif Asp-Glu-Ala-Asp (DEAD) and are putative RNA helicases. They are implicated in a number of cellular processes involving alteration of RNA secondary structure, such as translation initiation, nuclear and mitochondrial splicing, and ribosome and spliceosome assembly. Based on their distribution patterns, some members of the DEAD box protein family are believed to be involved in embryogenesis, spermatogenesis, and cellular growth and division. This gene is thought to play a role in the prognosis of patients with gastrointestinal stromal tumors. A pseudogene of this gene is present on chromosome 13. Alternate splicing results in multiple transcript variants. Additional alternatively spliced transcript variants of this gene have been described, but their full-length nature is not known. [provided by RefSeq, Sep 2013], |  |
| 1997 | E74 like ETS transcription factor 1(ELF1) | This gene encodes an E26 transformation-specific related transcription factor. The encoded protein is primarily expressed in lymphoid cells and acts as both an enhancer and a repressor to regulate transcription of various genes. Alternative splicing results in multiple transcript variants. [provided by RefSeq, Feb 2009], |  |
| 285203 | EGF domain specific O-linked N-acetylglucosamine transferase(EOGT) | This gene encodes an enzyme that acts in the lumen of the endoplasmic reticulum to catalyze the transfer of N-acetylglucosamine to serine or threonine residues of extracellular-targeted proteins. This enzyme modifies proteins containing eukaryotic growth factor (EGF)-like domains, including the Notch receptor, thereby regulating developmental signalling. Mutations in this gene have been observed in individuals with Adams-Oliver syndrome 4. Alternative splicing results in multiple transcript variants. [provided by RefSeq, Aug 2015], | hsa00514:Other types of O-glycan biosynthesis, |
| 11160 | ER lipid raft associated 2(ERLIN2) | This gene encodes a member of the SPFH domain-containing family of lipid raft-associated proteins. The encoded protein is localized to lipid rafts of the endoplasmic reticulum and plays a critical role in inositol 1,4,5-trisphosphate (IP3) signaling by mediating ER-associated degradation of activated IP3 receptors. Mutations in this gene are a cause of spastic paraplegia-18 (SPG18). Alternatively spliced transcript variants encoding multiple isoforms have been observed for this gene. [provided by RefSeq, Feb 2012], |  |
| 2114 | ETS proto-oncogene 2, transcription factor(ETS2) | This gene encodes a transcription factor which regulates genes involved in development and apoptosis. The encoded protein is also a protooncogene and shown to be involved in regulation of telomerase. A pseudogene of this gene is located on the X chromosome. Alternative splicing results in multiple transcript variants. [provided by RefSeq, Jan 2012], | hsa04014:Ras signaling pathway,hsa05166:Human T-cell leukemia virus 1 infection, |
| 26234 | F-box and leucine rich repeat protein 5(FBXL5) | This gene encodes a member of the F-box protein family which is characterized by an approximately 40 amino acid motif, the F-box. The F-box proteins constitute one of the four subunits of ubiquitin protein ligase complex called SCFs (SKP1-cullin-F-box), which function in phosphorylation-dependent ubiquitination. The F-box proteins are divided into 3 classes: Fbws containing WD-40 domains, Fbls containing leucine-rich repeats, and Fbxs containing either different protein-protein interaction modules or no recognizable motifs. The protein encoded by this gene belongs to the Fbls class and, in addition to an F-box, contains several tandem leucine-rich repeats. Alternatively spliced transcript variants have been described for this locus. [provided by RefSeq, Aug 2010], |  |
| 10447 | FAM3 metabolism regulating signaling molecule C(FAM3C) | This gene is a member of the family with sequence similarity 3 (FAM3) family and encodes a secreted protein with a GG domain. A change in expression of this protein has been noted in pancreatic cancer-derived cells. [provided by RefSeq, Mar 2010], |  |
| 2268 | FGR proto-oncogene, Src family tyrosine kinase(FGR) | This gene is a member of the Src family of protein tyrosine kinases (PTKs). The encoded protein contains N-terminal sites for myristylation and palmitylation, a PTK domain, and SH2 and SH3 domains which are involved in mediating protein-protein interactions with phosphotyrosine-containing and proline-rich motifs, respectively. The protein localizes to plasma membrane ruffles, and functions as a negative regulator of cell migration and adhesion triggered by the beta-2 integrin signal transduction pathway. Infection with Epstein-Barr virus results in the overexpression of this gene. Multiple alternatively spliced variants, encoding the same protein, have been identified. [provided by RefSeq, Jul 2008], | hsa04062:Chemokine signaling pathway, |
| 85476 | G elongation factor mitochondrial 1(GFM1) | Eukaryotes contain two protein translational systems, one in the cytoplasm and one in the mitochondria. Mitochondrial translation is crucial for maintaining mitochondrial function and mutations in this system lead to a breakdown in the respiratory chain-oxidative phosphorylation system and to impaired maintenance of mitochondrial DNA. This gene encodes one of the mitochondrial translation elongation factors. Its role in the regulation of normal mitochondrial function and in different disease states attributed to mitochondrial dysfunction is not known. [provided by RefSeq, Jul 2008], |  |
| 63940 | G protein signaling modulator 3(GPSM3) | Predicted to enable GTPase regulator activity. Predicted to be involved in positive regulation of inflammatory response. Predicted to act upstream of or within positive regulation of cytokine production involved in inflammatory response and positive regulation of leukocyte chemotaxis. Predicted to be located in cytoplasm and plasma membrane. [provided by Alliance of Genome Resources, Apr 2022], |  |
| 2788 | G protein subunit gamma 7(GNG7) | Predicted to enable G-protein beta-subunit binding activity. Predicted to be involved in G protein-coupled receptor signaling pathway and regulation of adenylate cyclase activity. Predicted to act upstream of or within behavioral fear response; locomotory behavior; and receptor guanylyl cyclase signaling pathway. Located in extracellular exosome. [provided by Alliance of Genome Resources, Apr 2022], | hsa04014:Ras signaling pathway,hsa04062:Chemokine signaling pathway,hsa04151:PI3K-Akt signaling pathway,hsa04371:Apelin signaling pathway,hsa04713:Circadian entrainment,hsa04723:Retrograde endocannabinoid signaling,hsa04724:Glutamatergic synapse,hsa04725:Cholinergic synapse,hsa04726:Serotonergic synapse,hsa04727:GABAergic synapse,hsa04728:Dopaminergic synapse,hsa04740:Olfactory transduction,hsa04926:Relaxin signaling pathway,hsa05032:Morphine addiction,hsa05034:Alcoholism,hsa05163:Human cytomegalovirus infection,hsa05167:Kaposi sarcoma-associated herpesvirus infection,hsa05170:Human immunodeficiency virus 1 infection,hsa05200:Pathways in cancer, |
| 7107 | G protein-coupled receptor 137B(GPR137B) | Involved in several processes, including positive regulation of TORC1 signaling; positive regulation of protein localization to lysosome; and regulation of GTPase activity. Located in lysosomal membrane. [provided by Alliance of Genome Resources, Apr 2022], |  |
| 151556 | G protein-coupled receptor 155(GPR155) | Involved in cognition. Located in extracellular exosome. [provided by Alliance of Genome Resources, Apr 2022], |  |
| 1880 | G protein-coupled receptor 183(GPR183) | This gene was identified by the up-regulation of its expression upon Epstein-Barr virus infection of primary B lymphocytes. This gene is predicted to encode a G protein-coupled receptor that is most closely related to the thrombin receptor. Expression of this gene was detected in B-lymphocyte cell lines and lymphoid tissues but not in T-lymphocyte cell lines or peripheral blood T lymphocytes. The function of this gene is unknown. [provided by RefSeq, Jul 2008], |  |
| 51291 | GEM interacting protein(GMIP) | This gene encodes a member of the ARHGAP family of Rho/Rac/Cdc42-like GTPase activating proteins. The encoded protein interacts with the Ras-related protein Gem through its N-terminal domain. Separately, it interacts with RhoA through a RhoGAP domain, and stimulates RhoA-dependent GTPase activity. Alternative splicing results in multiple transcript variants. [provided by RefSeq, Jan 2014], |  |
| 26157 | GTPase, IMAP family member 2(GIMAP2) | This gene encodes a protein belonging to the GTP-binding superfamily and to the immuno-associated nucleotide (IAN) subfamily of nucleotide-binding proteins. In humans, the IAN subfamily genes are located in a cluster at 7q36.1. [provided by RefSeq, Jul 2008], |  |
| 55303 | GTPase, IMAP family member 4(GIMAP4) | This gene encodes a protein belonging to the GTP-binding superfamily and to the immuno-associated nucleotide (IAN) subfamily of nucleotide-binding proteins. The encoded protein of this gene may be negatively regulated by T-cell acute lymphocytic leukemia 1 (TAL1). In humans, the IAN subfamily genes are located in a cluster at 7q36.1. [provided by RefSeq, Jul 2008], |  |
| 3055 | HCK proto-oncogene, Src family tyrosine kinase(HCK) | The protein encoded by this gene is a member of the Src family of tyrosine kinases. This protein is primarily hemopoietic, particularly in cells of the myeloid and B-lymphoid lineages. It may help couple the Fc receptor to the activation of the respiratory burst. In addition, it may play a role in neutrophil migration and in the degranulation of neutrophils. Multiple isoforms with different subcellular distributions are produced due to both alternative splicing and the use of alternative translation initiation codons, including a non-AUG (CUG) codon. [provided by RefSeq, Feb 2010], | hsa04062:Chemokine signaling pathway,hsa04666:Fc gamma R-mediated phagocytosis,hsa05167:Kaposi sarcoma-associated herpesvirus infection, |
| 26091 | HECT and RLD domain containing E3 ubiquitin protein ligase 4(HERC4) | HERC4 belongs to the HERC family of ubiquitin ligases, all of which contain a HECT domain and at least 1 RCC1 (MIM 179710)-like domain (RLD). The 350-amino acid HECT domain is predicted to catalyze the formation of a thioester with ubiquitin before transferring it to a substrate, and the RLD is predicted to act as a guanine nucleotide exchange factor for small G proteins (Hochrainer et al., 2005 [PubMed 15676274]).[supplied by OMIM, Mar 2008], | hsa04120:Ubiquitin mediated proteolysis, |
| 22806 | IKAROS family zinc finger 3(IKZF3) | This gene encodes a member of the Ikaros family of zinc-finger proteins. Three members of this protein family (Ikaros, Aiolos and Helios) are hematopoietic-specific transcription factors involved in the regulation of lymphocyte development. This gene product is a transcription factor that is important in the regulation of B lymphocyte proliferation and differentiation. Both Ikaros and Aiolos can participate in chromatin remodeling. Regulation of gene expression in B lymphocytes by Aiolos is complex as it appears to require the sequential formation of Ikaros homodimers, Ikaros/Aiolos heterodimers, and Aiolos homodimers. Several alternative transcripts encoding different isoforms have been described, as well as some non-protein coding variants. [provided by RefSeq, Apr 2012], |  |
| 3702 | IL2 inducible T cell kinase(ITK) | This gene encodes an intracellular tyrosine kinase expressed in T-cells. The protein contains both SH2 and SH3 domains which are often found in intracellular kinases. It is thought to play a role in T-cell proliferation and differentiation. [provided by RefSeq, Jul 2008], | hsa04062:Chemokine signaling pathway,hsa04660:T cell receptor signaling pathway,hsa04670:Leukocyte transendothelial migration, |
| 8826 | IQ motif containing GTPase activating protein 1(IQGAP1) | This gene encodes a member of the IQGAP family. The protein contains four IQ domains, one calponin homology domain, one Ras-GAP domain and one WW domain. It interacts with components of the cytoskeleton, with cell adhesion molecules, and with several signaling molecules to regulate cell morphology and motility. Expression of the protein is upregulated by gene amplification in two gastric cancer cell lines. [provided by RefSeq, Jul 2008], | hsa04520:Adherens junction,hsa04810:Regulation of actin cytoskeleton,hsa05205:Proteoglycans in cancer, |
| 10788 | IQ motif containing GTPase activating protein 2(IQGAP2) | This gene encodes a member of the IQGAP family. The encoded protein contains three IQ domains, one calponin homology domain, one Ras-GAP domain and one WW domain. This protein interacts with components of the cytoskeleton, with cell adhesion molecules, and with several signaling molecules to regulate cell morphology and motility. It also acts as a tumor suppressor and has been found to play a role in regulating innate antiviral responses. Alternative splicing results in multiple transcript variants. [provided by RefSeq, Apr 2017], | hsa04810:Regulation of actin cytoskeleton, |
| 79932 | KIAA0319 like(KIAA0319L) | This gene is a candidate gene for dyslexia susceptibility.[provided by RefSeq, Apr 2009], |  |
| 81606 | LBH regulator of WNT signaling pathway(LBH) | Involved in negative regulation of transcription, DNA-templated; positive regulation of transcription, DNA-templated; and regulation of MAPK cascade. Located in cytoplasm and nucleus. Part of protein-containing complex. [provided by Alliance of Genome Resources, Apr 2022], |  |
| 55646 | Ly1 antibody reactive(LYAR) | Enables several functions, including DNA-binding transcription factor binding activity; identical protein binding activity; and transcription regulator inhibitor activity. Involved in several processes, including erythrocyte development; negative regulation of innate immune response; and regulation of transcription, DNA-templated. Located in nucleolus and nucleoplasm. [provided by Alliance of Genome Resources, Apr 2022], |  |
| 10198 | M-phase phosphoprotein 9(MPHOSPH9) | Located in Golgi apparatus and centriole. Implicated in multiple sclerosis. [provided by Alliance of Genome Resources, Apr 2022], |  |
| 4354 | MAGUK p55 scaffold protein 1(MPP1) | This gene encodes the prototype of the membrane-associated guanylate kinase (MAGUK) family proteins. MAGUKs interact with the cytoskeleton and regulate cell proliferation, signaling pathways, and intercellular junctions. The encoded protein is an extensively palmitoylated membrane phosphoprotein containing a PDZ domain, a Src homology 3 (SH3) motif, and a guanylate kinase domain. This gene product interacts with various cytoskeletal proteins and cell junctional proteins in different tissue and cell types, and may be involved in the regulation of cell shape, hair cell development, neural patterning of the retina, and apico-basal polarity and tumor suppression pathways in non-erythroid cells. Multiple transcript variants encoding different isoforms have been found for this gene. [provided by RefSeq, Oct 2009], |  |
| 84549 | MAK16 homolog(MAK16) | Enables RNA binding activity. Predicted to be involved in maturation of 5.8S rRNA and maturation of LSU-rRNA. Located in nucleolus. [provided by Alliance of Genome Resources, Apr 2022], |  |
| 4277 | MHC class I polypeptide-related sequence B(MICB) | This gene encodes a heavily glycosylated protein which is a ligand for the NKG2D type II receptor. Binding of the ligand activates the cytolytic response of natural killer (NK) cells, CD8 alphabeta T cells, and gammadelta T cells which express the receptor. This protein is stress-induced and is similar to MHC class I molecules; however, it does not associate with beta-2-microglobulin or bind peptides. Alternative splicing results in multiple transcript variants. [provided by RefSeq, Jan 2014], | hsa04650:Natural killer cell mediated cytotoxicity,hsa05167:Kaposi sarcoma-associated herpesvirus infection, |
| 64210 | MMS19 homolog, cytosolic iron-sulfur assembly component(MMS19) | Enables estrogen receptor binding activity and transcription coactivator activity. Involved in several processes, including iron-sulfur cluster assembly; positive regulation of nucleobase-containing compound metabolic process; and protein maturation by iron-sulfur cluster transfer. Located in cytosol; nucleoplasm; and spindle. Part of CIA complex and MMXD complex. [provided by Alliance of Genome Resources, Apr 2022], |  |
| 4600 | MX dynamin like GTPase 2(MX2) | The protein encoded by this gene has a nuclear and a cytoplasmic form and is a member of both the dynamin family and the family of large GTPases. The nuclear form is localized in a granular pattern in the heterochromatin region beneath the nuclear envelope. A nuclear localization signal (NLS) is present at the amino terminal end of the nuclear form but is lacking in the cytoplasmic form due to use of an alternate translation start codon. This protein is upregulated by interferon-alpha but does not contain the antiviral activity of a similar myxovirus resistance protein 1. [provided by RefSeq, Jul 2008], | hsa03250:Viral life cycle - HIV-1,hsa05160:Hepatitis C,hsa05162:Measles,hsa05164:Influenza A,hsa05165:Human papillomavirus infection,hsa05171:Coronavirus disease - COVID-19, |
| 80896 | N-acetylneuraminate pyruvate lyase(NPL) | This gene encodes a member of the N-acetylneuraminate lyase sub-family of (beta/alpha)(8)-barrel enzymes. N-acetylneuraminate lyases regulate cellular concentrations of N-acetyl-neuraminic acid (sialic acid) by mediating the reversible conversion of sialic acid into N-acetylmannosamine and pyruvate. A pseudogene of this gene is located on the short arm of chromosome 2. Alternatively spliced transcript variants encoding multiple isoforms have been observed for this gene. [provided by RefSeq, Jan 2011], | hsa00520:Amino sugar and nucleotide sugar metabolism,hsa01100:Metabolic pathways, |
| 80218 | N-alpha-acetyltransferase 50, NatE catalytic subunit(NAA50) | Enables H4 histone acetyltransferase activity; peptide alpha-N-acetyltransferase activity; and peptidyl-lysine acetyltransferase activity. Involved in N-terminal protein amino acid acetylation; establishment of mitotic sister chromatid cohesion; and mitotic sister chromatid cohesion, centromeric. Located in cytosol and nucleus. Part of NatA complex. [provided by Alliance of Genome Resources, Apr 2022], |  |
| 9111 | N-myc and STAT interactor(NMI) | NMYC interactor (NMI) encodes a protein that interacts with NMYC and CMYC (two members of the oncogene Myc family), and other transcription factors containing a Zip, HLH, or HLH-Zip motif. The NMI protein also interacts with all STATs except STAT2 and augments STAT-mediated transcription in response to cytokines IL2 and IFN-gamma. The NMI mRNA has low expression levels in all human fetal and adult tissues tested except brain and has high expression in cancer cell line-myeloid leukemias. [provided by RefSeq, Jul 2008], |  |
| 3071 | NCK associated protein 1 like(NCKAP1L) | This gene encodes a member of the HEM family of tissue-specific transmembrane proteins which are highly conserved from invertebrates through mammals. This gene is only expressed in hematopoietic cells. The encoded protein is a part of the Scar/WAVE complex which plays an important role in regulating cell shape in both metazoans and plants. Alternatively spliced transcript variants encoding different isoforms have been found.[provided by RefSeq, May 2010], | hsa04810:Regulation of actin cytoskeleton,hsa05130:Pathogenic Escherichia coli infection,hsa05132:Salmonella infection, |
| 9683 | NEDD4 binding protein 1(N4BP1) | Enables mRNA binding activity; ribonuclease activity; and ubiquitin binding activity. Involved in cellular response to UV and negative regulation of viral genome replication. Predicted to be located in cytosol and nucleolus. Predicted to be active in PML body. [provided by Alliance of Genome Resources, Apr 2022], |  |
| 4664 | NGFI-A binding protein 1(NAB1) | Predicted to enable transcription coregulator activity. Predicted to be involved in regulation of transcription, DNA-templated. Predicted to act upstream of or within endochondral ossification; nervous system development; and regulation of epidermis development. Predicted to be located in nucleoplasm. Predicted to be active in nucleus. [provided by Alliance of Genome Resources, Apr 2022], |  |
| 64318 | NOC3 like DNA replication regulator(NOC3L) | Enables RNA binding activity. Predicted to be involved in DNA replication initiation. Predicted to act upstream of or within fat cell differentiation. Located in mitochondrion; nucleolus; and nucleoplasm. [provided by Alliance of Genome Resources, Apr 2022], |  |
| 5090 | PBX homeobox 3(PBX3) | Predicted to enable DNA-binding transcription factor activity, RNA polymerase II-specific and RNA polymerase II cis-regulatory region sequence-specific DNA binding activity. Predicted to be involved in animal organ development; neuron development; and regulation of transcription by RNA polymerase II. Predicted to act upstream of or within several processes, including adult locomotory behavior; dorsal spinal cord development; and regulation of respiratory gaseous exchange by nervous system process. Predicted to be located in nucleus. Predicted to be part of chromatin. [provided by Alliance of Genome Resources, Apr 2022], | hsa05202:Transcriptional misregulation in cancer, |
| 51131 | PHD finger protein 11(PHF11) | This gene encodes a protein containing a PHD (plant homeodomain) type zinc finger. This gene has been identified in some studies as a candidate gene for asthma. Naturally-occurring readthrough transcription may occur from the upstream SETDB2 (SET domain bifurcated 2) gene to this locus. Alternative splicing results in multiple transcript variants. [provided by RefSeq, Mar 2016], |  |
| 84106 | PML-RARA regulated adaptor molecule 1(PRAM1) | The protein encoded by this gene is similar to FYN binding protein (FYB/SLAP-130), an adaptor protein involved in T cell receptor mediated signaling. This gene is expressed and regulated during normal myelopoiesis. The expression of this gene is induced by retinoic acid and is inhibited by the expression of PML-RARalpha, a fusion protein of promyelocytic leukemia (PML) and the retinoic acid receptor-alpha (RARalpha). [provided by RefSeq, Jul 2008], |  |
| 9240 | PNMA family member 1(PNMA1) | This gene encodes a neuron- and testis-specific protein that is also expressed in some paraneoplastic syndromes affecting the nervous system. Some patients with neurologic disorders develop antibodies against the protein encoded by this gene. The identification of the antineuronal antibodies in the sera of these patients has facilitated the diagnosis of paraneoplastic neurological disorders and the early detection of the associated tumors. [provided by RefSeq, Feb 2014], |  |
| 29108 | PYD and CARD domain containing(PYCARD) | This gene encodes an adaptor protein that is composed of two protein-protein interaction domains: a N-terminal PYRIN-PAAD-DAPIN domain (PYD) and a C-terminal caspase-recruitment domain (CARD). The PYD and CARD domains are members of the six-helix bundle death domain-fold superfamily that mediates assembly of large signaling complexes in the inflammatory and apoptotic signaling pathways via the activation of caspase. In normal cells, this protein is localized to the cytoplasm; however, in cells undergoing apoptosis, it forms ball-like aggregates near the nuclear periphery. Two transcript variants encoding different isoforms have been found for this gene. [provided by RefSeq, Jul 2008], | hsa04217:Necroptosis,hsa04621:NOD-like receptor signaling pathway,hsa04623:Cytosolic DNA-sensing pathway,hsa04625:C-type lectin receptor signaling pathway,hsa05130:Pathogenic Escherichia coli infection,hsa05131:Shigellosis,hsa05132:Salmonella infection,hsa05133:Pertussis,hsa05134:Legionellosis,hsa05135:Yersinia infection,hsa05164:Influenza A,hsa05417:Lipid and atherosclerosis, |
| 11040 | Pim-2 proto-oncogene, serine/threonine kinase(PIM2) | This gene encodes a protooncogene that acts as a serine/threonine protein kinase. Studies determined the encoded protein functions to prevent apoptosis and to promote cell survival.[provided by RefSeq, Nov 2009], | hsa05200:Pathways in cancer,hsa05221:Acute myeloid leukemia, |
| 9444 | QKI, KH domain containing RNA binding(QKI) | The protein encoded by this gene is an RNA-binding protein that regulates pre-mRNA splicing, export of mRNAs from the nucleus, protein translation, and mRNA stability. The encoded protein is involved in myelinization and oligodendrocyte differentiation and may play a role in schizophrenia. Multiple transcript variants encoding different isoforms have been found for this gene. [provided by RefSeq, Jul 2014], |  |
| 91300 | R3H domain containing 4(R3HDM4) | Predicted to enable nucleic acid binding activity. Predicted to be located in nucleus. [provided by Alliance of Genome Resources, Apr 2022], |  |
| 5873 | RAB27A, member RAS oncogene family(RAB27A) | The protein encoded by this gene belongs to the small GTPase superfamily, Rab family. The protein is membrane-bound and may be involved in protein transport and small GTPase mediated signal transduction. Mutations in this gene are associated with Griscelli syndrome type 2. Alternative splicing occurs at this locus and four transcript variants encoding the same protein have been identified. [provided by RefSeq, Jul 2008], |  |
| 11031 | RAB31, member RAS oncogene family(RAB31) | Small GTP-binding proteins of the RAB family, such as RAB31, play essential roles in vesicle and granule targeting (Bao et al., 2002 [PubMed 11784320]).[supplied by OMIM, Jul 2009], | hsa04144:Endocytosis, |
| 9363 | RAB33A, member RAS oncogene family(RAB33A) | The protein encoded by this gene belongs to the small GTPase superfamily, Rab family. It is GTP-binding protein and may be involved in vesicle transport. [provided by RefSeq, Jul 2008], |  |
| 57826 | RAP2C, member of RAS oncogene family(RAP2C) | The protein encoded by this gene is a member of the Ras-related protein subfamily of the Ras GTPase superfamily. Members of this family are small GTPases that act as molecular switches to regulate cellular proliferation, differentiation, and apoptosis. This protein has been reported to activate in vitro transcriptional activity of the serum response element. Alternative splicing results in multiple transcript variants. [provided by RefSeq, Sep 2012], | hsa04530:Tight junction, |
| 10125 | RAS guanyl releasing protein 1(RASGRP1) | This gene is a member of a family of genes characterized by the presence of a Ras superfamily guanine nucleotide exchange factor (GEF) domain. It functions as a diacylglycerol (DAG)-regulated nucleotide exchange factor specifically activating Ras through the exchange of bound GDP for GTP. It activates the Erk/MAP kinase cascade and regulates T-cells and B-cells development, homeostasis and differentiation. Alternatively spliced transcript variants encoding different isoforms have been identified. Altered expression of the different isoforms of this protein may be a cause of susceptibility to systemic lupus erythematosus (SLE). [provided by RefSeq, Jul 2008], | hsa04010:MAPK signaling pathway,hsa04014:Ras signaling pathway,hsa04611:Platelet activation,hsa04660:T cell receptor signaling pathway,hsa05200:Pathways in cancer,hsa05235:PD-L1 expression and PD-1 checkpoint pathway in cancer, |
| 10235 | RAS guanyl releasing protein 2(RASGRP2) | The protein encoded by this gene is a brain-enriched nucleotide exchanged factor that contains an N-terminal GEF domain, 2 tandem repeats of EF-hand calcium-binding motifs, and a C-terminal diacylglycerol/phorbol ester-binding domain. This protein can activate small GTPases, including RAS and RAP1/RAS3. The nucleotide exchange activity of this protein can be stimulated by calcium and diacylglycerol. Four alternatively spliced transcript variants encoding two different isoforms have been found for this gene. [provided by RefSeq, Jan 2016], | hsa04010:MAPK signaling pathway,hsa04014:Ras signaling pathway,hsa04015:Rap1 signaling pathway,hsa04062:Chemokine signaling pathway,hsa04611:Platelet activation,hsa05200:Pathways in cancer, |
| 5922 | RAS p21 protein activator 2(RASA2) | The protein encoded by this gene is member of the GAP1 family of GTPase-activating proteins. The gene product stimulates the GTPase activity of normal RAS p21 but not its oncogenic counterpart. Acting as a suppressor of RAS function, the protein enhances the weak intrinsic GTPase activity of RAS proteins resulting in the inactive GDP-bound form of RAS, thereby allowing control of cellular proliferation and differentiation. Alternative splicing results in multiple transcript variants. [provided by RefSeq, Dec 2014], | hsa04010:MAPK signaling pathway,hsa04014:Ras signaling pathway,hsa05203:Viral carcinogenesis, |
| 22821 | RAS p21 protein activator 3(RASA3) | This gene encodes a protein that binds inositol 1,3,4,5-tetrakisphosphate and stimulates the GTPase activity of Ras p21. This protein functions as a negative regulator of the Ras signalling pathway. It is localized to the cell membrane via a pleckstrin homology (PH) domain in the C-terminal region. Alternative splicing results in multiple transcript variants. [provided by RefSeq, Mar 2016], | hsa04014:Ras signaling pathway, |
| 9584 | RNA binding motif protein 39(RBM39) | This gene encodes a member of the U2AF65 family of proteins. The encoded protein is found in the nucleus, where it co-localizes with core spliceosomal proteins. It has been shown to play a role in both steroid hormone receptor-mediated transcription and alternative splicing, and it is also a transcriptional coregulator of the viral oncoprotein v-Rel. Multiple transcript variants have been observed for this gene. A related pseudogene has been identified on chromosome X. [provided by RefSeq, Aug 2011], |  |
| 84268 | RPA interacting protein(RPAIN) | Predicted to enable metal ion binding activity. Acts upstream of or within several processes, including DNA metabolic process; protein import into nucleus; and response to UV. Located in PML body; cytoplasm; and fibrillar center. [provided by Alliance of Genome Resources, Apr 2022], |  |
| 9459 | Rac/Cdc42 guanine nucleotide exchange factor 6(ARHGEF6) | Rho GTPases play a fundamental role in numerous cellular processes that are initiated by extracellular stimuli that work through G protein coupled receptors. The encoded protein belongs to a family of cytoplasmic proteins that activate the Ras-like family of Rho proteins by exchanging bound GDP for GTP. It may form a complex with G proteins and stimulate Rho-dependent signals. This protein is activated by PI3-kinase. Mutations in this gene can cause X-chromosomal non-specific cognitive disability. [provided by RefSeq, Jul 2008], | hsa04810:Regulation of actin cytoskeleton,hsa05212:Pancreatic cancer, |
| 57514 | Rho GTPase activating protein 31(ARHGAP31) | This gene encodes a GTPase-activating protein (GAP). A variety of cellular processes are regulated by Rho GTPases which cycle between an inactive form bound to GDP and an active form bound to GTP. This cycling between inactive and active forms is regulated by guanine nucleotide exchange factors and GAPs. The encoded protein is a GAP shown to regulate two GTPases involved in protein trafficking and cell growth. [provided by RefSeq, Jul 2008], |  |
| 6502 | S-phase kinase associated protein 2(SKP2) | This gene encodes a member of the F-box protein family which is characterized by an approximately 40 amino acid motif, the F-box. The F-box proteins constitute one of the four subunits of ubiquitin protein ligase complex called SCFs (SKP1-cullin-F-box), which function in phosphorylation-dependent ubiquitination. The F-box proteins are divided into 3 classes: Fbws containing WD-40 domains, Fbls containing leucine-rich repeats, and Fbxs containing either different protein-protein interaction modules or no recognizable motifs. The protein encoded by this gene belongs to the Fbls class; in addition to an F-box, this protein contains 10 tandem leucine-rich repeats. This protein is an essential element of the cyclin A-CDK2 S-phase kinase. It specifically recognizes phosphorylated cyclin-dependent kinase inhibitor 1B (CDKN1B, also referred to as p27 or KIP1) predominantly in S phase and interacts with S-phase kinase-associated protein 1 (SKP1 or p19). In addition, this gene is established as a protooncogene causally involved in the pathogenesis of lymphomas. Alternative splicing of this gene generates three transcript variants encoding different isoforms. [provided by RefSeq, Jul 2011], | hsa04068:FoxO signaling pathway,hsa04110:Cell cycle,hsa04120:Ubiquitin mediated proteolysis,hsa04150:mTOR signaling pathway,hsa05169:Epstein-Barr virus infection,hsa05200:Pathways in cancer,hsa05203:Viral carcinogenesis,hsa05222:Small cell lung cancer, |
| 55133 | S1 RNA binding domain 1(SRBD1) | Predicted to enable mRNA binding activity. Predicted to be a structural constituent of ribosome. Predicted to be involved in translation. [provided by Alliance of Genome Resources, Apr 2022], |  |
| 6282 | S100 calcium binding protein A11(S100A11) | The protein encoded by this gene is a member of the S100 family of proteins containing 2 EF-hand calcium-binding motifs. S100 proteins are localized in the cytoplasm and/or nucleus of a wide range of cells, and involved in the regulation of a number of cellular processes such as cell cycle progression and differentiation. S100 genes include at least 13 members which are located as a cluster on chromosome 1q21. This protein may function in motility, invasion, and tubulin polymerization. Chromosomal rearrangements and altered expression of this gene have been implicated in tumor metastasis. [provided by RefSeq, Jul 2008], |  |
| 6280 | S100 calcium binding protein A9(S100A9) | The protein encoded by this gene is a member of the S100 family of proteins containing 2 EF-hand calcium-binding motifs. S100 proteins are localized in the cytoplasm and/or nucleus of a wide range of cells, and involved in the regulation of a number of cellular processes such as cell cycle progression and differentiation. S100 genes include at least 13 members which are located as a cluster on chromosome 1q21. This protein may function in the inhibition of casein kinase and altered expression of this protein is associated with the disease cystic fibrosis. This antimicrobial protein exhibits antifungal and antibacterial activity. [provided by RefSeq, Nov 2014], | hsa04657:IL-17 signaling pathway, |
| 6397 | SEC14 like lipid binding 1(SEC14L1) | The protein encoded by this gene belongs to the SEC14 cytosolic factor family. It has similarity to yeast SEC14 and to Japanese flying squid RALBP which suggests a possible role of the gene product in an intracellular transport system. Multiple alternatively spliced transcript variants have been found for this gene; some variants represent read-through transcripts that include exons from the upstream gene C17orf86. [provided by RefSeq, Feb 2011], |  |
| 64754 | SET and MYND domain containing 3(SMYD3) | This gene encodes a histone methyltransferase which functions in RNA polymerase II complexes by an interaction with a specific RNA helicase. Multiple transcript variants encoding different isoforms have been found for this gene. [provided by RefSeq, Dec 2011], | hsa00310:Lysine degradation,hsa01100:Metabolic pathways, |
| 51100 | SH3 domain containing GRB2 like, endophilin B1(SH3GLB1) | This gene encodes a SRC homology 3 domain-containing protein. The encoded protein interacts with the proapoptotic member of the Bcl-2 family, Bcl-2-associated X protein (Bax) and may be involved in regulating apoptotic signaling pathways. This protein may also be involved in maintaining mitochondrial morphology. Alternate splicing results in multiple transcript variants. [provided by RefSeq, Sep 2011], | hsa04140:Autophagy - animal,hsa04144:Endocytosis, |
| 8869 | ST3 beta-galactoside alpha-2,3-sialyltransferase 5(ST3GAL5) | Ganglioside GM3 is known to participate in the induction of cell differentiation, modulation of cell proliferation, maintenance of fibroblast morphology, signal transduction, and integrin-mediated cell adhesion. The protein encoded by this gene is a type II membrane protein which catalyzes the formation of GM3 using lactosylceramide as the substrate. The encoded protein is a member of glycosyltransferase family 29 and may be localized to the Golgi apparatus. Mutation in this gene has been associated with Amish infantile epilepsy syndrome. Transcript variants encoding different isoforms have been found for this gene. [provided by RefSeq, Jul 2008], | hsa00604:Glycosphingolipid biosynthesis - ganglio series,hsa01100:Metabolic pathways, |
| 10312 | T cell immune regulator 1, ATPase H+ transporting V0 subunit a3(TCIRG1) | This gene encodes a subunit of a large protein complex known as a vacuolar H+-ATPase (V-ATPase). The protein complex acts as a pump to move protons across the membrane. This movement of protons helps regulate the pH of cells and their surrounding environment. V-ATPase dependent organelle acidification is necessary for such intracellular processes as protein sorting, zymogen activation, and receptor-mediated endocytosis. V-ATPase is comprised of a cytosolic V1 domain and a transmembrane V0 domain. Alternative splicing results in multiple transcript variants. Mutations in this gene are associated with infantile malignant osteopetrosis. [provided by RefSeq, May 2017], | hsa00190:Oxidative phosphorylation,hsa01100:Metabolic pathways,hsa04142:Lysosome,hsa04145:Phagosome,hsa04721:Synaptic vesicle cycle,hsa04966:Collecting duct acid secretion,hsa05110:Vibrio cholerae infection,hsa05120:Epithelial cell signaling in Helicobacter pylori infection,hsa05152:Tuberculosis,hsa05165:Human papillomavirus infection,hsa05323:Rheumatoid arthritis, |
| 201633 | T cell immunoreceptor with Ig and ITIM domains(TIGIT) | This gene encodes a member of the PVR (poliovirus receptor) family of immunoglobin proteins. The product of this gene is expressed on several classes of T cells including follicular B helper T cells (TFH). The protein has been shown to bind PVR with high affinity; this binding is thought to assist interactions between TFH and dendritic cells to regulate T cell dependent B cell responses.[provided by RefSeq, Sep 2009], | hsa04514:Cell adhesion molecules, |
| 57533 | TBC1 domain family member 14(TBC1D14) | Enables protein kinase binding activity. Involved in negative regulation of autophagy; recycling endosome to Golgi transport; and regulation of autophagosome assembly. Located in several cellular components, including Golgi apparatus; autophagosome; and recycling endosome. [provided by Alliance of Genome Resources, Apr 2022], |  |
| 26277 | TERF1 interacting nuclear factor 2(TINF2) | This gene encodes one of the proteins of the shelterin, or telosome, complex which protects telomeres by allowing the cell to distinguish between telomeres and regions of DNA damage. The protein encoded by this gene is a critical part of shelterin; it interacts with the three DNA-binding proteins of the shelterin complex, and it is important for assembly of the complex. Mutations in this gene cause dyskeratosis congenita (DKC), an inherited bone marrow failure syndrome. [provided by RefSeq, Mar 2010], |  |
| 54962 | TIMELESS interacting protein(TIPIN) | The protein encoded by this gene is part of the replisome complex, a group of proteins that support DNA replication. It binds TIM, which is involved in circadian rhythm regulation, and aids in protecting cells against DNA damage and stress. Two pseudogenes and two transcript variants encoding different isoforms have been found for this gene. [provided by RefSeq, Jul 2014], |  |
| 7090 | TLE family member 3, transcriptional corepressor(TLE3) | This gene encodes a transcriptional co-repressor protein that belongs to the transducin-like enhancer family of proteins. The members of this family function in the Notch signaling pathway that regulates determination of cell fate during development. Expression of this gene has been associated with a favorable outcome to chemotherapy with taxanes for ovarian carcinoma. Alternate splicing results in multiple transcript variants. Additional alternatively spliced transcript variants of this gene have been described, but their full-length nature is not known. [provided by RefSeq, Sep 2013], | hsa04310:Wnt signaling pathway,hsa04330:Notch signaling pathway, |
| 944 | TNF superfamily member 8(TNFSF8) | The protein encoded by this gene is a cytokine that belongs to the tumor necrosis factor (TNF) ligand family. This cytokine is a ligand for TNFRSF8/CD30, which is a cell surface antigen and a marker for Hodgkin lymphoma and related hematologic malignancies. The engagement of this cytokine expressed on B cell surface plays an inhibitory role in modulating Ig class switch. This cytokine was shown to enhance cell proliferation of some lymphoma cell lines, while to induce cell death and reduce cell proliferation of other lymphoma cell lines. The pleiotropic biologic activities of this cytokine on different CD30+ lymphoma cell lines may play a pathophysiologic role in Hodgkin&apos;s and some non-Hodgkin&apos;s lymphomas. Two transcript variants encoding different isoforms have been found for this gene. [provided by RefSeq, Nov 2011], | hsa04060:Cytokine-cytokine receptor interaction, |
| 11078 | TRIO and F-actin binding protein(TRIOBP) | This gene encodes a protein with an N-terminal pleckstrin homology domain and a C-terminal coiled-coil region. The protein interacts with trio, which is involved with neural tissue development and controlling actin cytoskeleton organization, cell motility and cell growth. The protein also associates with F-actin and stabilizes F-actin structures. Mutations in this gene have been associated with a form of autosomal recessive nonsyndromic deafness. Multiple alternatively spliced transcript variants that would encode different isoforms have been found for this gene, however some transcripts may be subject to nonsense-mediated decay (NMD). [provided by RefSeq, Nov 2008], |  |
| 79650 | U6 snRNA biogenesis phosphodiesterase 1(USB1) | This gene encodes a protein with several conserved domains, however, its exact function is not known. Mutations in this gene are associated with poikiloderma with neutropenia (PN), which shows phenotypic overlap with Rothmund-Thomson syndrome (RTS) caused by mutations in the RECQL4 gene. It is believed that this gene product interacts with RECQL4 protein via SMAD4 proteins, explaining the partial clinical overlap between PN and RTS. Alternatively spliced transcript variants encoding different isoforms have been noted for this gene. [provided by RefSeq, Mar 2011], |  |
| 7456 | WAS/WASL interacting protein family member 1(WIPF1) | This gene encodes a protein that plays an important role in the organization of the actin cytoskeleton. The encoded protein binds to a region of Wiskott-Aldrich syndrome protein that is frequently mutated in Wiskott-Aldrich syndrome, an X-linked recessive disorder. Impairment of the interaction between these two proteins may contribute to the disease. Two transcript variants encoding the same protein have been identified for this gene. [provided by RefSeq, Jul 2008], | hsa04144:Endocytosis,hsa05130:Pathogenic Escherichia coli infection,hsa05135:Yersinia infection, |
| 51099 | abhydrolase domain containing 5, lysophosphatidic acid acyltransferase(ABHD5) | The protein encoded by this gene belongs to a large family of proteins defined by an alpha/beta hydrolase fold, and contains three sequence motifs that correspond to a catalytic triad found in the esterase/lipase/thioesterase subfamily. It differs from other members of this subfamily in that its putative catalytic triad contains an asparagine instead of the serine residue. Mutations in this gene have been associated with Chanarin-Dorfman syndrome, a triglyceride storage disease with impaired long-chain fatty acid oxidation. [provided by RefSeq, Jul 2008], | hsa04923:Regulation of lipolysis in adipocytes, |
| 10096 | actin related protein 3(ACTR3) | The specific function of this gene has not yet been determined; however, the protein it encodes is known to be a major constituent of the ARP2/3 complex. This complex is located at the cell surface and is essential to cell shape and motility through lamellipodial actin assembly and protrusion. Three transcript variants encoding two different isoforms have been found for this gene. [provided by RefSeq, Mar 2013], | hsa04144:Endocytosis,hsa04530:Tight junction,hsa04666:Fc gamma R-mediated phagocytosis,hsa04810:Regulation of actin cytoskeleton,hsa05100:Bacterial invasion of epithelial cells,hsa05130:Pathogenic Escherichia coli infection,hsa05131:Shigellosis,hsa05132:Salmonella infection,hsa05135:Yersinia infection, |
| 87 | actinin alpha 1(ACTN1) | Alpha actinins belong to the spectrin gene superfamily which represents a diverse group of cytoskeletal proteins, including the alpha and beta spectrins and dystrophins. Alpha actinin is an actin-binding protein with multiple roles in different cell types. In nonmuscle cells, the cytoskeletal isoform is found along microfilament bundles and adherens-type junctions, where it is involved in binding actin to the membrane. In contrast, skeletal, cardiac, and smooth muscle isoforms are localized to the Z-disc and analogous dense bodies, where they help anchor the myofibrillar actin filaments. This gene encodes a nonmuscle, cytoskeletal, alpha actinin isoform and maps to the same site as the structurally similar erythroid beta spectrin gene. Three transcript variants encoding different isoforms have been found for this gene. [provided by RefSeq, Jul 2008], | hsa04510:Focal adhesion,hsa04520:Adherens junction,hsa04530:Tight junction,hsa04670:Leukocyte transendothelial migration,hsa04810:Regulation of actin cytoskeleton,hsa05131:Shigellosis,hsa05146:Amoebiasis,hsa05203:Viral carcinogenesis,hsa05322:Systemic lupus erythematosus, |
| 2180 | acyl-CoA synthetase long chain family member 1(ACSL1) | The protein encoded by this gene is an isozyme of the long-chain fatty-acid-coenzyme A ligase family. Although differing in substrate specificity, subcellular localization, and tissue distribution, all isozymes of this family convert free long-chain fatty acids into fatty acyl-CoA esters, and thereby play a key role in lipid biosynthesis and fatty acid degradation. Several transcript variants encoding different isoforms have been found for this gene. [provided by RefSeq, Nov 2013], | hsa00061:Fatty acid biosynthesis,hsa00071:Fatty acid degradation,hsa01100:Metabolic pathways,hsa01212:Fatty acid metabolism,hsa03320:PPAR signaling pathway,hsa04146:Peroxisome,hsa04216:Ferroptosis,hsa04714:Thermogenesis,hsa04920:Adipocytokine signaling pathway, |
| 262 | adenosylmethionine decarboxylase 1(AMD1) | This gene encodes an important intermediate enzyme in polyamine biosynthesis. The polyamines spermine, spermidine, and putrescine are low-molecular-weight aliphatic amines essential for cellular proliferation and tumor promotion. Multiple alternatively spliced transcript variants have been identified. Pseudogenes of this gene are found on chromosomes 5, 6, 10, X and Y. [provided by RefSeq, Dec 2013], | hsa00270:Cysteine and methionine metabolism,hsa00330:Arginine and proline metabolism,hsa01100:Metabolic pathways, |
| 216 | aldehyde dehydrogenase 1 family member A1(ALDH1A1) | The protein encoded by this gene belongs to the aldehyde dehydrogenase family. Aldehyde dehydrogenase is the next enzyme after alcohol dehydrogenase in the major pathway of alcohol metabolism. There are two major aldehyde dehydrogenase isozymes in the liver, cytosolic and mitochondrial, which are encoded by distinct genes, and can be distinguished by their electrophoretic mobility, kinetic properties, and subcellular localization. This gene encodes the cytosolic isozyme. Studies in mice show that through its role in retinol metabolism, this gene may also be involved in the regulation of the metabolic responses to high-fat diet. [provided by RefSeq, Mar 2011], | hsa00830:Retinol metabolism,hsa01100:Metabolic pathways, |
| 88455 | ankyrin repeat domain 13A(ANKRD13A) | Enables ubiquitin-dependent protein binding activity. Involved in negative regulation of protein localization to endosome and negative regulation of receptor internalization. Located in late endosome; perinuclear region of cytoplasm; and plasma membrane. [provided by Alliance of Genome Resources, Apr 2022], |  |
| 157567 | ankyrin repeat domain 46(ANKRD46) | This gene encodes a protein containing multiple ankyrin repeats. Ankyrin domains function in protein-protein interactions in a variety of cellular processes. Alternative splicing results in multiple transcript variants. [provided by RefSeq, Jun 2012], |  |
| 302 | annexin A2(ANXA2) | This gene encodes a member of the annexin family. Members of this calcium-dependent phospholipid-binding protein family play a role in the regulation of cellular growth and in signal transduction pathways. This protein functions as an autocrine factor which heightens osteoclast formation and bone resorption. This gene has three pseudogenes located on chromosomes 4, 9 and 10, respectively. Multiple alternatively spliced transcript variants encoding different isoforms have been found for this gene. Annexin A2 expression has been found to correlate with resistance to treatment against various cancer forms. [provided by RefSeq, Dec 2019], | hsa05132:Salmonella infection, |
| 307 | annexin A4(ANXA4) | Annexin IV (ANX4) belongs to the annexin family of calcium-dependent phospholipid binding proteins. Although their functions are still not clearly defined, several members of the annexin family have been implicated in membrane-related events along exocytotic and endocytotic pathways. ANX4 has 45 to 59% identity with other members of its family and shares a similar size and exon-intron organization. Isolated from human placenta, ANX4 encodes a protein that has possible interactions with ATP, and has in vitro anticoagulant activity and also inhibits phospholipase A2 activity. ANX4 is almost exclusively expressed in epithelial cells. Several transcript variants encoding different isoforms have been found for this gene. [provided by RefSeq, Mar 2016], |  |
| 308 | annexin A5(ANXA5) | The Annexin 5 gene spans 29 kb containing 13 exons, and encodes a single transcript of approximately 1.6 kb and a protein product with a molecular weight of about 35 kDa.The protein encoded by this gene belongs to the annexin family of calcium-dependent phospholipid binding proteins some of which have been implicated in membrane-related events along exocytotic and endocytotic pathways. Annexin 5 is a phospholipase A2 and protein kinase C inhibitory protein with calcium channel activity and a potential role in cellular signal transduction, inflammation, growth and differentiation. Annexin 5 has also been described as placental anticoagulant protein I, vascular anticoagulant-alpha, endonexin II, lipocortin V, placental protein 4 and anchorin CII. Polymorphisms in this gene have been implicated in various obstetric complications. [provided by RefSeq, Dec 2019], |  |
| 309 | annexin A6(ANXA6) | Annexin VI belongs to a family of calcium-dependent membrane and phospholipid binding proteins. Several members of the annexin family have been implicated in membrane-related events along exocytotic and endocytotic pathways. The annexin VI gene is approximately 60 kbp long and contains 26 exons. It encodes a protein of about 68 kDa that consists of eight 68-amino acid repeats separated by linking sequences of variable lengths. It is highly similar to human annexins I and II sequences, each of which contain four such repeats. Annexin VI has been implicated in mediating the endosome aggregation and vesicle fusion in secreting epithelia during exocytosis. Alternatively spliced transcript variants have been described. [provided by RefSeq, Aug 2010], |  |
| 64782 | apoptosis enhancing nuclease(AEN) | Enables exonuclease activity. Involved in intrinsic apoptotic signaling pathway in response to DNA damage by p53 class mediator and response to ionizing radiation. Located in nuclear membrane; nucleolus; and nucleoplasm. [provided by Alliance of Genome Resources, Apr 2022], |  |
| 9823 | armadillo repeat containing X-linked 2(ARMCX2) | This gene encodes a protein containing a potential N-terminal transmembrane domain and multiple armadillo (arm) repeats. Proteins containing arm repeats are involved in development, maintenance of tissue integrity, and tumorigenesis. This gene is located in a cluster of related genes on chromosome X. There is a pseudogene for this gene on chromosome 7. Alternative splicing in the 5&apos; UTR results in multiple transcript variants encoding the same protein. [provided by RefSeq, Aug 2013], |  |
| 196 | aryl hydrocarbon receptor(AHR) | The protein encoded by this gene is a ligand-activated helix-loop-helix transcription factor involved in the regulation of biological responses to planar aromatic hydrocarbons. This receptor has been shown to regulate xenobiotic-metabolizing enzymes such as cytochrome P450. Before ligand binding, the encoded protein is sequestered in the cytoplasm; upon ligand binding, this protein moves to the nucleus and stimulates transcription of target genes. [provided by RefSeq, Sep 2015], | hsa04659:Th17 cell differentiation,hsa04934:Cushing syndrome,hsa05207:Chemical carcinogenesis - receptor activation,hsa05208:Chemical carcinogenesis - reactive oxygen species, |
| 51733 | beta-ureidopropionase 1(UPB1) | This gene encodes a protein that belongs to the CN hydrolase family. Beta-ureidopropionase catalyzes the last step in the pyrimidine degradation pathway. The pyrimidine bases uracil and thymine are degraded via the consecutive action of dihydropyrimidine dehydrogenase (DHPDH), dihydropyrimidinase (DHP) and beta-ureidopropionase (UP) to beta-alanine and beta-aminoisobutyric acid, respectively. UP deficiencies are associated with N-carbamyl-beta-amino aciduria and may lead to abnormalities in neurological activity. [provided by RefSeq, Jul 2008], | hsa00240:Pyrimidine metabolism,hsa00410:beta-Alanine metabolism,hsa00770:Pantothenate and CoA biosynthesis,hsa00983:Drug metabolism - other enzymes,hsa01100:Metabolic pathways, |
| 683 | bone marrow stromal cell antigen 1(BST1) | Bone marrow stromal cell antigen-1 is a stromal cell line-derived glycosylphosphatidylinositol-anchored molecule that facilitates pre-B-cell growth. The deduced amino acid sequence exhibits 33% similarity with CD38. BST1 expression is enhanced in bone marrow stromal cell lines derived from patients with rheumatoid arthritis. The polyclonal B-cell abnormalities in rheumatoid arthritis may be, at least in part, attributed to BST1 overexpression in the stromal cell population. [provided by RefSeq, Jul 2008], | hsa00760:Nicotinate and nicotinamide metabolism,hsa01100:Metabolic pathways,hsa04970:Salivary secretion,hsa04972:Pancreatic secretion, |
| 10409 | brain abundant membrane attached signal protein 1(BASP1) | This gene encodes a membrane bound protein with several transient phosphorylation sites and PEST motifs. Conservation of proteins with PEST sequences among different species supports their functional significance. PEST sequences typically occur in proteins with high turnover rates. Immunological characteristics of this protein are species specific. This protein also undergoes N-terminal myristoylation. Alternative splicing results in multiple transcript variants that encode the same protein. [provided by RefSeq, Oct 2012], |  |
| 11177 | bromodomain adjacent to zinc finger domain 1A(BAZ1A) | The BAZ1A gene encodes the accessory subunit of the ATP-dependent chromatin assembly factor (ACF), a member of the ISWI (&apos;imitation switch&apos;) family of chromatin remodeling complexes (summarized by Racki et al., 2009 [PubMed 20033039]).[supplied by OMIM, Apr 2010], |  |
| 8573 | calcium/calmodulin dependent serine protein kinase(CASK) | This gene encodes a calcium/calmodulin-dependent serine protein kinase. The encoded protein is a MAGUK (membrane-associated guanylate kinase) protein family member. These proteins are scaffold proteins and the encoded protein is located at synapses in the brain. Mutations in this gene are associated with FG syndrome 4, intellectual disability and microcephaly with pontine and cerebellar hypoplasia, and a form of X-linked intellectual disability. Multiple transcript variants encoding different isoforms have been found for this gene. [provided by RefSeq, Jul 2017], |  |
| 51363 | carbohydrate sulfotransferase 15(CHST15) | Chondroitin sulfate (CS) is a glycosaminoglycan which is an important structural component of the extracellular matrix and which links to proteins to form proteoglycans. Chondroitin sulfate E (CS-E) is an isomer of chondroitin sulfate in which the C-4 and C-6 hydroxyl groups are sulfated. This gene encodes a type II transmembrane glycoprotein that acts as a sulfotransferase to transfer sulfate to the C-6 hydroxal group of chondroitin sulfate. This gene has also been identified as being co-expressed with RAG1 in B-cells and as potentially acting as a B-cell surface signaling receptor. Alternative splicing results in multiple transcript variants encoding distinct isoforms. [provided by RefSeq, Jul 2012], | hsa00532:Glycosaminoglycan biosynthesis - chondroitin sulfate / dermatan sulfate, |
| 10404 | carboxypeptidase Q(CPQ) | This gene encodes a metallopeptidase that belongs to the peptidase M28 family. The encoded protein may catalyze the cleavage of dipeptides with unsubstituted terminals into amino acids. [provided by RefSeq, Jul 2013], |  |
| 837 | caspase 4(CASP4) | This gene encodes a protein that is a member of the cysteine-aspartic acid protease (caspase) family. Sequential activation of caspases plays a central role in the execution-phase of cell apoptosis. Caspases exist as inactive proenzymes composed of a prodomain and a large and small protease subunit. Activation of caspases requires proteolytic processing at conserved internal aspartic residues to generate a heterodimeric enzyme consisting of the large and small subunits. This caspase is able to cleave and activate its own precursor protein, as well as caspase 1 precursor. When overexpressed, this gene induces cell apoptosis. Alternative splicing results in transcript variants encoding distinct isoforms. [provided by RefSeq, Jul 2008], | hsa04613:Neutrophil extracellular trap formation,hsa04621:NOD-like receptor signaling pathway,hsa05130:Pathogenic Escherichia coli infection,hsa05131:Shigellosis,hsa05132:Salmonella infection, |
| 1520 | cathepsin S(CTSS) | The preproprotein encoded by this gene, a member of the peptidase C1 family, is a lysosomal cysteine proteinase that participates in the degradation of antigenic proteins to peptides for presentation on MHC class II molecules. The mature protein cleaves the invariant chain of MHC class II molecules in endolysosomal compartments and enables the formation of antigen-MHC class II complexes and the proper display of extracellular antigenic peptides by MHC-II. The mature protein also functions as an elastase over a broad pH range. When secreted from cells, this protein can remodel components of the extracellular matrix such as elastin, collagen, and fibronectin. This gene is implicated in the pathology of many inflammatory and autoimmune diseases and, given its elastase activity, plays a significant role in some pulmonary diseases. Alternatively spliced transcript variants encoding distinct isoforms have been found for this gene. [provided by RefSeq, May 2020], | hsa04142:Lysosome,hsa04145:Phagosome,hsa04210:Apoptosis,hsa04612:Antigen processing and presentation,hsa05152:Tuberculosis, |
| 55038 | cell division cycle associated 4(CDCA4) | This gene encodes a protein that belongs to the E2F family of transcription factors. This protein regulates E2F-dependent transcriptional activation and cell proliferation, mainly through the E2F/retinoblastoma protein pathway. It also functions in the regulation of JUN oncogene expression. This protein shows distinctive nuclear-mitotic apparatus distribution, it is involved in spindle organization from prometaphase, and may also play a role as a midzone factor involved in chromosome segregation or cytokinesis. Two alternatively spliced transcript variants encoding the same protein have been noted for this gene. Two pseudogenes have also been identified on chromosome 1. [provided by RefSeq, May 2014], |  |
| 9662 | centrosomal protein 135(CEP135) | This gene encodes a centrosomal protein, which acts as a scaffolding protein during early centriole biogenesis, and is also required for centriole-centriole cohesion during interphase. Mutations in this gene are associated with autosomal recessive primary microcephaly-8. [provided by RefSeq, Jun 2012], |  |
| 1193 | chloride intracellular channel 2(CLIC2) | This gene encodes a chloride intracellular channel protein. Chloride channels are a diverse group of proteins that regulate fundamental cellular processes including stabilization of cell membrane potential, transepithelial transport, maintenance of intracellular pH, and regulation of cell volume. This protein plays a role in inhibiting the function of ryanodine receptor 2. A mutation in this gene is the cause of an X-linked form of cognitive disability. [provided by RefSeq, Jul 2017], |  |
| 1434 | chromosome segregation 1 like(CSE1L) | Proteins that carry a nuclear localization signal (NLS) are transported into the nucleus by the importin-alpha/beta heterodimer. Importin-alpha binds the NLS, while importin-beta mediates translocation through the nuclear pore complex. After translocation, RanGTP binds importin-beta and displaces importin-alpha. Importin-alpha must then be returned to the cytoplasm, leaving the NLS protein behind. The protein encoded by this gene binds strongly to NLS-free importin-alpha, and this binding is released in the cytoplasm by the combined action of RANBP1 and RANGAP1. In addition, the encoded protein may play a role both in apoptosis and in cell proliferation. Alternatively spliced transcript variants have been found for this gene. [provided by RefSeq, Jan 2012], | hsa03013:Nucleocytoplasmic transport,hsa05132:Salmonella infection, |
| 2153 | coagulation factor V(F5) | This gene encodes an essential cofactor of the blood coagulation cascade. This factor circulates in plasma, and is converted to the active form by the release of the activation peptide by thrombin during coagulation. This generates a heavy chain and a light chain which are held together by calcium ions. The activated protein is a cofactor that participates with activated coagulation factor X to activate prothrombin to thrombin. Defects in this gene result in either an autosomal recessive hemorrhagic diathesis or an autosomal dominant form of thrombophilia, which is known as activated protein C resistance. [provided by RefSeq, Oct 2008], | hsa04610:Complement and coagulation cascades, |
| 93058 | coenzyme Q10A(COQ10A) | Predicted to enable ubiquinone binding activity. Predicted to be involved in cellular respiration and ubiquinone biosynthetic process. Predicted to be located in mitochondrial inner membrane. Predicted to be active in mitochondrion. [provided by Alliance of Genome Resources, Apr 2022], |  |
| 152137 | coiled-coil domain containing 50(CCDC50) | This gene encodes a soluble, cytoplasmic, tyrosine-phosphorylated protein with multiple ubiquitin-interacting domains. Mutations in this gene cause nonsyndromic, postlingual, progressive sensorineural DFNA44 hearing loss. In mouse, the protein is expressed in the inner ear during development and postnatal maturation and associates with microtubule-based structures. This protein may also function as a negative regulator of NF-kB signaling and as an effector of epidermal growth factor (EGF)-mediated cell signaling. Alternative splicing results in multiple transcript variants encoding distinct isoforms. [provided by RefSeq, Oct 2008], |  |
| 51279 | complement C1r subcomponent like(C1RL) | Predicted to enable serine-type endopeptidase activity. Predicted to be involved in zymogen activation. Located in extracellular exosome. [provided by Alliance of Genome Resources, Apr 2022], |  |
| 1378 | complement C3b/C4b receptor 1 (Knops blood group)(CR1) | This gene is a member of the receptors of complement activation (RCA) family and is located in the &apos;cluster RCA&apos; region of chromosome 1. The genome is polymorphic at this locus with allele-specific splice variants encoding different isoforms, based on the presence/absence of long homologous repeats (LHRs). The gene encodes a monomeric single-pass type I membrane glycoprotein found on erythrocytes, leukocytes, glomerular podocytes, and splenic follicular dendritic cells. The Knops blood group system is a system of antigens located on this protein. The protein mediates cellular binding to particles and immune complexes that have activated complement. Decreases in expression of this protein and/or mutations in this gene have been associated with gallbladder carcinomas, mesangiocapillary glomerulonephritis, systemic lupus erythematosus, sarcoidosis and Alzheimer&apos;s disease. Mutations in this gene have also been associated with a reduction in Plasmodium falciparum rosetting, conferring protection against severe malaria. [provided by RefSeq, May 2020], | hsa04610:Complement and coagulation cascades,hsa04613:Neutrophil extracellular trap formation,hsa04640:Hematopoietic cell lineage,hsa05134:Legionellosis,hsa05140:Leishmaniasis,hsa05144:Malaria,hsa05152:Tuberculosis, |
| 5199 | complement factor properdin(CFP) | This gene encodes a plasma glycoprotein that positively regulates the alternative complement pathway of the innate immune system. This protein binds to many microbial surfaces and apoptotic cells and stabilizes the C3- and C5-convertase enzyme complexes in a feedback loop that ultimately leads to formation of the membrane attack complex and lysis of the target cell. Mutations in this gene result in two forms of properdin deficiency, which results in high susceptibility to meningococcal infections. Multiple alternatively spliced variants, encoding the same protein, have been identified.[provided by RefSeq, Feb 2009], | hsa05168:Herpes simplex virus 1 infection, |
| 865 | core-binding factor subunit beta(CBFB) | The protein encoded by this gene is the beta subunit of a heterodimeric core-binding transcription factor belonging to the PEBP2/CBF transcription factor family which master-regulates a host of genes specific to hematopoiesis (e.g., RUNX1) and osteogenesis (e.g., RUNX2). The beta subunit is a non-DNA binding regulatory subunit; it allosterically enhances DNA binding by alpha subunit as the complex binds to the core site of various enhancers and promoters, including murine leukemia virus, polyomavirus enhancer, T-cell receptor enhancers and GM-CSF promoters. Alternative splicing generates two mRNA variants, each encoding a distinct carboxyl terminus. In some cases, a pericentric inversion of chromosome 16 [inv(16)(p13q22)] produces a chimeric transcript consisting of the N terminus of core-binding factor beta in a fusion with the C-terminal portion of the smooth muscle myosin heavy chain 11. This chromosomal rearrangement is associated with acute myeloid leukemia of the M4Eo subtype. Two transcript variants encoding different isoforms have been found for this gene. [provided by RefSeq, Jul 2008], |  |
| 894 | cyclin D2(CCND2) | The protein encoded by this gene belongs to the highly conserved cyclin family, whose members are characterized by a dramatic periodicity in protein abundance through the cell cycle. Cyclins function as regulators of CDK kinases. Different cyclins exhibit distinct expression and degradation patterns which contribute to the temporal coordination of each mitotic event. This cyclin forms a complex with CDK4 or CDK6 and functions as a regulatory subunit of the complex, whose activity is required for cell cycle G1/S transition. This protein has been shown to interact with and be involved in the phosphorylation of tumor suppressor protein Rb. Knockout studies of the homologous gene in mouse suggest the essential roles of this gene in ovarian granulosa and germ cell proliferation. High level expression of this gene was observed in ovarian and testicular tumors. Mutations in this gene are associated with megalencephaly-polymicrogyria-polydactyly-hydrocephalus syndrome 3 (MPPH3). [provided by RefSeq, Sep 2014], | hsa04068:FoxO signaling pathway,hsa04110:Cell cycle,hsa04115:p53 signaling pathway,hsa04151:PI3K-Akt signaling pathway,hsa04218:Cellular senescence,hsa04310:Wnt signaling pathway,hsa04340:Hedgehog signaling pathway,hsa04390:Hippo signaling pathway,hsa04510:Focal adhesion,hsa04630:JAK-STAT signaling pathway,hsa04917:Prolactin signaling pathway,hsa05162:Measles,hsa05165:Human papillomavirus infection,hsa05166:Human T-cell leukemia virus 1 infection,hsa05169:Epstein-Barr virus infection,hsa05200:Pathways in cancer,hsa05202:Transcriptional misregulation in cancer,hsa05203:Viral carcinogenesis,hsa05206:MicroRNAs in cancer, |
| 1019 | cyclin dependent kinase 4(CDK4) | The protein encoded by this gene is a member of the Ser/Thr protein kinase family. This protein is highly similar to the gene products of S. cerevisiae cdc28 and S. pombe cdc2. It is a catalytic subunit of the protein kinase complex that is important for cell cycle G1 phase progression. The activity of this kinase is restricted to the G1-S phase, which is controlled by the regulatory subunits D-type cyclins and CDK inhibitor p16(INK4a). This kinase was shown to be responsible for the phosphorylation of retinoblastoma gene product (Rb). Mutations in this gene as well as in its related proteins including D-type cyclins, p16(INK4a) and Rb were all found to be associated with tumorigenesis of a variety of cancers. Multiple polyadenylation sites of this gene have been reported. [provided by RefSeq, Jul 2008], | hsa01522:Endocrine resistance,hsa04110:Cell cycle,hsa04115:p53 signaling pathway,hsa04151:PI3K-Akt signaling pathway,hsa04218:Cellular senescence,hsa04530:Tight junction,hsa04660:T cell receptor signaling pathway,hsa04933:AGE-RAGE signaling pathway in diabetic complications,hsa04934:Cushing syndrome,hsa05160:Hepatitis C,hsa05162:Measles,hsa05163:Human cytomegalovirus infection,hsa05164:Influenza A,hsa05165:Human papillomavirus infection,hsa05166:Human T-cell leukemia virus 1 infection,hsa05167:Kaposi sarcoma-associated herpesvirus infection,hsa05169:Epstein-Barr virus infection,hsa05200:Pathways in cancer,hsa05203:Viral carcinogenesis,hsa05212:Pancreatic cancer,hsa05214:Glioma,hsa05218:Melanoma,hsa05219:Bladder cancer,hsa05220:Chronic myeloid leukemia,hsa05222:Small cell lung cancer,hsa05223:Non-small cell lung cancer,hsa05224:Breast cancer,hsa05225:Hepatocellular carcinoma, |
| 79901 | cytochrome b reductase 1(CYBRD1) | This gene is a member of the cytochrome b(561) family that encodes an iron-regulated protein. It highly expressed in the duodenal brush border membrane. It has ferric reductase activity and is believed to play a physiological role in dietary iron absorption. [provided by RefSeq, Jul 2008], | hsa04978:Mineral absorption, |
| 51167 | cytochrome b5 reductase 4(CYB5R4) | NCB5OR is a flavohemoprotein that contains functional domains found in both cytochrome b5 (CYB5A; MIM 613218) and CYB5 reductase (CYB5R3; MIM 613213) (Zhu et al., 1999 [PubMed 10611283]).[supplied by OMIM, Jan 2010], | hsa00520:Amino sugar and nucleotide sugar metabolism, |
| 1528 | cytochrome b5 type A(CYB5A) | The protein encoded by this gene is a membrane-bound cytochrome that reduces ferric hemoglobin (methemoglobin) to ferrous hemoglobin, which is required for stearyl-CoA-desaturase activity. Defects in this gene are a cause of type IV hereditary methemoglobinemia. Three transcript variants encoding different isoforms have been found for this gene. [provided by RefSeq, Jun 2010], |  |
| 9267 | cytohesin 1(CYTH1) | The protein encoded by this gene is a member of the PSCD family. Members of this family have identical structural organization that consists of an N-terminal coiled-coil motif, a central Sec7 domain, and a C-terminal pleckstrin homology (PH) domain. The coiled-coil motif is involved in homodimerization, the Sec7 domain contains guanine-nucleotide exchange protein activity, and the PH domain interacts with phospholipids and is responsible for association of PSCDs with membranes. Members of this family appear to mediate the regulation of protein sorting and membrane trafficking. This gene is highly expressed in natural killer and peripheral T cells, and regulates the adhesiveness of integrins at the plasma membrane of lymphocytes. A pseudogene of this gene has been defined on the X chromosome. Alternative splicing results in multiple transcript variants. [provided by RefSeq, May 2014], | hsa04072:Phospholipase D signaling pathway,hsa04144:Endocytosis,hsa05130:Pathogenic Escherichia coli infection,hsa05131:Shigellosis,hsa05132:Salmonella infection, |
| 51379 | cytokine receptor like factor 3(CRLF3) | This gene encodes a cytokine receptor-like factor that may negatively regulate cell cycle progression at the G0/G1 phase. Studies of the related rat protein suggest that it may regulate neuronal morphology and synaptic vesicle biogenesis. This gene is one of several genes located in the neurofibromatosis type I tumor suppressor region on the q arm of chromosome 17, a region that is subject to microdeletions, duplications, chromosomal breaks and rearrangements. Alternative splicing of this gene results in multiple transcript variants. Related pseudogenes have been identified on chromosomes 2 and 5. [provided by RefSeq, Aug 2012], |  |
| 26999 | cytoplasmic FMR1 interacting protein 2(CYFIP2) | Predicted to enable small GTPase binding activity. Involved in activation of cysteine-type endopeptidase activity; apoptotic process; and cell-cell adhesion. Located in perinuclear region of cytoplasm and synapse. Part of SCAR complex. Implicated in developmental and epileptic encephalopathy 65. [provided by Alliance of Genome Resources, Apr 2022], | hsa04810:Regulation of actin cytoskeleton,hsa05130:Pathogenic Escherichia coli infection,hsa05132:Salmonella infection, |
| 23122 | cytoplasmic linker associated protein 2(CLASP2) | Enables cytoskeletal protein binding activity; dystroglycan binding activity; and protein tyrosine kinase binding activity. Involved in several processes, including microtubule cytoskeleton organization; positive regulation of extracellular matrix organization; and regulation of supramolecular fiber organization. Located in several cellular components, including basal cortex; cortical microtubule plus-end; and ruffle membrane. Colocalizes with focal adhesion; kinetochore; and microtubule cytoskeleton. [provided by Alliance of Genome Resources, Apr 2022], |  |
| 55619 | dedicator of cytokinesis 10(DOCK10) | This gene encodes a member of the dedicator of cytokinesis protein family. Members of this family are guanosine nucleotide exchange factors for Rho GTPases and defined by the presence of conserved DOCK-homology regions. The encoded protein belongs to the D (or Zizimin) subfamily of DOCK proteins, which also contain an N-terminal pleckstrin homology domain. Alternatively spliced transcript variants that encode different isoforms have been described. [provided by RefSeq, Mar 2014], |  |
| 55526 | dehydrogenase E1 and transketolase domain containing 1(DHTKD1) | This gene encodes a component of a mitochondrial 2-oxoglutarate-dehydrogenase-complex-like protein involved in the degradation pathways of several amino acids, including lysine. Mutations in this gene are associated with 2-aminoadipic 2-oxoadipic aciduria and Charcot-Marie-Tooth Disease Type 2Q. [provided by RefSeq, May 2013], | hsa00310:Lysine degradation,hsa00380:Tryptophan metabolism,hsa01100:Metabolic pathways, |
| 51071 | deoxyribose-phosphate aldolase(DERA) | Enables deoxyribose-phosphate aldolase activity. Involved in deoxyribonucleoside catabolic process. Located in nucleoplasm. [provided by Alliance of Genome Resources, Apr 2022], | hsa00030:Pentose phosphate pathway,hsa01100:Metabolic pathways, |
| 11034 | destrin, actin depolymerizing factor(DSTN) | The product of this gene belongs to the actin-binding proteins ADF family. This family of proteins is responsible for enhancing the turnover rate of actin in vivo. This gene encodes the actin depolymerizing protein that severs actin filaments (F-actin) and binds to actin monomers (G-actin). Two transcript variants encoding distinct isoforms have been identified for this gene. [provided by RefSeq, Jul 2008], |  |
| 1808 | dihydropyrimidinase like 2(DPYSL2) | This gene encodes a member of the collapsin response mediator protein family. Collapsin response mediator proteins form homo- and hetero-tetramers and facilitate neuron guidance, growth and polarity. The encoded protein promotes microtubule assembly and is required for Sema3A-mediated growth cone collapse, and also plays a role in synaptic signaling through interactions with calcium channels. This gene has been implicated in multiple neurological disorders, and hyperphosphorylation of the encoded protein may play a key role in the development of Alzheimer&apos;s disease. Alternatively spliced transcript variants encoding multiple isoforms have been observed for this gene. [provided by RefSeq, Sep 2011], | hsa04360:Axon guidance, |
| 79930 | docking protein 3(DOK3) | Predicted to be involved in Ras protein signal transduction and transmembrane receptor protein tyrosine kinase signaling pathway. Predicted to be located in ficolin-1-rich granule membrane and plasma membrane. Predicted to be active in cytoplasm. [provided by Alliance of Genome Resources, Apr 2022], |  |
| 8655 | dynein light chain LC8-type 1(DYNLL1) | Cytoplasmic dyneins are large enzyme complexes with a molecular mass of about 1,200 kD. They contain two force-producing heads formed primarily from dynein heavy chains, and stalks linking the heads to a basal domain, which contains a varying number of accessory intermediate chains. The complex is involved in intracellular transport and motility. The protein described in this record is a light chain and exists as part of this complex but also physically interacts with and inhibits the activity of neuronal nitric oxide synthase. Binding of this protein destabilizes the neuronal nitric oxide synthase dimer, a conformation necessary for activity, and it may regulate numerous biologic processes through its effects on nitric oxide synthase activity. Alternate transcriptional splice variants have been characterized. [provided by RefSeq, Jul 2008], | hsa04962:Vasopressin-regulated water reabsorption,hsa05132:Salmonella infection, |
| 83658 | dynein light chain roadblock-type 1(DYNLRB1) | This gene is a member of the roadblock dynein light chain family. The encoded cytoplasmic protein is capable of binding intermediate chain proteins, interacts with transforming growth factor-beta, and has been implicated in the regulation of actin modulating proteins. Upregulation of this gene has been associated with hepatocellular carcinomas, suggesting that this gene may be involved in tumor progression. Alternative splicing results in multiple transcript variants. Pseudogenes of this gene have been defined on chromosomes 12 and 18. [provided by RefSeq, Aug 2013], | hsa05132:Salmonella infection, |
| 2123 | ecotropic viral integration site 2A(EVI2A) | Predicted to be integral component of membrane. [provided by Alliance of Genome Resources, Apr 2022], |  |
| 2124 | ecotropic viral integration site 2B(EVI2B) | Involved in positive regulation of granulocyte differentiation. Predicted to be integral component of plasma membrane. [provided by Alliance of Genome Resources, Apr 2022], |  |
| 79956 | endoplasmic reticulum metallopeptidase 1(ERMP1) | Predicted to enable metal ion binding activity and metalloexopeptidase activity. Involved in cellular response to oxidative stress. Acts upstream of or within endoplasmic reticulum unfolded protein response. Located in membrane. [provided by Alliance of Genome Resources, Apr 2022], |  |
| 1632 | enoyl-CoA delta isomerase 1(ECI1) | This gene encodes a member of the hydratase/isomerase superfamily. The protein encoded is a key mitochondrial enzyme involved in beta-oxidation of unsaturated fatty acids. It catalyzes the transformation of 3-cis and 3-trans-enoyl-CoA esters arising during the stepwise degradation of cis-, mono-, and polyunsaturated fatty acids to the 2-trans-enoyl-CoA intermediates. Alternatively spliced transcript variants have been described. [provided by RefSeq, May 2010], | hsa00071:Fatty acid degradation, |
| 2060 | epidermal growth factor receptor pathway substrate 15(EPS15) | This gene encodes a protein that is part of the EGFR pathway. The protein is present at clatherin-coated pits and is involved in receptor-mediated endocytosis of EGF. Notably, this gene is rearranged with the HRX/ALL/MLL gene in acute myelogeneous leukemias. Alternative splicing results in multiple transcript variants encoding distinct isoforms. [provided by RefSeq, May 2009], | hsa04144:Endocytosis, |
| 2135 | exostosin like glycosyltransferase 2(EXTL2) | Enables alpha-1,4-N-acetylgalactosaminyltransferase activity and glucuronyl-galactosyl-proteoglycan 4-alpha-N-acetylglucosaminyltransferase activity. Involved in N-acetylglucosamine metabolic process and UDP-N-acetylgalactosamine metabolic process. Located in cytosol; endoplasmic reticulum; and nucleoplasm. [provided by Alliance of Genome Resources, Apr 2022], | hsa00534:Glycosaminoglycan biosynthesis - heparan sulfate / heparin,hsa01100:Metabolic pathways, |
| 23214 | exportin 6(XPO6) | The protein encoded by this gene is a member of the importin-beta family. Members of this family are regulated by the GTPase Ran to mediate transport of cargo across the nuclear envelope. This protein has been shown to mediate nuclear export of profilin-actin complexes. A pseudogene of this gene is located on the long arm of chromosome 14. Alternative splicing results in multiple transcript variants that encode different protein isoforms. [provided by RefSeq, Aug 2012], | hsa03013:Nucleocytoplasmic transport, |
| 23344 | extended synaptotagmin 1(ESYT1) | Enables identical protein binding activity. Predicted to be involved in endoplasmic reticulum-plasma membrane tethering and lipid transport. Located in endoplasmic reticulum. Is integral component of endoplasmic reticulum membrane. [provided by Alliance of Genome Resources, Apr 2022], |  |
| 83989 | family with sequence similarity 172 member A(FAM172A) | Predicted to contribute to siRNA binding activity. Predicted to be involved in heterochromatin assembly by small RNA; neural crest cell development; and regulation of alternative mRNA splicing, via spliceosome. Located in endoplasmic reticulum. [provided by Alliance of Genome Resources, Apr 2022], |  |
| 80256 | family with sequence similarity 214 member B(FAM214B) | Located in nucleus. [provided by Alliance of Genome Resources, Apr 2022], |  |
| 10875 | fibrinogen like 2(FGL2) | The protein encoded by this gene is a secreted protein that is similar to the beta- and gamma-chains of fibrinogen. The carboxyl-terminus of the encoded protein consists of the fibrinogen-related domains (FRED). The encoded protein forms a tetrameric complex which is stabilized by interchain disulfide bonds. This protein may play a role in physiologic functions at mucosal sites. [provided by RefSeq, Jul 2008], |  |
| 2319 | flotillin 2(FLOT2) | Caveolae are small domains on the inner cell membrane involved in vesicular trafficking and signal transduction. This gene encodes a caveolae-associated, integral membrane protein, which is thought to function in neuronal signaling. [provided by RefSeq, Jul 2008], | hsa04910:Insulin signaling pathway, |
| 3344 | forkhead box N2(FOXN2) | This gene encodes a forkhead domain binding protein and may function in the transcriptional regulation of the human T-cell leukemia virus long terminal repeat. [provided by RefSeq, Jul 2008], |  |
| 2357 | formyl peptide receptor 1(FPR1) | This gene encodes a G protein-coupled receptor of mammalian phagocytic cells that is a member of the G-protein coupled receptor 1 family. The protein mediates the response of phagocytic cells to invasion of the host by microorganisms and is important in host defense and inflammation.[provided by RefSeq, Jul 2010], | hsa04015:Rap1 signaling pathway,hsa04080:Neuroactive ligand-receptor interaction,hsa04613:Neutrophil extracellular trap formation,hsa05150:Staphylococcus aureus infection, |
| 3956 | galectin 1(LGALS1) | The galectins are a family of beta-galactoside-binding proteins implicated in modulating cell-cell and cell-matrix interactions. This gene product may act as an autocrine negative growth factor that regulates cell proliferation. [provided by RefSeq, Jul 2008], |  |
| 3959 | galectin 3 binding protein(LGALS3BP) | The galectins are a family of beta-galactoside-binding proteins implicated in modulating cell-cell and cell-matrix interactions. LGALS3BP has been found elevated in the serum of patients with cancer and in those infected by the human immunodeficiency virus (HIV). It appears to be implicated in immune response associated with natural killer (NK) and lymphokine-activated killer (LAK) cell cytotoxicity. Using fluorescence in situ hybridization the full length 90K cDNA has been localized to chromosome 17q25. The native protein binds specifically to a human macrophage-associated lectin known as Mac-2 and also binds galectin 1. [provided by RefSeq, Jul 2008], |  |
| 3958 | galectin 3(LGALS3) | This gene encodes a member of the galectin family of carbohydrate binding proteins. Members of this protein family have an affinity for beta-galactosides. The encoded protein is characterized by an N-terminal proline-rich tandem repeat domain and a single C-terminal carbohydrate recognition domain. This protein can self-associate through the N-terminal domain allowing it to bind to multivalent saccharide ligands. This protein localizes to the extracellular matrix, the cytoplasm and the nucleus. This protein plays a role in numerous cellular functions including apoptosis, innate immunity, cell adhesion and T-cell regulation. The protein exhibits antimicrobial activity against bacteria and fungi. Alternate splicing results in multiple transcript variants.[provided by RefSeq, Oct 2014], |  |
| 2960 | general transcription factor IIE subunit 1(GTF2E1) | Enables RNA polymerase II general transcription initiation factor activity. Involved in transcription by RNA polymerase II. Located in cytosol and nucleoplasm. Part of transcription factor TFIID complex and transcription preinitiation complex. [provided by Alliance of Genome Resources, Apr 2022], | hsa03022:Basal transcription factors,hsa05203:Viral carcinogenesis, |
| 2746 | glutamate dehydrogenase 1(GLUD1) | This gene encodes glutamate dehydrogenase, which is a mitochondrial matrix enzyme that catalyzes the oxidative deamination of glutamate to alpha-ketoglutarate and ammonia. This enzyme has an important role in regulating amino acid-induced insulin secretion. It is allosterically activated by ADP and inhibited by GTP and ATP. Activating mutations in this gene are a common cause of congenital hyperinsulinism. Alternative splicing of this gene results in multiple transcript variants. The related glutamate dehydrogenase 2 gene on the human X-chromosome originated from this gene via retrotransposition and encodes a soluble form of glutamate dehydrogenase. Related pseudogenes have been identified on chromosomes 10, 18 and X. [provided by RefSeq, Jan 2016], | hsa00220:Arginine biosynthesis,hsa00250:Alanine, aspartate and glutamate metabolism,hsa00910:Nitrogen metabolism,hsa01100:Metabolic pathways,hsa01200:Carbon metabolism,hsa04217:Necroptosis,hsa04964:Proximal tubule bicarbonate reclamation, |
| 2730 | glutamate-cysteine ligase modifier subunit(GCLM) | Glutamate-cysteine ligase, also known as gamma-glutamylcysteine synthetase, is the first rate limiting enzyme of glutathione synthesis. The enzyme consists of two subunits, a heavy catalytic subunit and a light regulatory subunit. Gamma glutamylcysteine synthetase deficiency has been implicated in some forms of hemolytic anemia. Alternative splicing results in multiple transcript variants encoding different isoforms. [provided by RefSeq, Apr 2015], | hsa00270:Cysteine and methionine metabolism,hsa00480:Glutathione metabolism,hsa01100:Metabolic pathways,hsa01240:Biosynthesis of cofactors,hsa04216:Ferroptosis, |
| 25797 | glutaminyl-peptide cyclotransferase(QPCT) | This gene encodes human pituitary glutaminyl cyclase, which is responsible for the presence of pyroglutamyl residues in many neuroendocrine peptides. The amino acid sequence of this enzyme is 86% identical to that of bovine glutaminyl cyclase. [provided by RefSeq, Jul 2008], |  |
| 2745 | glutaredoxin(GLRX) | This gene encodes a member of the glutaredoxin family. The encoded protein is a cytoplasmic enzyme catalyzing the reversible reduction of glutathione-protein mixed disulfides. This enzyme highly contributes to the antioxidant defense system. It is crucial for several signalling pathways by controlling the S-glutathionylation status of signalling mediators. It is involved in beta-amyloid toxicity and Alzheimer&apos;s disease. Multiple alternatively spliced transcript variants encoding the same protein have been identified. [provided by RefSeq, Aug 2011], |  |
| 23171 | glycerol-3-phosphate dehydrogenase 1 like(GPD1L) | The protein encoded by this gene catalyzes the conversion of sn-glycerol 3-phosphate to glycerone phosphate. The encoded protein is found in the cytoplasm, associated with the plasma membrane, where it binds the sodium channel, voltage-gated, type V, alpha subunit (SCN5A). Defects in this gene are a cause of Brugada syndrome type 2 (BRS2) as well as sudden infant death syndrome (SIDS). [provided by RefSeq, Jul 2010], | hsa00564:Glycerophospholipid metabolism, |
| 51573 | glycerophosphodiester phosphodiesterase 1(GDE1) | Predicted to enable glycerophosphodiester phosphodiesterase activity; glycerophosphoinositol glycerophosphodiesterase activity; and lysophospholipase activity. Predicted to be involved in N-acylethanolamine metabolic process; ethanolamine metabolic process; and phospholipid metabolic process. Predicted to be located in plasma membrane. [provided by Alliance of Genome Resources, Apr 2022], |  |
| 5836 | glycogen phosphorylase L(PYGL) | This gene encodes a homodimeric protein that catalyses the cleavage of alpha-1,4-glucosidic bonds to release glucose-1-phosphate from liver glycogen stores. This protein switches from inactive phosphorylase B to active phosphorylase A by phosphorylation of serine residue 15. Activity of this enzyme is further regulated by multiple allosteric effectors and hormonal controls. Humans have three glycogen phosphorylase genes that encode distinct isozymes that are primarily expressed in liver, brain and muscle, respectively. The liver isozyme serves the glycemic demands of the body in general while the brain and muscle isozymes supply just those tissues. In glycogen storage disease type VI, also known as Hers disease, mutations in liver glycogen phosphorylase inhibit the conversion of glycogen to glucose and results in moderate hypoglycemia, mild ketosis, growth retardation and hepatomegaly. Alternative splicing results in multiple transcript variants encoding different isoforms.[provided by RefSeq, Feb 2011], | hsa00500:Starch and sucrose metabolism,hsa01100:Metabolic pathways,hsa04217:Necroptosis,hsa04910:Insulin signaling pathway,hsa04922:Glucagon signaling pathway,hsa04931:Insulin resistance, |
| 2932 | glycogen synthase kinase 3 beta(GSK3B) | The protein encoded by this gene is a serine-threonine kinase belonging to the glycogen synthase kinase subfamily. It is a negative regulator of glucose homeostasis and is involved in energy metabolism, inflammation, ER-stress, mitochondrial dysfunction, and apoptotic pathways. Defects in this gene have been associated with Parkinson disease and Alzheimer disease. [provided by RefSeq, Aug 2017], | hsa01521:EGFR tyrosine kinase inhibitor resistance,hsa04012:ErbB signaling pathway,hsa04062:Chemokine signaling pathway,hsa04110:Cell cycle,hsa04150:mTOR signaling pathway,hsa04151:PI3K-Akt signaling pathway,hsa04310:Wnt signaling pathway,hsa04340:Hedgehog signaling pathway,hsa04360:Axon guidance,hsa04390:Hippo signaling pathway,hsa04510:Focal adhesion,hsa04550:Signaling pathways regulating pluripotency of stem cells,hsa04657:IL-17 signaling pathway,hsa04660:T cell receptor signaling pathway,hsa04662:B cell receptor signaling pathway,hsa04722:Neurotrophin signaling pathway,hsa04728:Dopaminergic synapse,hsa04910:Insulin signaling pathway,hsa04916:Melanogenesis,hsa04917:Prolactin signaling pathway,hsa04919:Thyroid hormone signaling pathway,hsa04931:Insulin resistance,hsa04932:Non-alcoholic fatty liver disease,hsa04934:Cushing syndrome,hsa04935:Growth hormone synthesis, secretion and action,hsa04936:Alcoholic liver disease,hsa05010:Alzheimer disease,hsa05020:Prion disease,hsa05022:Pathways of neurodegeneration - multiple diseases,hsa05131:Shigellosis,hsa05135:Yersinia infection,hsa05160:Hepatitis C,hsa05162:Measles,hsa05163:Human cytomegalovirus infection,hsa05165:Human papillomavirus infection,hsa05167:Kaposi sarcoma-associated herpesvirus infection,hsa05200:Pathways in cancer,hsa05210:Colorectal cancer,hsa05213:Endometrial cancer,hsa05215:Prostate cancer,hsa05217:Basal cell carcinoma,hsa05224:Breast cancer,hsa05225:Hepatocellular carcinoma,hsa05226:Gastric cancer,hsa05415:Diabetic cardiomyopathy,hsa05417:Lipid and atherosclerosis, |
| 2739 | glyoxalase I(GLO1) | The enzyme encoded by this gene is responsible for the catalysis and formation of S-lactoyl-glutathione from methylglyoxal condensation and reduced glutatione. Glyoxalase I is linked to HLA and is localized to 6p21.3-p21.1, between HLA and the centromere. [provided by RefSeq, Jul 2008], | hsa00620:Pyruvate metabolism,hsa01100:Metabolic pathways, |
| 9570 | golgi SNAP receptor complex member 2(GOSR2) | This gene encodes a trafficking membrane protein which transports proteins among the medial- and trans-Golgi compartments. Due to its chromosomal location and trafficking function, this gene may be involved in familial essential hypertension. [provided by RefSeq, Mar 2016], | hsa04130:SNARE interactions in vesicular transport, |
| 25801 | grancalcin(GCA) | This gene encodes a calcium-binding protein that is abundant in neutrophils and macrophages. In the absence of divalent cation, this protein localizes to the cytosolic fraction; with magnesium alone, it partitions with the granule fraction; and in the presence of magnesium and calcium, it associates with both the granule and membrane fractions. Alternative splicing and use of alternative promoters results in multiple transcript variants. [provided by RefSeq, Aug 2016], |  |
| 9710 | granule associated Rac and RHOG effector 1(GARRE1) | Enables CCR4-NOT complex binding activity and small GTPase binding activity. Involved in Rac protein signal transduction. Located in P-body. [provided by Alliance of Genome Resources, Apr 2022], |  |
| 3310 | heat shock protein family A (Hsp70) member 6(HSPA6) | Enables enzyme binding activity; heat shock protein binding activity; and unfolded protein binding activity. Involved in cellular response to heat and protein refolding. Located in centriole and cytosol. Colocalizes with COP9 signalosome. [provided by Alliance of Genome Resources, Apr 2022], | hsa03040:Spliceosome,hsa04010:MAPK signaling pathway,hsa04141:Protein processing in endoplasmic reticulum,hsa04144:Endocytosis,hsa04213:Longevity regulating pathway - multiple species,hsa04612:Antigen processing and presentation,hsa04915:Estrogen signaling pathway,hsa05020:Prion disease,hsa05134:Legionellosis,hsa05145:Toxoplasmosis,hsa05162:Measles,hsa05417:Lipid and atherosclerosis, |
| 10870 | hematopoietic cell signal transducer(HCST) | This gene encodes a transmembrane signaling adaptor that contains a YxxM motif in its cytoplasmic domain. The encoded protein may form part of the immune recognition receptor complex with the C-type lectin-like receptor NKG2D. As part of this receptor complex, this protein may activate phosphatidylinositol 3-kinase dependent signaling pathways through its intracytoplasmic YxxM motif. This receptor complex may have a role in cell survival and proliferation by activation of NK and T cell responses. Alternative splicing results in two transcript variants encoding different isoforms. [provided by RefSeq, Jul 2008], | hsa04650:Natural killer cell mediated cytotoxicity, |
| 3059 | hematopoietic cell-specific Lyn substrate 1(HCLS1) | Enables RNA polymerase II-specific DNA-binding transcription factor binding activity and protein kinase binding activity. Involved in several processes, including positive regulation of intracellular signal transduction; positive regulation of protein phosphorylation; and regulation of transcription, DNA-templated. Located in cytosol; nucleus; and plasma membrane. Part of transcription regulator complex. [provided by Alliance of Genome Resources, Apr 2022], | hsa04530:Tight junction,hsa05100:Bacterial invasion of epithelial cells,hsa05130:Pathogenic Escherichia coli infection,hsa05131:Shigellosis,hsa05205:Proteoglycans in cancer, |
| 3181 | heterogeneous nuclear ribonucleoprotein A2/B1(HNRNPA2B1) | This gene belongs to the A/B subfamily of ubiquitously expressed heterogeneous nuclear ribonucleoproteins (hnRNPs). The hnRNPs are RNA binding proteins and they complex with heterogeneous nuclear RNA (hnRNA). These proteins are associated with pre-mRNAs in the nucleus and appear to influence pre-mRNA processing and other aspects of mRNA metabolism and transport. While all of the hnRNPs are present in the nucleus, some seem to shuttle between the nucleus and the cytoplasm. The hnRNP proteins have distinct nucleic acid binding properties. The protein encoded by this gene has two repeats of quasi-RRM domains that bind to RNAs. This gene has been described to generate two alternatively spliced transcript variants which encode different isoforms. [provided by RefSeq, Jul 2008], | hsa05014:Amyotrophic lateral sclerosis, |
| 51170 | hydroxysteroid 17-beta dehydrogenase 11(HSD17B11) | Short-chain alcohol dehydrogenases, such as HSD17B11, metabolize secondary alcohols and ketones (Brereton et al., 2001 [PubMed 11165019]).[supplied by OMIM, Jun 2009], |  |
| 84263 | hydroxysteroid dehydrogenase like 2(HSDL2) | Predicted to enable oxidoreductase activity. Located in mitochondrion and peroxisome. [provided by Alliance of Genome Resources, Apr 2022], |  |
| 10261 | immunoglobulin superfamily member 6(IGSF6) | Predicted to enable transmembrane signaling receptor activity. Predicted to be involved in cell surface receptor signaling pathway and immune response. Predicted to be integral component of plasma membrane. [provided by Alliance of Genome Resources, Apr 2022], |  |
| 4033 | inositol 1,4,5-triphosphate receptor associated 2(IRAG2) | The protein encode dby this gene is expressed in a developmentally regulated manner in lymphoid cell lines and tissues. The protein is localized to the cytoplasmic face of the endoplasmic reticulum. [provided by RefSeq, Jul 2008], |  |
| 3628 | inositol polyphosphate-1-phosphatase(INPP1) | This gene encodes the enzyme inositol polyphosphate-1-phosphatase, one of the enzymes involved in phosphatidylinositol signaling pathways. This enzyme removes the phosphate group at position 1 of the inositol ring from the polyphosphates inositol 1,4-bisphosphate and inositol 1,3,4-trisphophosphate. [provided by RefSeq, Jul 2008], | hsa00562:Inositol phosphate metabolism,hsa01100:Metabolic pathways,hsa04070:Phosphatidylinositol signaling system, |
| 3631 | inositol polyphosphate-4-phosphatase type I A(INPP4A) | This gene encodes an Mg++ independent enzyme that hydrolyzes the 4-position phosphate from the inositol ring of phosphatidylinositol 3,4-bisphosphate, inositol 1,3,4-trisphosphate, and inositol 3,4-bisphosphate. Multiple transcript variants encoding distinct isoforms have been described. [provided by RefSeq, Aug 2008], | hsa00562:Inositol phosphate metabolism,hsa01100:Metabolic pathways,hsa04070:Phosphatidylinositol signaling system, |
| 81533 | integrin alpha FG-GAP repeat containing 1(ITFG1) | Located in extracellular exosome. [provided by Alliance of Genome Resources, Apr 2022], |  |
| 3676 | integrin subunit alpha 4(ITGA4) | The gene encodes a member of the integrin alpha chain family of proteins. Integrins are heterodimeric integral membrane proteins composed of an alpha chain and a beta chain that function in cell surface adhesion and signaling. The encoded preproprotein is proteolytically processed to generate light and heavy chains that comprise the alpha 4 subunit. This subunit associates with a beta 1 or beta 7 subunit to form an integrin that may play a role in cell motility and migration. This integrin is a therapeutic target for the treatment of multiple sclerosis, Crohn&apos;s disease and inflammatory bowel disease. Alternative splicing results in multiple transcript variants. [provided by RefSeq, Oct 2015], | hsa04151:PI3K-Akt signaling pathway,hsa04510:Focal adhesion,hsa04512:ECM-receptor interaction,hsa04514:Cell adhesion molecules,hsa04640:Hematopoietic cell lineage,hsa04670:Leukocyte transendothelial migration,hsa04672:Intestinal immune network for IgA production,hsa04810:Regulation of actin cytoskeleton,hsa05135:Yersinia infection,hsa05140:Leishmaniasis,hsa05165:Human papillomavirus infection,hsa05410:Hypertrophic cardiomyopathy,hsa05412:Arrhythmogenic right ventricular cardiomyopathy,hsa05414:Dilated cardiomyopathy, |
| 3683 | integrin subunit alpha L(ITGAL) | ITGAL encodes the integrin alpha L chain. Integrins are heterodimeric integral membrane proteins composed of an alpha chain and a beta chain. This I-domain containing alpha integrin combines with the beta 2 chain (ITGB2) to form the integrin lymphocyte function-associated antigen-1 (LFA-1), which is expressed on all leukocytes. LFA-1 plays a central role in leukocyte intercellular adhesion through interactions with its ligands, ICAMs 1-3 (intercellular adhesion molecules 1 through 3), and also functions in lymphocyte costimulatory signaling. Two transcript variants encoding different isoforms have been found for this gene. [provided by RefSeq, Jul 2008], | hsa04015:Rap1 signaling pathway,hsa04514:Cell adhesion molecules,hsa04613:Neutrophil extracellular trap formation,hsa04650:Natural killer cell mediated cytotoxicity,hsa04670:Leukocyte transendothelial migration,hsa04810:Regulation of actin cytoskeleton,hsa05144:Malaria,hsa05150:Staphylococcus aureus infection,hsa05166:Human T-cell leukemia virus 1 infection,hsa05169:Epstein-Barr virus infection,hsa05323:Rheumatoid arthritis,hsa05416:Viral myocarditis, |
| 3684 | integrin subunit alpha M(ITGAM) | This gene encodes the integrin alpha M chain. Integrins are heterodimeric integral membrane proteins composed of an alpha chain and a beta chain. This I-domain containing alpha integrin combines with the beta 2 chain (ITGB2) to form a leukocyte-specific integrin referred to as macrophage receptor 1 (&apos;Mac-1&apos;), or inactivated-C3b (iC3b) receptor 3 (&apos;CR3&apos;). The alpha M beta 2 integrin is important in the adherence of neutrophils and monocytes to stimulated endothelium, and also in the phagocytosis of complement coated particles. Multiple transcript variants encoding different isoforms have been found for this gene. [provided by RefSeq, Mar 2009], | hsa04015:Rap1 signaling pathway,hsa04145:Phagosome,hsa04514:Cell adhesion molecules,hsa04610:Complement and coagulation cascades,hsa04613:Neutrophil extracellular trap formation,hsa04640:Hematopoietic cell lineage,hsa04670:Leukocyte transendothelial migration,hsa04810:Regulation of actin cytoskeleton,hsa05133:Pertussis,hsa05134:Legionellosis,hsa05140:Leishmaniasis,hsa05146:Amoebiasis,hsa05150:Staphylococcus aureus infection,hsa05152:Tuberculosis,hsa05202:Transcriptional misregulation in cancer,hsa05221:Acute myeloid leukemia, |
| 3685 | integrin subunit alpha V(ITGAV) | The product of this gene belongs to the integrin alpha chain family. Integrins are heterodimeric integral membrane proteins composed of an alpha subunit and a beta subunit that function in cell surface adhesion and signaling. The encoded preproprotein is proteolytically processed to generate light and heavy chains that comprise the alpha V subunit. This subunit associates with beta 1, beta 3, beta 5, beta 6 and beta 8 subunits. The heterodimer consisting of alpha V and beta 3 subunits is also known as the vitronectin receptor. This integrin may regulate angiogenesis and cancer progression. Alternative splicing results in multiple transcript variants. Note that the integrin alpha 5 and integrin alpha V subunits are encoded by distinct genes. [provided by RefSeq, Oct 2015], | hsa04145:Phagosome,hsa04151:PI3K-Akt signaling pathway,hsa04510:Focal adhesion,hsa04512:ECM-receptor interaction,hsa04514:Cell adhesion molecules,hsa04810:Regulation of actin cytoskeleton,hsa04919:Thyroid hormone signaling pathway,hsa05163:Human cytomegalovirus infection,hsa05165:Human papillomavirus infection,hsa05200:Pathways in cancer,hsa05205:Proteoglycans in cancer,hsa05222:Small cell lung cancer,hsa05410:Hypertrophic cardiomyopathy,hsa05412:Arrhythmogenic right ventricular cardiomyopathy,hsa05414:Dilated cardiomyopathy,hsa05418:Fluid shear stress and atherosclerosis, |
| 3687 | integrin subunit alpha X(ITGAX) | This gene encodes the integrin alpha X chain protein. Integrins are heterodimeric integral membrane proteins composed of an alpha chain and a beta chain. This protein combines with the beta 2 chain (ITGB2) to form a leukocyte-specific integrin referred to as inactivated-C3b (iC3b) receptor 4 (CR4). The alpha X beta 2 complex seems to overlap the properties of the alpha M beta 2 integrin in the adherence of neutrophils and monocytes to stimulated endothelium cells, and in the phagocytosis of complement coated particles. Two transcript variants encoding different isoforms have been found for this gene. [provided by RefSeq, Nov 2013], | hsa04610:Complement and coagulation cascades,hsa04810:Regulation of actin cytoskeleton,hsa05152:Tuberculosis, |
| 26034 | interaction protein for cytohesin exchange factors 1(IPCEF1) | Predicted to enable peroxidase activity. Predicted to be involved in response to oxidative stress. Predicted to be located in cytosol and plasma membrane. [provided by Alliance of Genome Resources, Apr 2022], |  |
| 3385 | intercellular adhesion molecule 3(ICAM3) | The protein encoded by this gene is a member of the intercellular adhesion molecule (ICAM) family. All ICAM proteins are type I transmembrane glycoproteins, contain 2-9 immunoglobulin-like C2-type domains, and bind to the leukocyte adhesion LFA-1 protein. This protein is constitutively and abundantly expressed by all leucocytes and may be the most important ligand for LFA-1 in the initiation of the immune response. It functions not only as an adhesion molecule, but also as a potent signalling molecule. Alternative splicing results in multiple transcript variants encoding different isoforms. [provided by RefSeq, Feb 2016], | hsa04514:Cell adhesion molecules, |
| 3455 | interferon alpha and beta receptor subunit 2(IFNAR2) | The protein encoded by this gene is a type I membrane protein that forms one of the two chains of a receptor for interferons alpha and beta. Binding and activation of the receptor stimulates Janus protein kinases, which in turn phosphorylate several proteins, including STAT1 and STAT2. The protein belongs to the type II cytokine receptor family. Mutations in this gene are associated with Immunodeficiency 45. [provided by RefSeq, Jul 2020], | hsa04060:Cytokine-cytokine receptor interaction,hsa04151:PI3K-Akt signaling pathway,hsa04217:Necroptosis,hsa04380:Osteoclast differentiation,hsa04620:Toll-like receptor signaling pathway,hsa04621:NOD-like receptor signaling pathway,hsa04630:JAK-STAT signaling pathway,hsa04650:Natural killer cell mediated cytotoxicity,hsa05160:Hepatitis C,hsa05162:Measles,hsa05164:Influenza A,hsa05165:Human papillomavirus infection,hsa05167:Kaposi sarcoma-associated herpesvirus infection,hsa05168:Herpes simplex virus 1 infection,hsa05169:Epstein-Barr virus infection,hsa05171:Coronavirus disease - COVID-19,hsa05200:Pathways in cancer, |
| 3460 | interferon gamma receptor 2(IFNGR2) | This gene (IFNGR2) encodes the non-ligand-binding beta chain of the gamma interferon receptor. Human interferon-gamma receptor is a heterodimer of IFNGR1 and IFNGR2. Defects in IFNGR2 are a cause of mendelian susceptibility to mycobacterial disease (MSMD), also known as familial disseminated atypical mycobacterial infection. MSMD is a genetically heterogeneous disease with autosomal recessive, autosomal dominant or X-linked inheritance. [provided by RefSeq, Jul 2008], | hsa04060:Cytokine-cytokine receptor interaction,hsa04066:HIF-1 signaling pathway,hsa04217:Necroptosis,hsa04380:Osteoclast differentiation,hsa04630:JAK-STAT signaling pathway,hsa04650:Natural killer cell mediated cytotoxicity,hsa04658:Th1 and Th2 cell differentiation,hsa04659:Th17 cell differentiation,hsa05140:Leishmaniasis,hsa05142:Chagas disease,hsa05145:Toxoplasmosis,hsa05152:Tuberculosis,hsa05164:Influenza A,hsa05168:Herpes simplex virus 1 infection,hsa05200:Pathways in cancer,hsa05235:PD-L1 expression and PD-1 checkpoint pathway in cancer,hsa05321:Inflammatory bowel disease, |
| 8519 | interferon induced transmembrane protein 1(IFITM1) | Interferon-induced transmembrane (IFITM) proteins are a family of interferon induced antiviral proteins. The family contains five members, including IFITM1, IFITM2 and IFITM3 that belong to the CD225 superfamily. The protein encoded by this gene restricts cellular entry by diverse viral pathogens, such as influenza A virus, Ebola virus and Sars-CoV-2. [provided by RefSeq, Nov 2021], | hsa04662:B cell receptor signaling pathway, |
| 10581 | interferon induced transmembrane protein 2(IFITM2) | Interferon-induced transmembrane (IFITM) proteins are a family of interferon induced antiviral proteins. The family contains five members, including IFITM1, IFITM2 and IFITM3 and belong to the CD225 superfamily. The protein encoded by this gene restricts cellular entry by diverse viral pathogens, such as influenza A virus, Ebola virus and Sars-CoV-2. [provided by RefSeq, Nov 2021], |  |
| 3660 | interferon regulatory factor 2(IRF2) | IRF2 encodes interferon regulatory factor 2, a member of the interferon regulatory transcription factor (IRF) family. IRF2 competitively inhibits the IRF1-mediated transcriptional activation of interferons alpha and beta, and presumably other genes that employ IRF1 for transcription activation. However, IRF2 also functions as a transcriptional activator of histone H4. [provided by RefSeq, Jul 2008], |  |
| 3557 | interleukin 1 receptor antagonist(IL1RN) | The protein encoded by this gene is a member of the interleukin 1 cytokine family. This protein inhibits the activities of interleukin 1, alpha (IL1A) and interleukin 1, beta (IL1B), and modulates a variety of interleukin 1 related immune and inflammatory responses, particularly in the acute phase of infection and inflammation. This gene and five other closely related cytokine genes form a gene cluster spanning approximately 400 kb on chromosome 2. A polymorphism of this gene is reported to be associated with increased risk of osteoporotic fractures and gastric cancer. Several alternatively spliced transcript variants encoding distinct isoforms have been reported. [provided by RefSeq, Aug 2020], | hsa04060:Cytokine-cytokine receptor interaction, |
| 3597 | interleukin 13 receptor subunit alpha 1(IL13RA1) | The protein encoded by this gene is a subunit of the interleukin 13 receptor. This subunit forms a receptor complex with IL4 receptor alpha, a subunit shared by IL13 and IL4 receptors. This subunit serves as a primary IL13-binding subunit of the IL13 receptor, and may also be a component of IL4 receptors. This protein has been shown to bind tyrosine kinase TYK2, and thus may mediate the signaling processes that lead to the activation of JAK1, STAT3 and STAT6 induced by IL13 and IL4. [provided by RefSeq, Jul 2008], | hsa04060:Cytokine-cytokine receptor interaction,hsa04630:JAK-STAT signaling pathway,hsa05200:Pathways in cancer, |
| 3608 | interleukin enhancer binding factor 2(ILF2) | The protein encoded by this gene is a transcription factor required for T-cell expression of the interleukin 2 gene. It also binds RNA and is an essential component for encapsidation and protein priming of hepatitis B viral polymerase. The encoded 45 kDa protein (NF45, ILF2) forms a complex with the 90 kDa interleukin enhancer-binding factor 3 (NF90, ILF3), and this complex has been shown to affect the redistribution of nuclear mRNA to the cytoplasm, to repair DNA breaks by nonhomologous end joining, and to negatively regulate the microRNA processing pathway. Knockdown of NF45 or NF90 protein retards cell growth, possibly by inhibition of mRNA stabilization. Alternative splicing results in multiple transcript variants. Related pseudogenes have been found on chromosomes 3 and 14. [provided by RefSeq, Dec 2014], |  |
| 11275 | kelch like family member 2(KLHL2) | Enables actin binding activity and identical protein binding activity. Predicted to be involved in protein ubiquitination. Located in actin cytoskeleton. [provided by Alliance of Genome Resources, Apr 2022], |  |
| 22920 | kinesin associated protein 3(KIFAP3) | The small G protein GDP dissociation stimulator (smg GDS) is a regulator protein having two activities on a group of small G proteins including the Rho and Rap1 family members and Ki-Ras; one is to stimulate their GDP/GTP exchange reactions, and the other is to inhibit their interactions with membranes. The protein encoded by this gene contains 9 &apos;Armadillo&apos; repeats and interacts with the smg GDS protein through these repeats. This protein, which is highly concentrated around the endoplasmic reticulum, is phosphorylated by v-src, and this phosphorylation reduces the affinity of the protein for smg GDS. It is thought that this protein serves as a linker between human chromosome-associated polypeptide (HCAP) and KIF3A/B, a kinesin superfamily protein in the nucleus, and that it plays a role in the interaction of chromosomes with an ATPase motor protein. Several transcript variants encoding different isoforms have been found for this gene. [provided by RefSeq, Mar 2011], |  |
| 3939 | lactate dehydrogenase A(LDHA) | The protein encoded by this gene catalyzes the conversion of L-lactate and NAD to pyruvate and NADH in the final step of anaerobic glycolysis. The protein is found predominantly in muscle tissue and belongs to the lactate dehydrogenase family. Mutations in this gene have been linked to exertional myoglobinuria. Multiple transcript variants encoding different isoforms have been found for this gene. The human genome contains several non-transcribed pseudogenes of this gene. [provided by RefSeq, Sep 2008], | hsa00010:Glycolysis / Gluconeogenesis,hsa00270:Cysteine and methionine metabolism,hsa00620:Pyruvate metabolism,hsa00640:Propanoate metabolism,hsa01100:Metabolic pathways,hsa04066:HIF-1 signaling pathway,hsa04922:Glucagon signaling pathway,hsa05230:Central carbon metabolism in cancer, |
| 3945 | lactate dehydrogenase B(LDHB) | This gene encodes the B subunit of lactate dehydrogenase enzyme, which catalyzes the interconversion of pyruvate and lactate with concomitant interconversion of NADH and NAD+ in a post-glycolysis process. Alternatively spliced transcript variants have been found for this gene. Recent studies have shown that a C-terminally extended isoform is produced by use of an alternative in-frame translation termination codon via a stop codon readthrough mechanism, and that this isoform is localized in the peroxisomes. Mutations in this gene are associated with lactate dehydrogenase B deficiency. Pseudogenes have been identified on chromosomes X, 5 and 13. [provided by RefSeq, Feb 2016], | hsa00010:Glycolysis / Gluconeogenesis,hsa00270:Cysteine and methionine metabolism,hsa00620:Pyruvate metabolism,hsa00640:Propanoate metabolism,hsa01100:Metabolic pathways,hsa04066:HIF-1 signaling pathway,hsa04922:Glucagon signaling pathway,hsa05230:Central carbon metabolism in cancer, |
| 4001 | lamin B1(LMNB1) | This gene encodes one of the two B-type lamin proteins and is a component of the nuclear lamina. A duplication of this gene is associated with autosomal dominant adult-onset leukodystrophy (ADLD). Alternative splicing results in multiple transcript variants. [provided by RefSeq, Dec 2015], | hsa04210:Apoptosis, |
| 56925 | latexin(LXN) | This gene encodes the only known protein inhibitor of zinc-dependent metallocarboxypeptidases. The encoded protein, latexin, downregulates the population size of hematopoietic stem cells. This protein is found to be downregulated in cancer cells because of promoter hypermethylation. [provided by RefSeq, Jul 2020], |  |
| 54741 | leptin receptor overlapping transcript(LEPROT) | LEPROT is associated with the Golgi complex and endosomes and has a role in cell surface expression of growth hormone receptor (GHR; MIM 600946) and leptin receptor (OBR, or LEPR; MIM 601007), thereby altering receptor-mediated cell signaling (Couturier et al., 2007 [PubMed 18042720]; Touvier et al., 2009 [PubMed 19907080]).[supplied by OMIM, Jul 2010], |  |
| 11027 | leukocyte immunoglobulin like receptor A2(LILRA2) | This gene encodes a member of a family of immunoreceptors that are expressed predominantly on monocytes and B cells, and at lower levels on dendritic cells and natural killer cells. The encoded protein is an activating receptor that inhibits dendritic cell differentiation and antigen presentation and suppresses innate immune response. Alternatively spliced transcript variants encoding different isoforms have been found. This gene is located in a cluster of related genes on chromosome 19 and there is a pseudogene for this gene on chromosome 3. [provided by RefSeq, Mar 2014], | hsa04380:Osteoclast differentiation,hsa04662:B cell receptor signaling pathway, |
| 10859 | leukocyte immunoglobulin like receptor B1(LILRB1) | This gene is a member of the leukocyte immunoglobulin-like receptor (LIR) family, which is found in a gene cluster at chromosomal region 19q13.4. The encoded protein belongs to the subfamily B class of LIR receptors which contain two or four extracellular immunoglobulin domains, a transmembrane domain, and two to four cytoplasmic immunoreceptor tyrosine-based inhibitory motifs (ITIMs). The receptor is expressed on immune cells where it binds to MHC class I molecules on antigen-presenting cells and transduces a negative signal that inhibits stimulation of an immune response. It is thought to control inflammatory responses and cytotoxicity to help focus the immune response and limit autoreactivity. Multiple transcript variants encoding different isoforms have been found for this gene. [provided by RefSeq, Jul 2008], | hsa04380:Osteoclast differentiation,hsa04662:B cell receptor signaling pathway, |
| 10288 | leukocyte immunoglobulin like receptor B2(LILRB2) | This gene is a member of the leukocyte immunoglobulin-like receptor (LIR) family, which is found in a gene cluster at chromosomal region 19q13.4. The encoded protein belongs to the subfamily B class of LIR receptors which contain two or four extracellular immunoglobulin domains, a transmembrane domain, and two to four cytoplasmic immunoreceptor tyrosine-based inhibitory motifs (ITIMs). The receptor is expressed on immune cells where it binds to MHC class I molecules on antigen-presenting cells and transduces a negative signal that inhibits stimulation of an immune response. It is thought to control inflammatory responses and cytotoxicity to help focus the immune response and limit autoreactivity. Multiple transcript variants encoding different isoforms have been found for this gene. [provided by RefSeq, Jul 2008], | hsa04380:Osteoclast differentiation,hsa04662:B cell receptor signaling pathway, |
| 9404 | leupaxin(LPXN) | The product encoded by this gene is preferentially expressed in hematopoietic cells and belongs to the paxillin protein family. Similar to other members of this focal-adhesion-associated adaptor-protein family, it has four leucine-rich LD-motifs in the N-terminus and four LIM domains in the C-terminus. It may function in cell type-specific signaling by associating with PYK2, a member of focal adhesion kinase family. As a substrate for a tyrosine kinase in lymphoid cells, this protein may also function in, and be regulated by, tyrosine kinase activity. Alternative splicing results in multiple transcript variants encoding distinct isoforms.[provided by RefSeq, Jan 2009], |  |
| 3937 | lymphocyte cytosolic protein 2(LCP2) | This gene encodes an adapter protein that acts as a substrate of the T cell antigen receptor (TCR)-activated protein tyrosine kinase pathway. The encoded protein associates with growth factor receptor bound protein 2, and is thought to play a role TCR-mediated intracellular signal transduction. A similar protein in mouse plays a role in normal T-cell development and activation. Mice lacking this gene show subcutaneous and intraperitoneal fetal hemorrhaging, dysfunctional platelets and impaired viability. [provided by RefSeq, Nov 2016], | hsa04015:Rap1 signaling pathway,hsa04380:Osteoclast differentiation,hsa04611:Platelet activation,hsa04650:Natural killer cell mediated cytotoxicity,hsa04660:T cell receptor signaling pathway,hsa04664:Fc epsilon RI signaling pathway,hsa05135:Yersinia infection, |
| 54900 | lymphocyte transmembrane adaptor 1(LAX1) | Enables SH2 domain binding activity and protein kinase binding activity. Involved in several processes, including B cell activation; negative regulation of MAP kinase activity; and negative regulation of T cell activation. Located in Golgi apparatus; cytosol; and plasma membrane. Is integral component of membrane. [provided by Alliance of Genome Resources, Apr 2022], |  |
| 4050 | lymphotoxin beta(LTB) | Lymphotoxin beta is a type II membrane protein of the TNF family. It anchors lymphotoxin-alpha to the cell surface through heterotrimer formation. The predominant form on the lymphocyte surface is the lymphotoxin-alpha 1/beta 2 complex (e.g. 1 molecule alpha/2 molecules beta) and this complex is the primary ligand for the lymphotoxin-beta receptor. The minor complex is lymphotoxin-alpha 2/beta 1. LTB is an inducer of the inflammatory response system and involved in normal development of lymphoid tissue. Lymphotoxin-beta isoform b is unable to complex with lymphotoxin-alpha suggesting a function for lymphotoxin-beta which is independent of lympyhotoxin-alpha. Alternative splicing results in multiple transcript variants encoding different isoforms. [provided by RefSeq, Jul 2008], | hsa04060:Cytokine-cytokine receptor interaction,hsa04064:NF-kappa B signaling pathway,hsa05323:Rheumatoid arthritis, |
| 219972 | macrophage expressed 1(MPEG1) | Involved in defense response to Gram-negative bacterium and defense response to Gram-positive bacterium. Located in cytoplasmic vesicle. Implicated in primary immunodeficiency disease. [provided by Alliance of Genome Resources, Apr 2022], |  |
| 3108 | major histocompatibility complex, class II, DM alpha(HLA-DMA) | HLA-DMA belongs to the HLA class II alpha chain paralogues. This class II molecule is a heterodimer consisting of an alpha (DMA) and a beta chain (DMB), both anchored in the membrane. It is located in intracellular vesicles. DM plays a central role in the peptide loading of MHC class II molecules by helping to release the CLIP molecule from the peptide binding site. Class II molecules are expressed in antigen presenting cells (APC: B lymphocytes, dendritic cells, macrophages). The alpha chain is approximately 33-35 kDa and its gene contains 5 exons. Exon one encodes the leader peptide, exons 2 and 3 encode the two extracellular domains, exon 4 encodes the transmembrane domain and the cytoplasmic tail. [provided by RefSeq, Jul 2008], | hsa04145:Phagosome,hsa04514:Cell adhesion molecules,hsa04612:Antigen processing and presentation,hsa04640:Hematopoietic cell lineage,hsa04658:Th1 and Th2 cell differentiation,hsa04659:Th17 cell differentiation,hsa04672:Intestinal immune network for IgA production,hsa04940:Type I diabetes mellitus,hsa05140:Leishmaniasis,hsa05145:Toxoplasmosis,hsa05150:Staphylococcus aureus infection,hsa05152:Tuberculosis,hsa05164:Influenza A,hsa05166:Human T-cell leukemia virus 1 infection,hsa05168:Herpes simplex virus 1 infection,hsa05169:Epstein-Barr virus infection,hsa05310:Asthma,hsa05320:Autoimmune thyroid disease,hsa05321:Inflammatory bowel disease,hsa05322:Systemic lupus erythematosus,hsa05323:Rheumatoid arthritis,hsa05330:Allograft rejection,hsa05332:Graft-versus-host disease,hsa05416:Viral myocarditis, |
| 23608 | makorin ring finger protein 1(MKRN1) | This gene encodes a protein that belongs to a novel class of zinc finger proteins. The encoded protein functions as a transcriptional co-regulator, and as an E3 ubiquitin ligase that promotes the ubiquitination and proteasomal degradation of target proteins. The protein encoded by this gene is thought to regulate RNA polymerase II-catalyzed transcription. Substrates for this protein&apos;s E3 ubiquitin ligase activity include the capsid protein of the West Nile virus and the catalytic subunit of the telomerase ribonucleoprotein. This protein controls cell cycle arrest and apoptosis by regulating p21, a cell cycle regulator, and the tumor suppressor protein p53. Pseudogenes of this gene are present on chromosomes 1, 3, 9, 12 and 20, and on the X chromosome. Alternative splicing results in multiple transcript variants encoding different isoforms. [provided by RefSeq, Apr 2014], |  |
| 4200 | malic enzyme 2(ME2) | This gene encodes a mitochondrial NAD-dependent malic enzyme, a homotetrameric protein, that catalyzes the oxidative decarboxylation of malate to pyruvate. It had previously been weakly linked to a syndrome known as Friedreich ataxia that has since been shown to be the result of mutation in a completely different gene. Certain single-nucleotide polymorphism haplotypes of this gene have been shown to increase the risk for idiopathic generalized epilepsy. Alternatively spliced transcript variants encoding different isoforms found for this gene. [provided by RefSeq, Dec 2009], | hsa00620:Pyruvate metabolism,hsa01200:Carbon metabolism, |
| 22823 | metal response element binding transcription factor 2(MTF2) | Enables methylated histone binding activity and transcription corepressor binding activity. Predicted to be involved in several processes, including regulation of histone H3-K27 methylation; regulation of transcription by RNA polymerase II; and segment specification. Predicted to act upstream of or within cellular response to leukemia inhibitory factor. Located in cytoplasm; focal adhesion; and nucleoplasm. [provided by Alliance of Genome Resources, Apr 2022], |  |
| 22919 | microtubule associated protein RP/EB family member 1(MAPRE1) | The protein encoded by this gene was first identified by its binding to the APC protein which is often mutated in familial and sporadic forms of colorectal cancer. This protein localizes to microtubules, especially the growing ends, in interphase cells. During mitosis, the protein is associated with the centrosomes and spindle microtubules. The protein also associates with components of the dynactin complex and the intermediate chain of cytoplasmic dynein. Because of these associations, it is thought that this protein is involved in the regulation of microtubule structures and chromosome stability. This gene is a member of the RP/EB family. [provided by RefSeq, Jul 2008], |  |
| 55013 | mitochondrial calcium uniporter dominant negative subunit beta(MCUB) | Predicted to enable calcium channel inhibitor activity. Predicted to be involved in calcium import into the mitochondrion and mitochondrial calcium ion homeostasis. Located in mitochondrion and nucleoplasm. Is integral component of mitochondrial inner membrane. Part of uniplex complex. [provided by Alliance of Genome Resources, Apr 2022], |  |
| 5594 | mitogen-activated protein kinase 1(MAPK1) | This gene encodes a member of the MAP kinase family. MAP kinases, also known as extracellular signal-regulated kinases (ERKs), act as an integration point for multiple biochemical signals, and are involved in a wide variety of cellular processes such as proliferation, differentiation, transcription regulation and development. The activation of this kinase requires its phosphorylation by upstream kinases. Upon activation, this kinase translocates to the nucleus of the stimulated cells, where it phosphorylates nuclear targets. One study also suggests that this protein acts as a transcriptional repressor independent of its kinase activity. The encoded protein has been identified as a moonlighting protein based on its ability to perform mechanistically distinct functions. Two alternatively spliced transcript variants encoding the same protein, but differing in the UTRs, have been reported for this gene. [provided by RefSeq, Jan 2014], | hsa01521:EGFR tyrosine kinase inhibitor resistance,hsa01522:Endocrine resistance,hsa01524:Platinum drug resistance,hsa04010:MAPK signaling pathway,hsa04012:ErbB signaling pathway,hsa04014:Ras signaling pathway,hsa04015:Rap1 signaling pathway,hsa04022:cGMP-PKG signaling pathway,hsa04024:cAMP signaling pathway,hsa04062:Chemokine signaling pathway,hsa04066:HIF-1 signaling pathway,hsa04068:FoxO signaling pathway,hsa04071:Sphingolipid signaling pathway,hsa04072:Phospholipase D signaling pathway,hsa04114:Oocyte meiosis,hsa04140:Autophagy - animal,hsa04150:mTOR signaling pathway,hsa04151:PI3K-Akt signaling pathway,hsa04210:Apoptosis,hsa04218:Cellular senescence,hsa04261:Adrenergic signaling in cardiomyocytes,hsa04270:Vascular smooth muscle contraction,hsa04350:TGF-beta signaling pathway,hsa04360:Axon guidance,hsa04370:VEGF signaling pathway,hsa04371:Apelin signaling pathway,hsa04380:Osteoclast differentiation,hsa04510:Focal adhesion,hsa04520:Adherens junction,hsa04540:Gap junction,hsa04550:Signaling pathways regulating pluripotency of stem cells,hsa04611:Platelet activation,hsa04613:Neutrophil extracellular trap formation,hsa04620:Toll-like receptor signaling pathway,hsa04621:NOD-like receptor signaling pathway,hsa04625:C-type lectin receptor signaling pathway,hsa04650:Natural killer cell mediated cytotoxicity,hsa04657:IL-17 signaling pathway,hsa04658:Th1 and Th2 cell differentiation,hsa04659:Th17 cell differentiation,hsa04660:T cell receptor signaling pathway,hsa04662:B cell receptor signaling pathway,hsa04664:Fc epsilon RI signaling pathway,hsa04666:Fc gamma R-mediated phagocytosis,hsa04668:TNF signaling pathway,hsa04713:Circadian entrainment,hsa04720:Long-term potentiation,hsa04722:Neurotrophin signaling pathway,hsa04723:Retrograde endocannabinoid signaling,hsa04724:Glutamatergic synapse,hsa04725:Cholinergic synapse,hsa04726:Serotonergic synapse,hsa04730:Long-term depression,hsa04810:Regulation of actin cytoskeleton,hsa04910:Insulin signaling pathway,hsa04912:GnRH signaling pathway,hsa04914:Progesterone-mediated oocyte maturation,hsa04915:Estrogen signaling pathway,hsa04916:Melanogenesis,hsa04917:Prolactin signaling pathway,hsa04919:Thyroid hormone signaling pathway,hsa04921:Oxytocin signaling pathway,hsa04926:Relaxin signaling pathway,hsa04928:Parathyroid hormone synthesis, secretion and action,hsa04929:GnRH secretion,hsa04930:Type II diabetes mellitus,hsa04933:AGE-RAGE signaling pathway in diabetic complications,hsa04934:Cushing syndrome,hsa04935:Growth hormone synthesis, secretion and action,hsa04960:Aldosterone-regulated sodium reabsorption,hsa05010:Alzheimer disease,hsa05020:Prion disease,hsa05022:Pathways of neurodegeneration - multiple diseases,hsa05034:Alcoholism,hsa05130:Pathogenic Escherichia coli infection,hsa05131:Shigellosis,hsa05132:Salmonella infection,hsa05133:Pertussis,hsa05135:Yersinia infection,hsa05140:Leishmaniasis,hsa05142:Chagas disease,hsa05145:Toxoplasmosis,hsa05152:Tuberculosis,hsa05160:Hepatitis C,hsa05161:Hepatitis B,hsa05163:Human cytomegalovirus infection,hsa05164:Influenza A,hsa05165:Human papillomavirus infection,hsa05166:Human T-cell leukemia virus 1 infection,hsa05167:Kaposi sarcoma-associated herpesvirus infection,hsa05170:Human immunodeficiency virus 1 infection,hsa05171:Coronavirus disease - COVID-19,hsa05200:Pathways in cancer,hsa05203:Viral carcinogenesis,hsa05205:Proteoglycans in cancer,hsa05206:MicroRNAs in cancer,hsa05207:Chemical carcinogenesis - receptor activation,hsa05208:Chemical carcinogenesis - reactive oxygen species,hsa05210:Colorectal cancer,hsa05211:Renal cell carcinoma,hsa05212:Pancreatic cancer,hsa05213:Endometrial cancer,hsa05214:Glioma,hsa05215:Prostate cancer,hsa05216:Thyroid cancer,hsa05218:Melanoma,hsa05219:Bladder cancer,hsa05220:Chronic myeloid leukemia,hsa05221:Acute myeloid leukemia,hsa05223:Non-small cell lung cancer,hsa05224:Breast cancer,hsa05225:Hepatocellular carcinoma,hsa05226:Gastric cancer,hsa05230:Central carbon metabolism in cancer,hsa05231:Choline metabolism in cancer,hsa05235:PD-L1 expression and PD-1 checkpoint pathway in cancer,hsa05417:Lipid and atherosclerosis, |
| 1955 | multiple EGF like domains 9(MEGF9) | Predicted to be involved in several processes, including animal organ morphogenesis; cell migration; and substrate adhesion-dependent cell spreading. Predicted to be integral component of membrane. Predicted to be active in basement membrane. [provided by Alliance of Genome Resources, Apr 2022], |  |
| 124540 | musashi RNA binding protein 2(MSI2) | This gene encodes an RNA-binding protein that is a member of the Musashi protein family. The encoded protein is transcriptional regulator that targets genes involved in development and cell cycle regulation. Mutations in this gene are associated with poor prognosis in certain types of cancers. This gene has also been shown to be rearranged in certain cancer cells. [provided by RefSeq, Apr 2016], | hsa03015:mRNA surveillance pathway, |
| 4332 | myeloid cell nuclear differentiation antigen(MNDA) | The myeloid cell nuclear differentiation antigen (MNDA) is detected only in nuclei of cells of the granulocyte-monocyte lineage. A 200-amino acid region of human MNDA is strikingly similar to a region in the proteins encoded by a family of interferon-inducible mouse genes, designated Ifi-201, Ifi-202, and Ifi-203, that are not regulated in a cell- or tissue-specific fashion. The 1.8-kb MNDA mRNA, which contains an interferon-stimulated response element in the 5-prime untranslated region, was significantly upregulated in human monocytes exposed to interferon alpha. MNDA is located within 2,200 kb of FCER1A, APCS, CRP, and SPTA1. In its pattern of expression and/or regulation, MNDA resembles IFI16, suggesting that these genes participate in blood cell-specific responses to interferons. [provided by RefSeq, Jul 2008], |  |
| 26509 | myoferlin(MYOF) | Mutations in dysferlin, a protein associated with the plasma membrane, can cause muscle weakness that affects both proximal and distal muscles. The protein encoded by this gene is a type II membrane protein that is structurally similar to dysferlin. It is a member of the ferlin family and associates with both plasma and nuclear membranes. The protein contains C2 domains that play a role in calcium-mediated membrane fusion events, suggesting that it may be involved in membrane regeneration and repair. Two transcript variants encoding different isoforms have been found for this gene. Other possible variants have been detected, but their full-length nature has not been determined. [provided by RefSeq, Dec 2008], |  |
| 4542 | myosin IF(MYO1F) | Myosins are molecular motors that use the energy from ATP hydrolysis to generate force on actin filaments. The protein encoded by this gene is an unconventional myosin that may be involved in the intracellular movement of membrane-enclosed compartments. There is evidence to suggest that mutations in this gene can result in hearing loss. [provided by RefSeq, Jan 2017], | hsa05130:Pathogenic Escherichia coli infection, |
| 8776 | myotubularin related protein 1(MTMR1) | This gene encodes a member of the myotubularin related family of proteins. Members of this family contain the consensus sequence for the active site of protein tyrosine phosphatases. Alternatively spliced variants have been described but their biological validity has not been determined. [provided by RefSeq, Jul 2008], | hsa00562:Inositol phosphate metabolism,hsa01100:Metabolic pathways,hsa04070:Phosphatidylinositol signaling system, |
| 9107 | myotubularin related protein 6(MTMR6) | Enables phosphatidylinositol-3,5-bisphosphate phosphatase activity and phosphatidylinositol-3-phosphatase activity. Involved in phosphatidylinositol dephosphorylation. Located in cytoplasm and nuclear envelope. [provided by Alliance of Genome Resources, Apr 2022], | hsa00562:Inositol phosphate metabolism,hsa01100:Metabolic pathways,hsa04070:Phosphatidylinositol signaling system, |
| 4689 | neutrophil cytosolic factor 4(NCF4) | The protein encoded by this gene is a cytosolic regulatory component of the superoxide-producing phagocyte NADPH-oxidase, a multicomponent enzyme system important for host defense. This protein is preferentially expressed in cells of myeloid lineage. It interacts primarily with neutrophil cytosolic factor 2 (NCF2/p67-phox) to form a complex with neutrophil cytosolic factor 1 (NCF1/p47-phox), which further interacts with the small G protein RAC1 and translocates to the membrane upon cell stimulation. This complex then activates flavocytochrome b, the membrane-integrated catalytic core of the enzyme system. The PX domain of this protein can bind phospholipid products of the PI(3) kinase, which suggests its role in PI(3) kinase-mediated signaling events. The phosphorylation of this protein was found to negatively regulate the enzyme activity. Alternatively spliced transcript variants encoding distinct isoforms have been observed. [provided by RefSeq, Jul 2008], | hsa04145:Phagosome,hsa04380:Osteoclast differentiation,hsa04613:Neutrophil extracellular trap formation,hsa04670:Leukocyte transendothelial migration,hsa05020:Prion disease,hsa05140:Leishmaniasis,hsa05415:Diabetic cardiomyopathy,hsa05417:Lipid and atherosclerosis, |
| 116496 | niban apoptosis regulator 1(NIBAN1) | This gene encodes a member of the family with sequence similarity 129 protein family. This gene is highly expressed in several cancer cells and may serve as a prognostic marker for certain cancers. The encoded protein may play a role in regulating p53-mediated apoptosis. [provided by RefSeq, Sep 2016], |  |
| 4853 | notch receptor 2(NOTCH2) | This gene encodes a member of the Notch family. Members of this Type 1 transmembrane protein family share structural characteristics including an extracellular domain consisting of multiple epidermal growth factor-like (EGF) repeats, and an intracellular domain consisting of multiple, different domain types. Notch family members play a role in a variety of developmental processes by controlling cell fate decisions. The Notch signaling network is an evolutionarily conserved intercellular signaling pathway which regulates interactions between physically adjacent cells. In Drosophilia, notch interaction with its cell-bound ligands (delta, serrate) establishes an intercellular signaling pathway that plays a key role in development. Homologues of the notch-ligands have also been identified in human, but precise interactions between these ligands and the human notch homologues remain to be determined. This protein is cleaved in the trans-Golgi network, and presented on the cell surface as a heterodimer. This protein functions as a receptor for membrane bound ligands, and may play a role in vascular, renal and hepatic development. Two transcript variants encoding different isoforms have been found for this gene. [provided by RefSeq, Jan 2011], | hsa01522:Endocrine resistance,hsa04330:Notch signaling pathway,hsa04658:Th1 and Th2 cell differentiation,hsa04919:Thyroid hormone signaling pathway,hsa05165:Human papillomavirus infection,hsa05200:Pathways in cancer,hsa05206:MicroRNAs in cancer,hsa05207:Chemical carcinogenesis - receptor activation,hsa05224:Breast cancer, |
| 4778 | nuclear factor, erythroid 2(NFE2) | Enables several functions, including WW domain binding activity; identical protein binding activity; and protein N-terminus binding activity. Contributes to cis-regulatory region sequence-specific DNA binding activity. Predicted to be involved in regulation of transcription by RNA polymerase II. Predicted to act upstream of or within several processes, including labyrinthine layer blood vessel development; negative regulation of bone mineralization; and negative regulation of syncytium formation by plasma membrane fusion. Part of protein-DNA complex. [provided by Alliance of Genome Resources, Apr 2022], |  |
| 26502 | nuclear prelamin A recognition factor(NARF) | Several proteins have been found to be prenylated and methylated at their carboxyl-terminal ends. Prenylation was initially believed to be important only for membrane attachment. However, another role for prenylation appears to be its importance in protein-protein interactions. The only nuclear proteins known to be prenylated in mammalian cells are prelamin A- and B-type lamins. Prelamin A is farnesylated and carboxymethylated on the cysteine residue of a carboxyl-terminal CaaX motif. This post-translationally modified cysteine residue is removed from prelamin A when it is endoproteolytically processed into mature lamin A. The protein encoded by this gene binds to the prenylated prelamin A carboxyl-terminal tail domain. It may be a component of a prelamin A endoprotease complex. The encoded protein is located in the nucleus, where it partially colocalizes with the nuclear lamina. It shares limited sequence similarity with iron-only bacterial hydrogenases. Alternatively spliced transcript variants encoding different isoforms have been identified for this gene, including one with a novel exon that is generated by RNA editing. [provided by RefSeq, Jul 2008], |  |
| 8648 | nuclear receptor coactivator 1(NCOA1) | The protein encoded by this gene acts as a transcriptional coactivator for steroid and nuclear hormone receptors. It is a member of the p160/steroid receptor coactivator (SRC) family and like other family members has histone acetyltransferase activity and contains a nuclear localization signal, as well as bHLH and PAS domains. The product of this gene binds nuclear receptors directly and stimulates the transcriptional activities in a hormone-dependent fashion. Alternatively spliced transcript variants encoding different isoforms have been identified. [provided by RefSeq, Jul 2008], | hsa04915:Estrogen signaling pathway,hsa04919:Thyroid hormone signaling pathway,hsa05200:Pathways in cancer,hsa05224:Breast cancer, |
| 8204 | nuclear receptor interacting protein 1(NRIP1) | Nuclear receptor interacting protein 1 (NRIP1) is a nuclear protein that specifically interacts with the hormone-dependent activation domain AF2 of nuclear receptors. Also known as RIP140, this protein modulates transcriptional activity of the estrogen receptor. [provided by RefSeq, Jul 2008], |  |
| 64859 | nucleic acid binding protein 1(NABP1) | Single-stranded DNA (ssDNA)-binding proteins, such as OBFC2A, are ubiquitous and essential for a variety of DNA metabolic processes, including replication, recombination, and detection and repair of damage (Richard et al., 2008 [PubMed 18449195]).[supplied by OMIM, Jun 2008], |  |
| 11164 | nudix hydrolase 5(NUDT5) | This gene belongs to the Nudix (nucleoside diphosphate linked moiety X) hydrolase superfamily. The encoded enzyme catalyzes the hydrolysis of modified nucleoside diphosphates, including ADP-ribose (ADPR) and 8-oxoGua-containing 8-oxo-dADP and 8-oxo-dGDP. Protein-bound ADP ribose can be hazardous to the cell because it can modify some amino acid residues, resulting in the inhibition of ATP-activated potassium channels. 8-oxoGua is an oxidized form of guanine that can potentially alter genetic information by pairing with adenine and cytosine in RNA. Presence of 8-oxoGua in RNA results in formation of abnormal proteins due to translational errors. [provided by RefSeq, Aug 2013], | hsa00230:Purine metabolism,hsa01100:Metabolic pathways, |
| 10439 | olfactomedin 1(OLFM1) | This gene product shares extensive sequence similarity with the rat neuronal olfactomedin-related ER localized protein. While the exact function of the encoded protein is not known, its abundant expression in brain suggests that it may have an essential role in nerve tissue. Several alternatively spliced transcripts encoding different isoforms have been found for this gene. [provided by RefSeq, Jul 2008], |  |
| 4947 | ornithine decarboxylase antizyme 2(OAZ2) | The protein encoded by this gene belongs to the ornithine decarboxylase antizyme family, which plays a role in cell growth and proliferation by regulating intracellular polyamines. Expression of antizymes requires +1 ribosomal frameshifting, which is enhanced by high levels of polyamines. Antizymes in turn bind to and inhibit ornithine decarboxylase (ODC), the key enzyme in polyamine biosynthesis; thus, completing the auto-regulatory circuit. This gene encodes antizyme 2, the second member of the antizyme family. Like antizyme 1, antizyme 2 has broad tissue distribution, inhibits ODC activity and polyamine uptake, and stimulates ODC degradation in vivo; however, it fails to promote ODC degradation in vitro. Antizyme 2 is expressed at lower levels than antizyme 1, but is evolutionary more conserved, suggesting it likely has an important biological role. Studies also show different subcellular localization of antizymes 1 and 2, indicating specific function for each antizyme in discrete compartments of the cell. Alternatively spliced transcript variants have been found for this gene. [provided by RefSeq, Dec 2014], |  |
| 4957 | outer dense fiber of sperm tails 2(ODF2) | The outer dense fibers are cytoskeletal structures that surround the axoneme in the middle piece and principal piece of the sperm tail. The fibers function in maintaining the elastic structure and recoil of the sperm tail as well as in protecting the tail from shear forces during epididymal transport and ejaculation. Defects in the outer dense fibers lead to abnormal sperm morphology and infertility. This gene encodes one of the major outer dense fiber proteins. Alternative splicing results in multiple transcript variants. The longer transcripts, also known as &apos;Cenexins&apos;, encode proteins with a C-terminal extension that are differentially targeted to somatic centrioles and thought to be crucial for the formation of microtubule organizing centers. [provided by RefSeq, Oct 2010], |  |
| 29992 | paired immunoglobin like type 2 receptor alpha(PILRA) | Cell signaling pathways rely on a dynamic interaction between activating and inhibiting processes. SHP-1-mediated dephosphorylation of protein tyrosine residues is central to the regulation of several cell signaling pathways. Two types of inhibitory receptor superfamily members are immunoreceptor tyrosine-based inhibitory motif (ITIM)-bearing receptors and their non-ITIM-bearing, activating counterparts. Control of cell signaling via SHP-1 is thought to occur through a balance between PILRalpha-mediated inhibition and PILRbeta-mediated activation. These paired immunoglobulin-like receptor genes are located in a tandem head-to-tail orientation on chromosome 7. This particular gene encodes the ITIM-bearing member of the receptor pair, which functions in the inhibitory role. Alternative splicing has been observed at this locus and three variants, each encoding a distinct isoform, are described. [provided by RefSeq, Jul 2008], | hsa05168:Herpes simplex virus 1 infection, |
| 24145 | pannexin 1(PANX1) | The protein encoded by this gene belongs to the innexin family. Innexin family members are the structural components of gap junctions. This protein and pannexin 2 are abundantly expressed in central nerve system (CNS) and are coexpressed in various neuronal populations. Studies in Xenopus oocytes suggest that this protein alone and in combination with pannexin 2 may form cell type-specific gap junctions with distinct properties. [provided by RefSeq, Jul 2008], | hsa04621:NOD-like receptor signaling pathway, |
| 5445 | paraoxonase 2(PON2) | This gene encodes a member of the paraoxonase gene family, which includes three known members located adjacent to each other on the long arm of chromosome 7. The encoded protein is ubiquitously expressed in human tissues, membrane-bound, and may act as a cellular antioxidant, protecting cells from oxidative stress. Hydrolytic activity against acylhomoserine lactones, important bacterial quorum-sensing mediators, suggests the encoded protein may also play a role in defense responses to pathogenic bacteria. Mutations in this gene may be associated with vascular disease and a number of quantitative phenotypes related to diabetes. Alternatively spliced transcript variants encoding different isoforms have been described. [provided by RefSeq, Jul 2008], |  |
| 57162 | pellino E3 ubiquitin protein ligase 1(PELI1) | Enables ubiquitin protein ligase activity. Involved in several processes, including negative regulation of necroptotic process; protein polyubiquitination; and response to lipopolysaccharide. Predicted to be located in cytosol. [provided by Alliance of Genome Resources, Apr 2022], |  |
| 5066 | peptidylglycine alpha-amidating monooxygenase(PAM) | This gene encodes a multifunctional protein. The encoded preproprotein is proteolytically processed to generate the mature enzyme. This enzyme includes two domains with distinct catalytic activities, a peptidylglycine alpha-hydroxylating monooxygenase (PHM) domain and a peptidyl-alpha-hydroxyglycine alpha-amidating lyase (PAL) domain. These catalytic domains work sequentially to catalyze the conversion of neuroendocrine peptides to active alpha-amidated products. Alternative splicing results in multiple transcript variants, at least one of which encodes an isoform that is proteolytically processed. [provided by RefSeq, Jan 2016], |  |
| 5191 | peroxisomal biogenesis factor 7(PEX7) | This gene encodes the cytosolic receptor for the set of peroxisomal matrix enzymes targeted to the organelle by the peroxisome targeting signal 2 (PTS2). Defects in this gene cause peroxisome biogenesis disorders (PBDs), which are characterized by multiple defects in peroxisome function. There are at least 14 complementation groups for PBDs, with more than one phenotype being observed in cases falling into particular complementation groups. Although the clinical features of PBD patients vary, cells from all PBD patients exhibit a defect in the import of one or more classes of peroxisomal matrix proteins into the organelle. Defects in this gene have been associated with PBD complementation group 11 (PBD-CG11) disorders, rhizomelic chondrodysplasia punctata type 1 (RCDP1), and Refsum disease (RD). [provided by RefSeq, Oct 2008], | hsa04146:Peroxisome, |
| 10056 | phenylalanyl-tRNA synthetase subunit beta(FARSB) | This gene encodes a highly conserved enzyme that belongs to the aminoacyl-tRNA synthetase class IIc subfamily. This enzyme comprises the regulatory beta subunits that form a tetramer with two catalytic alpha subunits. In the presence of ATP, this tetramer is responsible for attaching L-phenylalanine to the terminal adenosine of the appropriate tRNA. A pseudogene located on chromosome 10 has been identified. Alternative splicing results in multiple transcript variants. [provided by RefSeq, Jan 2015], | hsa00970:Aminoacyl-tRNA biosynthesis, |
| 5286 | phosphatidylinositol-4-phosphate 3-kinase catalytic subunit type 2 alpha(PIK3C2A) | The protein encoded by this gene belongs to the phosphoinositide 3-kinase (PI3K) family. PI3-kinases play roles in signaling pathways involved in cell proliferation, oncogenic transformation, cell survival, cell migration, and intracellular protein trafficking. This protein contains a lipid kinase catalytic domain as well as a C-terminal C2 domain, a characteristic of class II PI3-kinases. C2 domains act as calcium-dependent phospholipid binding motifs that mediate translocation of proteins to membranes, and may also mediate protein-protein interactions. The PI3-kinase activity of this protein is not sensitive to nanomolar levels of the inhibitor wortmanin. This protein was shown to be able to be activated by insulin and may be involved in integrin-dependent signaling. [provided by RefSeq, Jul 2008], | hsa00562:Inositol phosphate metabolism,hsa01100:Metabolic pathways,hsa04070:Phosphatidylinositol signaling system,hsa05132:Salmonella infection, |
| 5305 | phosphatidylinositol-5-phosphate 4-kinase type 2 alpha(PIP4K2A) | Phosphatidylinositol-5,4-bisphosphate, the precursor to second messengers of the phosphoinositide signal transduction pathways, is thought to be involved in the regulation of secretion, cell proliferation, differentiation, and motility. The protein encoded by this gene is one of a family of enzymes capable of catalyzing the phosphorylation of phosphatidylinositol-5-phosphate on the fourth hydroxyl of the myo-inositol ring to form phosphatidylinositol-5,4-bisphosphate. The amino acid sequence of this enzyme does not show homology to other kinases, but the recombinant protein does exhibit kinase activity. This gene is a member of the phosphatidylinositol-5-phosphate 4-kinase family. [provided by RefSeq, Jul 2008], | hsa00562:Inositol phosphate metabolism,hsa01100:Metabolic pathways,hsa04070:Phosphatidylinositol signaling system,hsa04810:Regulation of actin cytoskeleton, |
| 8396 | phosphatidylinositol-5-phosphate 4-kinase type 2 beta(PIP4K2B) | The protein encoded by this gene catalyzes the phosphorylation of phosphatidylinositol-5-phosphate on the fourth hydroxyl of the myo-inositol ring to form phosphatidylinositol-5,4-bisphosphate. This gene is a member of the phosphatidylinositol-5-phosphate 4-kinase family. The encoded protein sequence does not show similarity to other kinases, but the protein does exhibit kinase activity. Additionally, the encoded protein interacts with p55 TNF receptor. [provided by RefSeq, Jul 2008], | hsa00562:Inositol phosphate metabolism,hsa01100:Metabolic pathways,hsa04070:Phosphatidylinositol signaling system,hsa04810:Regulation of actin cytoskeleton, |
| 5230 | phosphoglycerate kinase 1(PGK1) | The protein encoded by this gene is a glycolytic enzyme that catalyzes the conversion of 1,3-diphosphoglycerate to 3-phosphoglycerate. The encoded protein may also act as a cofactor for polymerase alpha. Additionally, this protein is secreted by tumor cells where it participates in angiogenesis by functioning to reduce disulfide bonds in the serine protease, plasmin, which consequently leads to the release of the tumor blood vessel inhibitor angiostatin. The encoded protein has been identified as a moonlighting protein based on its ability to perform mechanistically distinct functions. Deficiency of the enzyme is associated with a wide range of clinical phenotypes hemolytic anemia and neurological impairment. Pseudogenes of this gene have been defined on chromosomes 19, 21 and the X chromosome. [provided by RefSeq, Jan 2014], | hsa00010:Glycolysis / Gluconeogenesis,hsa01100:Metabolic pathways,hsa01200:Carbon metabolism,hsa01230:Biosynthesis of amino acids,hsa04066:HIF-1 signaling pathway, |
| 118788 | phosphoinositide-3-kinase adaptor protein 1(PIK3AP1) | Predicted to enable phosphatidylinositol 3-kinase regulatory subunit binding activity and signaling receptor binding activity. Predicted to be involved in regulation of inflammatory response; regulation of signal transduction; and toll-like receptor signaling pathway. Predicted to be located in cytoplasm and membrane. Predicted to be active in cytosol. [provided by Alliance of Genome Resources, Apr 2022], | hsa04151:PI3K-Akt signaling pathway,hsa04662:B cell receptor signaling pathway, |
| 79887 | phospholipase B domain containing 1(PLBD1) | Predicted to enable phospholipase activity. Predicted to be involved in phospholipid catabolic process. Located in extracellular space. [provided by Alliance of Genome Resources, Apr 2022], |  |
| 5359 | phospholipid scramblase 1(PLSCR1) | This gene encodes a phospholipid scramblase family member. The encoded protein is involved in disruption of the asymmetrical distribution of phospholipids between the inner and outer leaflets of the plasma membrane, resulting in externalization of phosphatidylserine. This cell membrane disruption plays an important role in the blood coagulation cascade as well as macrophage clearing of apoptotic cells. The encoded protein has additionally been implicated in gene regulation and interferon-induced antiviral responses. [provided by RefSeq, May 2022], |  |
| 5264 | phytanoyl-CoA 2-hydroxylase(PHYH) | This gene is a member of the PhyH family and encodes a peroxisomal protein that is involved in the alpha-oxidation of 3-methyl branched fatty acids. Specifically, this protein converts phytanoyl-CoA to 2-hydroxyphytanoyl-CoA. Mutations in this gene have been associated with Refsum disease (RD) and deficient protein activity has been associated with Zellweger syndrome and rhizomelic chondrodysplasia punctata. Alternate transcriptional splice variants, encoding different isoforms, have been characterized. [provided by RefSeq, Jul 2008], | hsa04146:Peroxisome, |
| 5724 | platelet activating factor receptor(PTAFR) | This gene encodes a seven-transmembrane G-protein-coupled receptor for platelet-activating factor (PAF) that localizes to lipid rafts and/or caveolae in the cell membrane. PAF (1-0-alkyl-2-acetyl-sn-glycero-3-phosphorylcholine) is a phospholipid that plays a significant role in oncogenic transformation, tumor growth, angiogenesis, metastasis, and pro-inflammatory processes. Binding of PAF to the PAF-receptor (PAFR) stimulates numerous signal transduction pathways including phospholipase C, D, A2, mitogen-activated protein kinases (MAPKs), and the phosphatidylinositol-calcium second messenger system. Following PAFR activation, cells become rapidly desensitized and this refractory state is dependent on PAFR phosphorylation, internalization, and down-regulation. Alternative splicing results in multiple transcript variants. [provided by RefSeq, Aug 2011], | hsa04020:Calcium signaling pathway,hsa04080:Neuroactive ligand-receptor interaction,hsa05150:Staphylococcus aureus infection, |
| 10154 | plexin C1(PLXNC1) | This gene encodes a member of the plexin family. Plexins are transmembrane receptors for semaphorins, a large family of proteins that regulate axon guidance, cell motility and migration, and the immune response. The encoded protein and its ligand regulate melanocyte adhesion, and viral semaphorins may modulate the immune response by binding to this receptor. The encoded protein may be a tumor suppressor protein for melanoma. Alternatively spliced transcript variants have been observed for this gene. [provided by RefSeq, Jan 2011], | hsa04360:Axon guidance, |
| 79668 | poly(ADP-ribose) polymerase family member 8(PARP8) | Enables protein ADP-ribosylase activity. Involved in protein auto-ADP-ribosylation and protein mono-ADP-ribosylation. [provided by Alliance of Genome Resources, Apr 2022], |  |
| 83666 | poly(ADP-ribose) polymerase family member 9(PARP9) | Enables several functions, including ADP-D-ribose binding activity; NAD+ ADP-ribosyltransferase activity; and STAT family protein binding activity. Involved in several processes, including positive regulation of nitrogen compound metabolic process; regulation of defense response; and regulation of gene expression. Located in several cellular components, including mitochondrion; nucleoplasm; and site of DNA damage. Part of protein-containing complex. Colocalizes with nucleus. [provided by Alliance of Genome Resources, Apr 2022], |  |
| 9991 | polypyrimidine tract binding protein 3(PTBP3) | The protein encoded by this gene binds RNA and is a regulator of cell differentiation. The encoded protein preferentially binds to poly(G) and poly(U) sequences in vitro. Several transcript variants encoding different isoforms have been found for this gene. [provided by RefSeq, Oct 2011], |  |
| 55851 | presenilin enhancer, gamma-secretase subunit(PSENEN) | Presenilins, which are components of the gamma-secretase protein complex, are required for intramembranous processing of some type I transmembrane proteins, such as the Notch proteins and the beta-amyloid precursor protein. Signaling by Notch receptors mediates a wide range of developmental cell fates. Processing of the beta-amyloid precursor protein generates neurotoxic amyloid beta peptides, the major component of senile plaques associated with Alzheimer&apos;s disease. This gene encodes a protein that is required for Notch pathway signaling, and for the activity and accumulation of gamma-secretase. Mutations resulting in haploinsufficiency for this gene cause familial acne inversa-2 (ACNINV2). Alternative splicing results in multiple transcript variants. [provided by RefSeq, Jul 2013], | hsa04330:Notch signaling pathway,hsa05010:Alzheimer disease, |
| 5579 | protein kinase C beta(PRKCB) | Protein kinase C (PKC) is a family of serine- and threonine-specific protein kinases that can be activated by calcium and second messenger diacylglycerol. PKC family members phosphorylate a wide variety of protein targets and are known to be involved in diverse cellular signaling pathways. PKC family members also serve as major receptors for phorbol esters, a class of tumor promoters. Each member of the PKC family has a specific expression profile and is believed to play a distinct role in cells. The protein encoded by this gene is one of the PKC family members. This protein kinase has been reported to be involved in many different cellular functions, such as B cell activation, apoptosis induction, endothelial cell proliferation, and intestinal sugar absorption. Studies in mice also suggest that this kinase may also regulate neuronal functions and correlate fear-induced conflict behavior after stress. Alternatively spliced transcript variants encoding distinct isoforms have been reported. [provided by RefSeq, Jul 2008], | hsa01521:EGFR tyrosine kinase inhibitor resistance,hsa04010:MAPK signaling pathway,hsa04012:ErbB signaling pathway,hsa04014:Ras signaling pathway,hsa04015:Rap1 signaling pathway,hsa04020:Calcium signaling pathway,hsa04062:Chemokine signaling pathway,hsa04064:NF-kappa B signaling pathway,hsa04066:HIF-1 signaling pathway,hsa04070:Phosphatidylinositol signaling system,hsa04071:Sphingolipid signaling pathway,hsa04150:mTOR signaling pathway,hsa04270:Vascular smooth muscle contraction,hsa04310:Wnt signaling pathway,hsa04370:VEGF signaling pathway,hsa04510:Focal adhesion,hsa04540:Gap junction,hsa04613:Neutrophil extracellular trap formation,hsa04650:Natural killer cell mediated cytotoxicity,hsa04662:B cell receptor signaling pathway,hsa04666:Fc gamma R-mediated phagocytosis,hsa04670:Leukocyte transendothelial migration,hsa04713:Circadian entrainment,hsa04720:Long-term potentiation,hsa04723:Retrograde endocannabinoid signaling,hsa04724:Glutamatergic synapse,hsa04725:Cholinergic synapse,hsa04726:Serotonergic synapse,hsa04727:GABAergic synapse,hsa04728:Dopaminergic synapse,hsa04730:Long-term depression,hsa04750:Inflammatory mediator regulation of TRP channels,hsa04911:Insulin secretion,hsa04912:GnRH signaling pathway,hsa04916:Melanogenesis,hsa04918:Thyroid hormone synthesis,hsa04919:Thyroid hormone signaling pathway,hsa04921:Oxytocin signaling pathway,hsa04925:Aldosterone synthesis and secretion,hsa04928:Parathyroid hormone synthesis, secretion and action,hsa04929:GnRH secretion,hsa04931:Insulin resistance,hsa04933:AGE-RAGE signaling pathway in diabetic complications,hsa04935:Growth hormone synthesis, secretion and action,hsa04960:Aldosterone-regulated sodium reabsorption,hsa04961:Endocrine and other factor-regulated calcium reabsorption,hsa04970:Salivary secretion,hsa04971:Gastric acid secretion,hsa04972:Pancreatic secretion,hsa04973:Carbohydrate digestion and absorption,hsa05017:Spinocerebellar ataxia,hsa05022:Pathways of neurodegeneration - multiple diseases,hsa05031:Amphetamine addiction,hsa05032:Morphine addiction,hsa05140:Leishmaniasis,hsa05143:African trypanosomiasis,hsa05146:Amoebiasis,hsa05161:Hepatitis B,hsa05163:Human cytomegalovirus infection,hsa05164:Influenza A,hsa05170:Human immunodeficiency virus 1 infection,hsa05171:Coronavirus disease - COVID-19,hsa05200:Pathways in cancer,hsa05205:Proteoglycans in cancer,hsa05206:MicroRNAs in cancer,hsa05207:Chemical carcinogenesis - receptor activation,hsa05214:Glioma,hsa05223:Non-small cell lung cancer,hsa05225:Hepatocellular carcinoma,hsa05231:Choline metabolism in cancer,hsa05415:Diabetic cardiomyopathy, |
| 129285 | protein phosphatase 1 regulatory subunit 21(PPP1R21) | Located in early endosome. [provided by Alliance of Genome Resources, Apr 2022], |  |
| 5530 | protein phosphatase 3 catalytic subunit alpha(PPP3CA) | Enables several functions, including ATPase binding activity; calmodulin binding activity; and calmodulin-dependent protein phosphatase activity. Involved in several processes, including calcineurin-NFAT signaling cascade; peptidyl-serine dephosphorylation; and response to calcium ion. Located in several cellular components, including cytosol; dendritic spine; and nucleoplasm. Part of calcineurin complex. Colocalizes with cytoplasmic side of plasma membrane. Implicated in developmental and epileptic encephalopathy 91. Biomarker of focal segmental glomerulosclerosis and schizophrenia. [provided by Alliance of Genome Resources, Apr 2022], | hsa04010:MAPK signaling pathway,hsa04020:Calcium signaling pathway,hsa04022:cGMP-PKG signaling pathway,hsa04114:Oocyte meiosis,hsa04218:Cellular senescence,hsa04310:Wnt signaling pathway,hsa04360:Axon guidance,hsa04370:VEGF signaling pathway,hsa04380:Osteoclast differentiation,hsa04625:C-type lectin receptor signaling pathway,hsa04650:Natural killer cell mediated cytotoxicity,hsa04658:Th1 and Th2 cell differentiation,hsa04659:Th17 cell differentiation,hsa04660:T cell receptor signaling pathway,hsa04662:B cell receptor signaling pathway,hsa04720:Long-term potentiation,hsa04724:Glutamatergic synapse,hsa04728:Dopaminergic synapse,hsa04921:Oxytocin signaling pathway,hsa04922:Glucagon signaling pathway,hsa04924:Renin secretion,hsa05010:Alzheimer disease,hsa05014:Amyotrophic lateral sclerosis,hsa05020:Prion disease,hsa05022:Pathways of neurodegeneration - multiple diseases,hsa05031:Amphetamine addiction,hsa05152:Tuberculosis,hsa05163:Human cytomegalovirus infection,hsa05166:Human T-cell leukemia virus 1 infection,hsa05167:Kaposi sarcoma-associated herpesvirus infection,hsa05170:Human immunodeficiency virus 1 infection,hsa05235:PD-L1 expression and PD-1 checkpoint pathway in cancer,hsa05417:Lipid and atherosclerosis, |
| 5532 | protein phosphatase 3 catalytic subunit beta(PPP3CB) | Enables several functions, including calmodulin binding activity; calmodulin-dependent protein phosphatase activity; and protein phosphatase 2B binding activity. Involved in calcineurin-NFAT signaling cascade; positive regulation of transcription by RNA polymerase II; and protein dephosphorylation. Located in cytoplasm. Part of calcineurin complex. Implicated in aortic valve stenosis. Biomarker of focal segmental glomerulosclerosis and schizophrenia. [provided by Alliance of Genome Resources, Apr 2022], | hsa04010:MAPK signaling pathway,hsa04020:Calcium signaling pathway,hsa04022:cGMP-PKG signaling pathway,hsa04114:Oocyte meiosis,hsa04218:Cellular senescence,hsa04310:Wnt signaling pathway,hsa04360:Axon guidance,hsa04370:VEGF signaling pathway,hsa04380:Osteoclast differentiation,hsa04625:C-type lectin receptor signaling pathway,hsa04650:Natural killer cell mediated cytotoxicity,hsa04658:Th1 and Th2 cell differentiation,hsa04659:Th17 cell differentiation,hsa04660:T cell receptor signaling pathway,hsa04662:B cell receptor signaling pathway,hsa04720:Long-term potentiation,hsa04724:Glutamatergic synapse,hsa04728:Dopaminergic synapse,hsa04921:Oxytocin signaling pathway,hsa04922:Glucagon signaling pathway,hsa04924:Renin secretion,hsa05010:Alzheimer disease,hsa05014:Amyotrophic lateral sclerosis,hsa05020:Prion disease,hsa05022:Pathways of neurodegeneration - multiple diseases,hsa05031:Amphetamine addiction,hsa05152:Tuberculosis,hsa05163:Human cytomegalovirus infection,hsa05166:Human T-cell leukemia virus 1 infection,hsa05167:Kaposi sarcoma-associated herpesvirus infection,hsa05170:Human immunodeficiency virus 1 infection,hsa05235:PD-L1 expression and PD-1 checkpoint pathway in cancer,hsa05417:Lipid and atherosclerosis, |
| 5534 | protein phosphatase 3 regulatory subunit B, alpha(PPP3R1) | Enables cyclosporin A binding activity; phosphatase binding activity; and protein domain specific binding activity. Involved in calcineurin-NFAT signaling cascade and positive regulation of transcription by RNA polymerase II. Part of calcineurin complex. Implicated in Alzheimer&apos;s disease and dilated cardiomyopathy. [provided by Alliance of Genome Resources, Apr 2022], | hsa04010:MAPK signaling pathway,hsa04020:Calcium signaling pathway,hsa04022:cGMP-PKG signaling pathway,hsa04114:Oocyte meiosis,hsa04218:Cellular senescence,hsa04310:Wnt signaling pathway,hsa04360:Axon guidance,hsa04370:VEGF signaling pathway,hsa04380:Osteoclast differentiation,hsa04625:C-type lectin receptor signaling pathway,hsa04650:Natural killer cell mediated cytotoxicity,hsa04658:Th1 and Th2 cell differentiation,hsa04659:Th17 cell differentiation,hsa04660:T cell receptor signaling pathway,hsa04662:B cell receptor signaling pathway,hsa04720:Long-term potentiation,hsa04724:Glutamatergic synapse,hsa04921:Oxytocin signaling pathway,hsa04922:Glucagon signaling pathway,hsa04924:Renin secretion,hsa05010:Alzheimer disease,hsa05014:Amyotrophic lateral sclerosis,hsa05020:Prion disease,hsa05022:Pathways of neurodegeneration - multiple diseases,hsa05031:Amphetamine addiction,hsa05152:Tuberculosis,hsa05163:Human cytomegalovirus infection,hsa05166:Human T-cell leukemia virus 1 infection,hsa05167:Kaposi sarcoma-associated herpesvirus infection,hsa05170:Human immunodeficiency virus 1 infection,hsa05235:PD-L1 expression and PD-1 checkpoint pathway in cancer,hsa05417:Lipid and atherosclerosis, |
| 5494 | protein phosphatase, Mg2+/Mn2+ dependent 1A(PPM1A) | The protein encoded by this gene is a member of the PP2C family of Ser/Thr protein phosphatases. PP2C family members are known to be negative regulators of cell stress response pathways. This phosphatase dephosphorylates, and negatively regulates the activities of, MAP kinases and MAP kinase kinases. It has been shown to inhibit the activation of p38 and JNK kinase cascades induced by environmental stresses. This phosphatase can also dephosphorylate cyclin-dependent kinases, and thus may be involved in cell cycle control. Overexpression of this phosphatase is reported to activate the expression of the tumor suppressor gene TP53/p53, which leads to G2/M cell cycle arrest and apoptosis. Three alternatively spliced transcript variants encoding distinct isoforms have been described. [provided by RefSeq, Jul 2008], | hsa04010:MAPK signaling pathway, |
| 7803 | protein tyrosine phosphatase 4A1(PTP4A1) | This gene encodes a member of a small class of prenylated protein tyrosine phosphatases (PTPs), which contain a PTP domain and a characteristic C-terminal prenylation motif. The encoded protein is a cell signaling molecule that plays regulatory roles in a variety of cellular processes, including cell proliferation and migration. The protein may also be involved in cancer development and metastasis. This tyrosine phosphatase is a nuclear protein, but may associate with plasma membrane by means of its prenylation motif. Pseudogenes related to this gene are located on chromosomes 1, 2, 5, 7, 11 and X. [provided by RefSeq, Jun 2013], |  |
| 5775 | protein tyrosine phosphatase non-receptor type 4(PTPN4) | The protein encoded by this gene is a member of the protein tyrosine phosphatase (PTP) family. PTPs are known to be signaling molecules that regulate a variety of cellular processes including cell growth, differentiation, mitotic cycle, and oncogenic transformation. This protein contains a C-terminal PTP domain and an N-terminal domain homologous to the band 4.1 superfamily of cytoskeletal-associated proteins. This PTP has been shown to interact with glutamate receptor delta 2 and epsilon subunits, and is thought to play a role in signalling downstream of the glutamate receptors through tyrosine dephosphorylation. [provided by RefSeq, Jul 2008], |  |
| 5788 | protein tyrosine phosphatase receptor type C(PTPRC) | The protein encoded by this gene is a member of the protein tyrosine phosphatase (PTP) family. PTPs are known to be signaling molecules that regulate a variety of cellular processes including cell growth, differentiation, mitosis, and oncogenic transformation. This PTP contains an extracellular domain, a single transmembrane segment and two tandem intracytoplasmic catalytic domains, and thus is classified as a receptor type PTP. This PTP has been shown to be an essential regulator of T- and B-cell antigen receptor signaling. It functions through either direct interaction with components of the antigen receptor complexes, or by activating various Src family kinases required for the antigen receptor signaling. This PTP also suppresses JAK kinases, and thus functions as a regulator of cytokine receptor signaling. Alternatively spliced transcripts variants of this gene, which encode distinct isoforms, have been reported. [provided by RefSeq, Jun 2012], | hsa04514:Cell adhesion molecules,hsa04660:T cell receptor signaling pathway,hsa04666:Fc gamma R-mediated phagocytosis,hsa05132:Salmonella infection,hsa05340:Primary immunodeficiency, |
| 5791 | protein tyrosine phosphatase receptor type E(PTPRE) | The protein encoded by this gene is a member of the protein tyrosine phosphatase (PTP) family. PTPs are known to be signaling molecules that regulate a variety of cellular processes including cell growth, differentiation, mitotic cycle, and oncogenic transformation. Several alternatively spliced transcript variants of this gene have been reported, at least two of which encode a receptor-type PTP that possesses a short extracellular domain, a single transmembrane region, and two tandem intracytoplasmic catalytic domains; another one encodes a PTP that contains a distinct hydrophilic N-terminus, and thus represents a nonreceptor-type isoform of this PTP. Studies of the similar gene in mice suggested the regulatory roles of this PTP in RAS related signal transduction pathways, cytokine-induced SATA signaling, as well as the activation of voltage-gated K+ channels. [provided by RefSeq, Oct 2015], |  |
| 53829 | purinergic receptor P2Y13(P2RY13) | The product of this gene belongs to the family of G-protein coupled receptors. This family has several receptor subtypes with different pharmacological selectivity, which overlaps in some cases, for various adenosine and uridine nucleotides. This receptor is activated by ADP. [provided by RefSeq, Sep 2008], | hsa04080:Neuroactive ligand-receptor interaction, |
| 5160 | pyruvate dehydrogenase E1 subunit alpha 1(PDHA1) | The pyruvate dehydrogenase (PDH) complex is a nuclear-encoded mitochondrial multienzyme complex that catalyzes the overall conversion of pyruvate to acetyl-CoA and CO(2), and provides the primary link between glycolysis and the tricarboxylic acid (TCA) cycle. The PDH complex is composed of multiple copies of three enzymatic components: pyruvate dehydrogenase (E1), dihydrolipoamide acetyltransferase (E2) and lipoamide dehydrogenase (E3). The E1 enzyme is a heterotetramer of two alpha and two beta subunits. This gene encodes the E1 alpha 1 subunit containing the E1 active site, and plays a key role in the function of the PDH complex. Mutations in this gene are associated with pyruvate dehydrogenase E1-alpha deficiency and X-linked Leigh syndrome. Alternatively spliced transcript variants encoding different isoforms have been found for this gene.[provided by RefSeq, Mar 2010], | hsa00010:Glycolysis / Gluconeogenesis,hsa00020:Citrate cycle (TCA cycle),hsa00620:Pyruvate metabolism,hsa01100:Metabolic pathways,hsa01200:Carbon metabolism,hsa04066:HIF-1 signaling pathway,hsa04922:Glucagon signaling pathway,hsa05230:Central carbon metabolism in cancer,hsa05415:Diabetic cardiomyopathy, |
| 5166 | pyruvate dehydrogenase kinase 4(PDK4) | This gene is a member of the PDK/BCKDK protein kinase family and encodes a mitochondrial protein with a histidine kinase domain. This protein is located in the matrix of the mitrochondria and inhibits the pyruvate dehydrogenase complex by phosphorylating one of its subunits, thereby contributing to the regulation of glucose metabolism. Expression of this gene is regulated by glucocorticoids, retinoic acid and insulin. [provided by RefSeq, Jul 2008], | hsa05415:Diabetic cardiomyopathy, |
| 23475 | quinolinate phosphoribosyltransferase(QPRT) | This gene encodes a key enzyme in catabolism of quinolinate, an intermediate in the tryptophan-nicotinamide adenine dinucleotide pathway. Quinolinate acts as a most potent endogenous exitotoxin to neurons. Elevation of quinolinate levels in the brain has been linked to the pathogenesis of neurodegenerative disorders such as epilepsy, Alzheimer&apos;s disease, and Huntington&apos;s disease. Alternative splicing results in multiple transcript variants. [provided by RefSeq, Dec 2015], | hsa00760:Nicotinate and nicotinamide metabolism,hsa01100:Metabolic pathways,hsa01240:Biosynthesis of cofactors, |
| 5962 | radixin(RDX) | Radixin is a cytoskeletal protein that may be important in linking actin to the plasma membrane. It is highly similar in sequence to both ezrin and moesin. The radixin gene has been localized by fluorescence in situ hybridization to 11q23. A truncated version representing a pseudogene (RDXP2) was assigned to Xp21.3. Another pseudogene that seemed to lack introns (RDXP1) was mapped to 11p by Southern and PCR analyses. Multiple alternatively spliced transcript variants encoding different isoforms have been found for this gene. [provided by RefSeq, May 2012], | hsa04530:Tight junction,hsa04810:Regulation of actin cytoskeleton,hsa05205:Proteoglycans in cancer,hsa05206:MicroRNAs in cancer, |
| 266747 | ral guanine nucleotide dissociation stimulator like 4(RGL4) | This oncogene encodes a protein similar to guanine nucleotide exchange factor Ral guanine dissociation stimulator. Increased expression of this gene leads to translocation of the encoded protein to the cell membrane. The encoded protein can activate several pathways, including the Ras-Raf-MEK-ERK cascade. [provided by RefSeq, Jul 2016], |  |
| 5900 | ral guanine nucleotide dissociation stimulator(RALGDS) | Guanine nucleotide dissociation stimulators (GDSs, or exchange factors), such as RALGDS, are effectors of Ras-related GTPases (see MIM 190020) that participate in signaling for a variety of cellular processes.[supplied by OMIM, Nov 2010], | hsa04014:Ras signaling pathway,hsa04015:Rap1 signaling pathway,hsa04072:Phospholipase D signaling pathway,hsa05200:Pathways in cancer,hsa05210:Colorectal cancer,hsa05212:Pancreatic cancer,hsa05231:Choline metabolism in cancer, |
| 391 | ras homolog family member G(RHOG) | This gene encodes a member of the Rho family of small GTPases, which cycle between inactive GDP-bound and active GTP-bound states and function as molecular switches in signal transduction cascades. Rho proteins promote reorganization of the actin cytoskeleton and regulate cell shape, attachment, and motility. The encoded protein facilitates translocation of a functional guanine nucleotide exchange factor (GEF) complex from the cytoplasm to the plasma membrane where ras-related C3 botulinum toxin substrate 1 is activated to promote lamellipodium formation and cell migration. Two related pseudogene have been identified on chromosomes 20 and X. [provided by RefSeq, Aug 2011], | hsa05100:Bacterial invasion of epithelial cells,hsa05132:Salmonella infection,hsa05135:Yersinia infection, |
| 6004 | regulator of G protein signaling 16(RGS16) | The protein encoded by this gene belongs to the &apos;regulator of G protein signaling&apos; family. It inhibits signal transduction by increasing the GTPase activity of G protein alpha subunits. It also may play a role in regulating the kinetics of signaling in the phototransduction cascade. [provided by RefSeq, Jul 2008], |  |
| 10287 | regulator of G protein signaling 19(RGS19) | G proteins mediate a number of cellular processes. The protein encoded by this gene belongs to the RGS (regulators of G-protein signaling) family and specifically interacts with G protein, GAI3. This protein is a guanosine triphosphatase-activating protein that functions to down-regulate Galpha i/Galpha q-linked signaling. Alternatively spliced transcript variants encoding the same protein isoform have been found for this gene. [provided by RefSeq, Jul 2008], |  |
| 55196 | retroelement silencing factor 1(RESF1) | Predicted to enable histone binding activity and histone methyltransferase binding activity. Predicted to be involved in negative regulation of single stranded viral RNA replication via double stranded DNA intermediate and positive regulation of DNA methylation-dependent heterochromatin assembly. Predicted to act upstream of or within response to bacterium. Predicted to be located in nucleus. Predicted to colocalize with gamma-tubulin complex. [provided by Alliance of Genome Resources, Apr 2022], |  |
| 8933 | retrotransposon Gag like 8C(RTL8C) | Predicted to be located in plasma membrane. [provided by Alliance of Genome Resources, Apr 2022], |  |
| 64080 | ribokinase(RBKS) | This gene encodes a member of the carbohydrate kinase PfkB family. The encoded protein phosphorylates ribose to form ribose-5-phosphate in the presence of ATP and magnesium as a first step in ribose metabolism. Alternative splicing results in multiple transcript variants. [provided by RefSeq, Dec 2013], | hsa00030:Pentose phosphate pathway,hsa01100:Metabolic pathways, |
| 57494 | ribosomal modification protein rimK like family member B(RIMKLB) | Predicted to enable N-acetyl-L-aspartate-L-glutamate ligase activity and citrate-L-glutamate ligase activity. Predicted to be involved in glutamine family amino acid metabolic process. Predicted to be located in cytosol. Predicted to be active in cytoplasm. [provided by Alliance of Genome Resources, Apr 2022], | hsa00250:Alanine, aspartate and glutamate metabolism,hsa01100:Metabolic pathways, |
| 6147 | ribosomal protein L23a(RPL23A) | Ribosomes, the organelles that catalyze protein synthesis, consist of a small 40S subunit and a large 60S subunit. Together these subunits are composed of 4 RNA species and approximately 80 structurally distinct proteins. This gene encodes a ribosomal protein that is a component of the 60S subunit. The protein belongs to the L23P family of ribosomal proteins. It is located in the cytoplasm. The protein may be one of the target molecules involved in mediating growth inhibition by interferon. In yeast, the corresponding protein binds to a specific site on the 26S rRNA. This gene is co-transcribed with the U42A, U42B, U101A, and U101B small nucleolar RNA genes, which are located in its third, first, second, and fourth introns, respectively. As is typical for genes encoding ribosomal proteins, there are multiple processed pseudogenes of this gene dispersed through the genome. [provided by RefSeq, Jul 2008], | hsa03010:Ribosome,hsa05171:Coronavirus disease - COVID-19, |
| 54778 | ring finger protein 111(RNF111) | The protein encoded by this gene is a nuclear RING-domain containing E3 ubiquitin ligase. This protein interacts with the transforming growth factor (TGF) -beta/NODAL signaling pathway by promoting the ubiquitination and proteosomal degradation of negative regulators, like SMAD proteins, and thereby enhances TGF-beta target-gene transcription. As a modulator of the nodal signaling cascade, this gene plays a critical role in the induction of mesoderm during embryonic development. Alternative splicing of this gene results in multiple transcript variants encoding distinct isoforms. [provided by RefSeq, Jul 2012], |  |
| 284996 | ring finger protein 149(RNF149) | Predicted to enable ubiquitin protein ligase activity. Predicted to be involved in ubiquitin-dependent protein catabolic process. Predicted to act upstream of or within cellular response to xenobiotic stimulus; negative regulation of MAPK cascade; and regulation of protein stability. Located in membrane. [provided by Alliance of Genome Resources, Apr 2022], |  |
| 91607 | schlafen family member 11(SLFN11) | Enables tRNA binding activity. Involved in several processes, including defense response to virus; negative regulation of G1/S transition of mitotic cell cycle; and replication fork arrest. Located in cytosol; nucleoplasm; and site of DNA damage. [provided by Alliance of Genome Resources, Apr 2022], |  |
| 9522 | secretory carrier membrane protein 1(SCAMP1) | This gene product belongs to the SCAMP family of proteins, which are secretory carrier membrane proteins. They function as carriers to the cell surface in post-golgi recycling pathways. Different family members are highly related products of distinct genes, and are usually expressed together. These findings suggest that these protein family members may function at the same site during vesicular transport rather than in separate pathways. A pseudogene of this gene has been defined on chromosome 1. Alternative splicing results in multiple transcript variants. [provided by RefSeq, Mar 2014], |  |
| 6402 | selectin L(SELL) | This gene encodes a cell surface adhesion molecule that belongs to a family of adhesion/homing receptors. The encoded protein contains a C-type lectin-like domain, a calcium-binding epidermal growth factor-like domain, and two short complement-like repeats. The gene product is required for binding and subsequent rolling of leucocytes on endothelial cells, facilitating their migration into secondary lymphoid organs and inflammation sites. Single-nucleotide polymorphisms in this gene have been associated with various diseases including immunoglobulin A nephropathy. Alternatively spliced transcript variants have been found for this gene. [provided by RefSeq, Oct 2009], | hsa04514:Cell adhesion molecules, |
| 22929 | selenophosphate synthetase 1(SEPHS1) | This gene encodes an enzyme that synthesizes selenophosphate from selenide and ATP. Selenophosphate is the selenium donor used to synthesize selenocysteine, which is co-translationally incorporated into selenoproteins at in-frame UGA codons. [provided by RefSeq, Sep 2010], | hsa00450:Selenocompound metabolism,hsa01100:Metabolic pathways, |
| 51765 | serine/threonine kinase 26(STK26) | The product of this gene is a member of the GCK group III family of kinases, which are a subset of the Ste20-like kinases. The encoded protein contains an amino-terminal kinase domain, and a carboxy-terminal regulatory domain that mediates homodimerization. The protein kinase localizes to the Golgi apparatus and is specifically activated by binding to the Golgi matrix protein GM130. It is also cleaved by caspase-3 in vitro, and may function in the apoptotic pathway. Several alternatively spliced transcript variants of this gene have been described, but the full-length nature of some of these variants has not been determined. [provided by RefSeq, Jul 2008], |  |
| 51246 | shisa family member 5(SHISA5) | This gene encodes a member of the shisa family. The encoded protein is localized to the endoplasmic reticulum, and together with p53 induces apoptosis in a caspase-dependent manner. Alternative splicing results in multiple transcript variants. Related pseudogenes of this gene are found on chromosome X. [provided by RefSeq, Apr 2016], | hsa04115:p53 signaling pathway, |
| 6775 | signal transducer and activator of transcription 4(STAT4) | The protein encoded by this gene is a member of the STAT family of transcription factors. In response to cytokines and growth factors, STAT family members are phosphorylated by the receptor associated kinases, and then form homo- or heterodimers that translocate to the cell nucleus where they act as transcription activators. This protein is essential for mediating responses to IL12 in lymphocytes, and regulating the differentiation of T helper cells. Mutations in this gene may be associated with systemic lupus erythematosus and rheumatoid arthritis. Alternate splicing results in multiple transcript variants that encode the same protein. [provided by RefSeq, Aug 2011], | hsa04217:Necroptosis,hsa04630:JAK-STAT signaling pathway,hsa04658:Th1 and Th2 cell differentiation,hsa05161:Hepatitis B,hsa05200:Pathways in cancer,hsa05321:Inflammatory bowel disease, |
| 6777 | signal transducer and activator of transcription 5B(STAT5B) | The protein encoded by this gene is a member of the STAT family of transcription factors. In response to cytokines and growth factors, STAT family members are phosphorylated by the receptor associated kinases, and then form homo- or heterodimers that translocate to the cell nucleus where they act as transcription activators. This protein mediates the signal transduction triggered by various cell ligands, such as IL2, IL4, CSF1, and different growth hormones. It has been shown to be involved in diverse biological processes, such as TCR signaling, apoptosis, adult mammary gland development, and sexual dimorphism of liver gene expression. This gene was found to fuse to retinoic acid receptor-alpha (RARA) gene in a small subset of acute promyelocytic leukemias (APLL). The dysregulation of the signaling pathways mediated by this protein may be the cause of the APLL. [provided by RefSeq, Jul 2008], | hsa04012:ErbB signaling pathway,hsa04062:Chemokine signaling pathway,hsa04217:Necroptosis,hsa04630:JAK-STAT signaling pathway,hsa04658:Th1 and Th2 cell differentiation,hsa04659:Th17 cell differentiation,hsa04917:Prolactin signaling pathway,hsa04933:AGE-RAGE signaling pathway in diabetic complications,hsa04935:Growth hormone synthesis, secretion and action,hsa05161:Hepatitis B,hsa05162:Measles,hsa05166:Human T-cell leukemia virus 1 infection,hsa05200:Pathways in cancer,hsa05203:Viral carcinogenesis,hsa05207:Chemical carcinogenesis - receptor activation,hsa05220:Chronic myeloid leukemia,hsa05221:Acute myeloid leukemia,hsa05223:Non-small cell lung cancer, |
| 6504 | signaling lymphocytic activation molecule family member 1(SLAMF1) | Enables SH2 domain binding activity and identical protein binding activity. Involved in several processes, including negative regulation of CD40 signaling pathway; negative regulation of cytokine production; and positive regulation of MAPK cascade. Located in extracellular exosome. [provided by Alliance of Genome Resources, Apr 2022], | hsa05162:Measles, |
| 64744 | small ArfGAP2(SMAP2) | Predicted to enable GTPase activator activity. Predicted to be involved in regulation of catalytic activity. Predicted to be located in cytoplasm. [provided by Alliance of Genome Resources, Apr 2022], | hsa04144:Endocytosis, |
| 114926 | small integral membrane protein 19(SMIM19) | Predicted to be integral component of membrane. [provided by Alliance of Genome Resources, Apr 2022], |  |
| 9990 | solute carrier family 12 member 6(SLC12A6) | This gene is a member of the K-Cl cotransporter (KCC) family. K-Cl cotransporters are integral membrane proteins that lower intracellular chloride concentrations below the electrochemical equilibrium potential. The proteins encoded by this gene are activated by cell swelling induced by hypotonic conditions. Alternate splicing results in multiple transcript variants encoding different isoforms. Mutations in this gene are associated with agenesis of the corpus callosum with peripheral neuropathy. [provided by RefSeq, Jul 2008], |  |
| 51312 | solute carrier family 25 member 37(SLC25A37) | SLC25A37 is a solute carrier localized in the mitochondrial inner membrane. It functions as an essential iron importer for the synthesis of mitochondrial heme and iron-sulfur clusters (summary by Chen et al., 2009 [PubMed 19805291]).[supplied by OMIM, Jan 2011], |  |
| 1318 | solute carrier family 31 member 2(SLC31A2) | Predicted to enable copper ion transmembrane transporter activity. Predicted to be involved in cellular copper ion homeostasis. Predicted to act upstream of or within regulation of copper ion transmembrane transport. Predicted to be located in membrane. Predicted to be active in plasma membrane. [provided by Alliance of Genome Resources, Apr 2022], |  |
| 29015 | solute carrier family 43 member 3(SLC43A3) | Predicted to enable transmembrane transporter activity. Predicted to be involved in transmembrane transport. Predicted to be integral component of membrane. [provided by Alliance of Genome Resources, Apr 2022], |  |
| 6533 | solute carrier family 6 member 6(SLC6A6) | This gene encodes a multi-pass membrane protein that is a member of a family of sodium and chloride-ion dependent transporters. The encoded protein transports taurine and beta-alanine. There is a pseudogene for this gene on chromosome 21. Alternative splicing results in multiple transcript variants. [provided by RefSeq, May 2013], |  |
| 29887 | sorting nexin 10(SNX10) | This gene encodes a member of the sorting nexin family. Members of this family contain a phox (PX) domain, which is a phosphoinositide binding domain, and are involved in intracellular trafficking. This protein does not contain a coiled coil region, like some family members. This gene may play a role in regulating endosome homeostasis. Alternative splicing results in multiple transcript variants. [provided by RefSeq, Dec 2010], |  |
| 26010 | spermatogenesis associated serine rich 2 like(SPATS2L) | Enables RNA binding activity. Located in cytosol; nucleolus; and nucleoplasm. Part of protein-containing complex. [provided by Alliance of Genome Resources, Apr 2022], |  |
| 54498 | spermine oxidase(SMOX) | Polyamines are ubiquitous polycationic alkylamines which include spermine, spermidine, putrescine, and agmatine. These molecules participate in a broad range of cellular functions which include cell cycle modulation, scavenging reactive oxygen species, and the control of gene expression. These molecules also play important roles in neurotransmission through their regulation of cell-surface receptor activity, involvement in intracellular signalling pathways, and their putative roles as neurotransmitters. This gene encodes an FAD-containing enzyme that catalyzes the oxidation of spermine to spermadine and secondarily produces hydrogen peroxide. Multiple transcript variants encoding different isoenzymes have been identified for this gene, some of which have failed to demonstrate significant oxidase activity on natural polyamine substrates. The characterized isoenzymes have distinctive biochemical characteristics and substrate specificities, suggesting the existence of additional levels of complexity in polyamine catabolism. [provided by RefSeq, Jul 2012], | hsa00330:Arginine and proline metabolism,hsa00410:beta-Alanine metabolism,hsa01100:Metabolic pathways, |
| 6850 | spleen associated tyrosine kinase(SYK) | This gene encodes a member of the family of non-receptor type Tyr protein kinases. This protein is widely expressed in hematopoietic cells and is involved in coupling activated immunoreceptors to downstream signaling events that mediate diverse cellular responses, including proliferation, differentiation, and phagocytosis. It is thought to be a modulator of epithelial cell growth and a potential tumour suppressor in human breast carcinomas. Alternatively spliced transcript variants encoding different isoforms have been found for this gene. [provided by RefSeq, Mar 2010], | hsa04064:NF-kappa B signaling pathway,hsa04072:Phospholipase D signaling pathway,hsa04151:PI3K-Akt signaling pathway,hsa04380:Osteoclast differentiation,hsa04611:Platelet activation,hsa04613:Neutrophil extracellular trap formation,hsa04625:C-type lectin receptor signaling pathway,hsa04650:Natural killer cell mediated cytotoxicity,hsa04662:B cell receptor signaling pathway,hsa04664:Fc epsilon RI signaling pathway,hsa04666:Fc gamma R-mediated phagocytosis,hsa05152:Tuberculosis,hsa05167:Kaposi sarcoma-associated herpesvirus infection,hsa05168:Herpes simplex virus 1 infection,hsa05169:Epstein-Barr virus infection,hsa05171:Coronavirus disease - COVID-19,hsa05203:Viral carcinogenesis, |
| 8631 | src kinase associated phosphoprotein 1(SKAP1) | This gene encodes a T cell adaptor protein, a class of intracellular molecules with modular domains capable of recruiting additional proteins but that exhibit no intrinsic enzymatic activity. The encoded protein contains a unique N-terminal region followed by a PH domain and C-terminal SH3 domain. Along with the adhesion and degranulation-promoting adaptor protein, the encoded protein plays a critical role in inside-out signaling by coupling T-cell antigen receptor stimulation to the activation of integrins. [provided by RefSeq, Jul 2008], | hsa04015:Rap1 signaling pathway, |
| 8935 | src kinase associated phosphoprotein 2(SKAP2) | The protein encoded by this gene shares homology with Src kinase-associated phosphoprotein 1, and is a substrate of Src family kinases. It is an adaptor protein that is thought to play an essential role in the Src signaling pathway, and in regulating proper activation of the immune system. This protein contains an amino terminal coiled-coil domain for self-dimerization, a plecskstrin homology (PH) domain required for interactions with lipids at the membrane, and a Src homology (SH3) domain at the carboxy terminus. Some reports indicate that this protein inhibits actin polymerization through interactions with actin assembly factors, and might negatively regulate the invasiveness of tumors by modulating actin assembly. Alternative splicing results in multiple transcript variants encoding different isoforms. [provided by RefSeq, Jan 2015], | hsa05135:Yersinia infection, |
| 6715 | steroid 5 alpha-reductase 1(SRD5A1) | Steroid 5-alpha-reductase (EC 1.3.99.5) catalyzes the conversion of testosterone into the more potent androgen, dihydrotestosterone (DHT). Also see SRD5A2 (MIM 607306).[supplied by OMIM, Mar 2008], | hsa00140:Steroid hormone biosynthesis,hsa01100:Metabolic pathways, |
| 2040 | stomatin(STOM) | This gene encodes a member of a highly conserved family of integral membrane proteins. The encoded protein localizes to the cell membrane of red blood cells and other cell types, where it may regulate ion channels and transporters. Loss of localization of the encoded protein is associated with hereditary stomatocytosis, a form of hemolytic anemia. There is a pseudogene for this gene on chromosome 6. Alternative splicing results in multiple transcript variants. [provided by RefSeq, Jul 2012], |  |
| 64420 | sushi domain containing 1(SUSD1) | Predicted to enable calcium ion binding activity. Predicted to be integral component of membrane. [provided by Alliance of Genome Resources, Apr 2022], |  |
| 23075 | switching B cell complex subunit SWAP70(SWAP70) | Enables cadherin binding activity. Predicted to be involved in regulation of actin polymerization or depolymerization. Predicted to act upstream of or within isotype switching. Located in actin cytoskeleton; cytoplasm; and plasma membrane. [provided by Alliance of Genome Resources, Apr 2022], |  |
| 116461 | tRNA splicing endonuclease subunit 15(TSEN15) | This gene encodes a subunit of the tRNA splicing endonuclease, which catalyzes the removal of introns from tRNA precursors. Alternative splicing results in multiple transcript variants. There is a pseudogene of this gene on chromosome 17. [provided by RefSeq, Jul 2014], |  |
| 54902 | tetratricopeptide repeat domain 19(TTC19) | This gene encodes a protein with a tetratricopeptide repeat (TPR) domain containing several TPRs of about 34 aa each. These repeats are found in a variety of organisms including bacteria, fungi and plants, and are involved in a variety of functions including protein-protein interactions. This protein is embedded in the inner mitochondrial membrane and is involved in the formation of the mitochondrial respiratory chain III. It has also been suggested that this protein plays a role in cytokinesis. Mutations in this gene cause mitochondrial complex III deficiency. Alternatively spliced transcript variants have been found for this gene. [provided by RefSeq, Sep 2012], |  |
| 125488 | tetratricopeptide repeat domain 39C(TTC39C) | Predicted to be involved in cilium assembly and otolith morphogenesis. [provided by Alliance of Genome Resources, Apr 2022], |  |
| 9473 | thymocyte selection associated family member 2(THEMIS2) | Predicted to be involved in T cell receptor signaling pathway and regulation of B cell activation. Predicted to be active in cytoplasm and nucleus. [provided by Alliance of Genome Resources, Apr 2022], |  |
| 7096 | toll like receptor 1(TLR1) | The protein encoded by this gene is a member of the Toll-like receptor (TLR) family which plays a fundamental role in pathogen recognition and activation of innate immunity. TLRs are highly conserved from Drosophila to humans and share structural and functional similarities. They recognize pathogen-associated molecular patterns (PAMPs) that are expressed on infectious agents, and mediate the production of cytokines necessary for the development of effective immunity. The various TLRs exhibit different patterns of expression. This gene is ubiquitously expressed, and at higher levels than other TLR genes. Different length transcripts presumably resulting from use of alternative polyadenylation site, and/or from alternative splicing, have been noted for this gene. [provided by RefSeq, Jul 2008], | hsa04620:Toll-like receptor signaling pathway,hsa05152:Tuberculosis, |
| 7097 | toll like receptor 2(TLR2) | The protein encoded by this gene is a member of the Toll-like receptor (TLR) family which plays a fundamental role in pathogen recognition and activation of innate immunity. TLRs are highly conserved from Drosophila to humans and share structural and functional similarities. This protein is a cell-surface protein that can form heterodimers with other TLR family members to recognize conserved molecules derived from microorganisms known as pathogen-associated molecular patterns (PAMPs). Activation of TLRs by PAMPs leads to an up-regulation of signaling pathways to modulate the host&apos;s inflammatory response. This gene is also thought to promote apoptosis in response to bacterial lipoproteins. This gene has been implicated in the pathogenesis of several autoimmune diseases. Alternative splicing results in multiple transcript variants. [provided by RefSeq, Jan 2016], | hsa04145:Phagosome,hsa04151:PI3K-Akt signaling pathway,hsa04613:Neutrophil extracellular trap formation,hsa04620:Toll-like receptor signaling pathway,hsa05132:Salmonella infection,hsa05134:Legionellosis,hsa05140:Leishmaniasis,hsa05142:Chagas disease,hsa05144:Malaria,hsa05145:Toxoplasmosis,hsa05146:Amoebiasis,hsa05152:Tuberculosis,hsa05161:Hepatitis B,hsa05162:Measles,hsa05168:Herpes simplex virus 1 infection,hsa05169:Epstein-Barr virus infection,hsa05170:Human immunodeficiency virus 1 infection,hsa05171:Coronavirus disease - COVID-19,hsa05205:Proteoglycans in cancer,hsa05235:PD-L1 expression and PD-1 checkpoint pathway in cancer,hsa05321:Inflammatory bowel disease,hsa05323:Rheumatoid arthritis,hsa05417:Lipid and atherosclerosis, |
| 51311 | toll like receptor 8(TLR8) | The protein encoded by this gene is a member of the Toll-like receptor (TLR) family which plays a fundamental role in pathogen recognition and activation of innate immunity. TLRs are highly conserved from Drosophila to humans and share structural and functional similarities. They recognize pathogen-associated molecular patterns (PAMPs) that are expressed on infectious agents, and mediate the production of cytokines necessary for the development of effective immunity. The various TLRs exhibit different patterns of expression. This gene is predominantly expressed in lung and peripheral blood leukocytes, and lies in close proximity to another family member, TLR7, on chromosome X. [provided by RefSeq, Jul 2008], | hsa04613:Neutrophil extracellular trap formation,hsa04620:Toll-like receptor signaling pathway,hsa05171:Coronavirus disease - COVID-19, |
| 26092 | torsin 1A interacting protein 1(TOR1AIP1) | This gene encodes a type 2 integral membrane protein that binds A- and B-type lamins. The encoded protein localizes to the inner nuclear membrane and may be involved in maintaining the attachment of the nuclear membrane to the nuclear lamina during cell division. Alternate splicing results in multiple transcript variants. [provided by RefSeq, Apr 2016], |  |
| 6938 | transcription factor 12(TCF12) | The protein encoded by this gene is a member of the basic helix-loop-helix (bHLH) E-protein family that recognizes the consensus binding site (E-box) CANNTG. This encoded protein is expressed in many tissues, among them skeletal muscle, thymus, B- and T-cells, and may participate in regulating lineage-specific gene expression through the formation of heterodimers with other bHLH E-proteins. Several alternatively spliced transcript variants of this gene have been described, but the full-length nature of some of these variants has not been determined. [provided by RefSeq, Jul 2008], |  |
| 6925 | transcription factor 4(TCF4) | This gene encodes transcription factor 4, a basic helix-loop-helix transcription factor. The encoded protein recognizes an Ephrussi-box (&apos;E-box&apos;) binding site (&apos;CANNTG&apos;) - a motif first identified in immunoglobulin enhancers. This gene is broadly expressed, and may play an important role in nervous system development. Defects in this gene are a cause of Pitt-Hopkins syndrome. In addition, an intronic CTG repeat normally numbering 10-37 repeat units can expand to &gt;50 repeat units and cause Fuchs endothelial corneal dystrophy. Multiple alternatively spliced transcript variants that encode different proteins have been described. [provided by RefSeq, Jul 2016], |  |
| 7086 | transketolase(TKT) | This gene encodes a thiamine-dependent enzyme which plays a role in the channeling of excess sugar phosphates to glycolysis in the pentose phosphate pathway. Alternatively spliced transcript variants encoding multiple isoforms have been observed for this gene. [provided by RefSeq, Apr 2012], | hsa00030:Pentose phosphate pathway,hsa01100:Metabolic pathways,hsa01200:Carbon metabolism,hsa01230:Biosynthesis of amino acids, |
| 10548 | transmembrane 9 superfamily member 1(TM9SF1) | Predicted to be involved in protein localization to membrane. Predicted to be located in autophagosome membrane; cytoplasmic vesicle; and lysosomal membrane. Predicted to be integral component of membrane. Predicted to be active in membrane. [provided by Alliance of Genome Resources, Apr 2022], |  |
| 7305 | transmembrane immune signaling adaptor TYROBP(TYROBP) | This gene encodes a transmembrane signaling polypeptide which contains an immunoreceptor tyrosine-based activation motif (ITAM) in its cytoplasmic domain. The encoded protein may associate with the killer-cell inhibitory receptor (KIR) family of membrane glycoproteins and may act as an activating signal transduction element. This protein may bind zeta-chain (TCR) associated protein kinase 70kDa (ZAP-70) and spleen tyrosine kinase (SYK) and play a role in signal transduction, bone modeling, brain myelination, and inflammation. Mutations within this gene have been associated with polycystic lipomembranous osteodysplasia with sclerosing leukoencephalopathy (PLOSL), also known as Nasu-Hakola disease. Its putative receptor, triggering receptor expressed on myeloid cells 2 (TREM2), also causes PLOSL. Multiple alternative transcript variants encoding distinct isoforms have been identified for this gene. [provided by RefSeq, Mar 2010], | hsa04380:Osteoclast differentiation,hsa04650:Natural killer cell mediated cytotoxicity, |
| 10972 | transmembrane p24 trafficking protein 10(TMED10) | This gene is a member of the EMP24/GP25L/p24 family and encodes a protein with a GOLD domain. This type I membrane protein is localized to the plasma membrane and golgi cisternae and is involved in vesicular protein trafficking. The protein is also a member of a heteromeric secretase complex and regulates the complex&apos;s gamma-secretase activity without affecting its epsilon-secretase activity. Mutations in this gene have been associated with early-onset familial Alzheimer&apos;s disease. This gene has a pseudogene on chromosome 8. [provided by RefSeq, Jul 2008], | hsa05130:Pathogenic Escherichia coli infection, |
| 23423 | transmembrane p24 trafficking protein 3(TMED3) | Predicted to be involved in Golgi organization; endoplasmic reticulum to Golgi vesicle-mediated transport; and intracellular protein transport. Located in Golgi apparatus; endoplasmic reticulum; and endoplasmic reticulum-Golgi intermediate compartment. [provided by Alliance of Genome Resources, Apr 2022], |  |
| 28978 | transmembrane protein 14A(TMEM14A) | Involved in negative regulation of mitochondrial outer membrane permeabilization involved in apoptotic signaling pathway. Located in endoplasmic reticulum membrane and mitochondrial membrane. [provided by Alliance of Genome Resources, Apr 2022], |  |
| 201799 | transmembrane protein 154(TMEM154) | Predicted to be integral component of membrane. [provided by Alliance of Genome Resources, Apr 2022], |  |
| 85363 | tripartite motif containing 5(TRIM5) | The protein encoded by this gene is a member of the tripartite motif (TRIM) family. The TRIM motif includes three zinc-binding domains, a RING, a B-box type 1 and a B-box type 2, and a coiled-coil region. The protein forms homo-oligomers via the coilel-coil region and localizes to cytoplasmic bodies. It appears to function as a E3 ubiquitin-ligase and ubiqutinates itself to regulate its subcellular localization. It may play a role in retroviral restriction. Multiple alternatively spliced transcript variants encoding different isoforms have been described for this gene. [provided by RefSeq, Dec 2009], | hsa03250:Viral life cycle - HIV-1,hsa05170:Human immunodeficiency virus 1 infection, |
| 84617 | tubulin beta 6 class V(TUBB6) | Predicted to enable GTP binding activity. Predicted to be a structural constituent of cytoskeleton. Predicted to be involved in microtubule cytoskeleton organization and mitotic cell cycle. Located in microtubule. [provided by Alliance of Genome Resources, Apr 2022], | hsa04145:Phagosome,hsa04540:Gap junction,hsa05010:Alzheimer disease,hsa05012:Parkinson disease,hsa05014:Amyotrophic lateral sclerosis,hsa05016:Huntington disease,hsa05020:Prion disease,hsa05022:Pathways of neurodegeneration - multiple diseases,hsa05130:Pathogenic Escherichia coli infection,hsa05132:Salmonella infection, |
| 203068 | tubulin beta class I(TUBB) | This gene encodes a beta tubulin protein. This protein forms a dimer with alpha tubulin and acts as a structural component of microtubules. Mutations in this gene cause cortical dysplasia, complex, with other brain malformations 6. Alternative splicing results in multiple splice variants. There are multiple pseudogenes for this gene on chromosomes 1, 6, 7, 8, 9, and 13. [provided by RefSeq, Jun 2014], | hsa04145:Phagosome,hsa04540:Gap junction,hsa05010:Alzheimer disease,hsa05012:Parkinson disease,hsa05014:Amyotrophic lateral sclerosis,hsa05016:Huntington disease,hsa05020:Prion disease,hsa05022:Pathways of neurodegeneration - multiple diseases,hsa05130:Pathogenic Escherichia coli infection,hsa05132:Salmonella infection, |
| 51465 | ubiquitin conjugating enzyme E2 J1(UBE2J1) | The modification of proteins with ubiquitin is an important cellular mechanism for targeting abnormal or short-lived proteins for degradation. Ubiquitination involves at least three classes of enzymes: ubiquitin-activating enzymes, or E1s, ubiquitin-conjugating enzymes, or E2s, and ubiquitin-protein ligases, or E3s. This gene encodes a member of the E2 ubiquitin-conjugating enzyme family. This enzyme is located in the membrane of the endoplasmic reticulum (ER) and may contribute to quality control ER-associated degradation by the ubiquitin-proteasome system. [provided by RefSeq, Jul 2008], | hsa04120:Ubiquitin mediated proteolysis,hsa04141:Protein processing in endoplasmic reticulum,hsa05012:Parkinson disease,hsa05022:Pathways of neurodegeneration - multiple diseases, |
| 7409 | vav guanine nucleotide exchange factor 1(VAV1) | This gene is a member of the VAV gene family. The VAV proteins are guanine nucleotide exchange factors (GEFs) for Rho family GTPases that activate pathways leading to actin cytoskeletal rearrangements and transcriptional alterations. The encoded protein is important in hematopoiesis, playing a role in T-cell and B-cell development and activation. The encoded protein has been identified as the specific binding partner of Nef proteins from HIV-1. Coexpression and binding of these partners initiates profound morphological changes, cytoskeletal rearrangements and the JNK/SAPK signaling cascade, leading to increased levels of viral transcription and replication. Alternatively spliced transcript variants encoding multiple isoforms have been observed for this gene. [provided by RefSeq, Apr 2012], | hsa04015:Rap1 signaling pathway,hsa04024:cAMP signaling pathway,hsa04062:Chemokine signaling pathway,hsa04510:Focal adhesion,hsa04650:Natural killer cell mediated cytotoxicity,hsa04660:T cell receptor signaling pathway,hsa04662:B cell receptor signaling pathway,hsa04664:Fc epsilon RI signaling pathway,hsa04666:Fc gamma R-mediated phagocytosis,hsa04670:Leukocyte transendothelial migration,hsa04810:Regulation of actin cytoskeleton,hsa05135:Yersinia infection,hsa05205:Proteoglycans in cancer,hsa05417:Lipid and atherosclerosis, |
| 9839 | zinc finger E-box binding homeobox 2(ZEB2) | The protein encoded by this gene is a member of the Zfh1 family of 2-handed zinc finger/homeodomain proteins. It is located in the nucleus and functions as a DNA-binding transcriptional repressor that interacts with activated SMADs. Mutations in this gene are associated with Hirschsprung disease/Mowat-Wilson syndrome. Alternatively spliced transcript variants have been found for this gene.[provided by RefSeq, Jan 2010], | hsa05206:MicroRNAs in cancer, |
| 54680 | zinc finger HIT-type containing 6(ZNHIT6) | Enables ATPase binding activity; TFIID-class transcription factor complex binding activity; and identical protein binding activity. Involved in box C/D snoRNP assembly; protein complex oligomerization; and snoRNA localization. Located in extracellular exosome. Part of pre-snoRNP complex. [provided by Alliance of Genome Resources, Apr 2022], |  |
| 9202 | zinc finger MYM-type containing 4(ZMYM4) | Predicted to enable DNA binding activity. Involved in cytoskeleton organization and regulation of cell morphogenesis. [provided by Alliance of Genome Resources, Apr 2022], |  |
| 7739 | zinc finger protein 185 with LIM domain(ZNF185) | Zinc-finger proteins bind nucleic acids and play important roles in various cellular functions, including cell proliferation, differentiation, and apoptosis. This gene encodes a LIM-domain zinc finger protein. The LIM domain is composed of two contiguous zinc finger domains, separated by a two-amino acid residue hydrophobic linker. The LIM domain mediates protein:protein interactions. Multiple alternatively spliced transcript variants encoding different isoforms have been identified.[provided by RefSeq, May 2010], |  |
| 23528 | zinc finger protein 281(ZNF281) | Enables DNA-binding transcription repressor activity, RNA polymerase II-specific and RNA polymerase II cis-regulatory region sequence-specific DNA binding activity. Involved in negative regulation of gene expression; negative regulation of transcription by RNA polymerase II; and positive regulation of transcription, DNA-templated. Located in nucleoplasm. [provided by Alliance of Genome Resources, Apr 2022], |  |
