# Supplementary material for: The Variation of Transcriptomic Perturbations is Associated with the Development and Progression of Various Diseases
Source: Dis Markers. 2022 Sep 26;2022:2148627. doi: 10.1155/2022/2148627 (PMC9530920; doi:10.1155/2022/2148627)
Supplement: Supplementary 3 — Table S3: the genes displayed significant expression perturbations' correlations with VTP values across diseases and had genetic variants statistically associated with a risk of the diseases. [file 2148627.f3.docx]

| **Table S3. The genes displayed significant expression perturbations’ correlations with VTP values across diseases and had genetic variants statistically associated with a risk of the diseases** | |
| --- | --- |
| **Disease** | **Intersection genes** |
| Alzheimer's disease | *RDX, PIP4K2A, PILRA, LPXN, LILRB2, ITGAX, IQGAP2, FOXN2, CR1, CELF2, CDC42SE2, CD2AP, PDK4, PARP8, HSPA6, BNIP3* |
| Schizophrenia | *SPATS2L, MPHOSPH9, MSI2, TCF4, GNG7, NRIP1, ZEB2, NAB1, PPP3R1, ARHGAP31, QPCT, PRKCB, PIK3C2A, ASAP1, SKAP1, RNF111, FARSB, SEPHS1, TUBB, HLA-DMA, SLC6A6, GMIP, SLAMF1, RBKS, ETS2, RASA3, ZMYM4, LGALS3, BNIP3, FOXN2* |
| Corona Virus Disease 2019 | *CCR1, CPQ, IFNAR2, LMNB1, MYO1F, PLSCR1, PTPRE, STK26* |
| Malaria | *SMYD3* |
| Cardiovascular disease | *TKT, TCF4, SWAP70, DDHD2, ARHGAP31, LTB* |
| Respiratory disease | *DENND2D, PELI1, PLXNC1* |
| Liver disease | *TCF4, STAT4, NAB1, IKZF3, OLFM1, SMYD3, PRKCB, BCL2A1, SPATS2L, RAB31, RASGRP1, ITGAL, RDX, CXCR2, AHR, SKAP2, IFNGR2, LEPROT, CD28, LPXN, CCL2, HSPA6* |
| Kidney disease | *PBX3, KIAA0319L, STAT4, ITGAM, IKZF3, NAB1, TLR1, HLA-DMA, PTPRC, ZEB2, ITGAX, DOCK10, CD247, ARHGAP31, RGS19, NRIP1, LBH, SKAP2, NOTCH2, SMYD3, IPCEF1, ACAP1, ELF1, RASGRP1, PRKCB, TTC39C, ICAM3, HSPA6, ESYT1, CD28* |
| Endocrine disease | *PAM, ACSL1, ACAP1, NOTCH2, PTPRC, SKAP2, RASGRP1, STAT4, MNDA, TLR1, AHR, IKZF3, CCND2, ARAP1, CPQ, TCF4, TCF12, ATP8B2, PARP8, IL13RA1, GCA, TMEM154, CYTH1, LDHB, ZEB2, EVI2B, ANXA5, SYK, SRBD1, MPHOSPH9, IPCEF1, QKI, NUDT5, RDX, COQ10A, PFKFB3, NCOA1, TSEN15, NMI, IFNAR2, GPR137B* |
